# Validation and analysis of 12,000 AI-driven CAR-T designs in the *Bits to Binders* competition

**DOI:** 10.1038/s44320-026-00246-1

**Published:** 2026-10-08

**Authors:** Clayton W Kosonocky, Alex M Abel, Aaron L Feller, Amanda E Cifuentes Rieffer, Phillip R Woolley, Jakub Lála, Daryl R Barth, Tynan Gardner, Bits to Binders Competitors, Daniel Acosta, Stefano Angioletti-Uberti, Vinayak Annapure, Neil Anthony, Rana A Barghout, Max Beining, Parth Bibekar, Dionessa Biton, Patrick Bryant, Anton Bushuiev, Cianna Calia, Ava Chan, Jie Chen, Akshay Chenna, Nicole Chiang, Vivian Chu, Joseph Clark, Alia Clark-ElSayed, Elliot Cole, Qian Cong, Shreyasi Das, Tanner Dean, Tyler Derr, Alejandro Diaz, Jesse Durham, Moritz Ertelt, Antonio Fonseca, Ora Furman, Tynan Gardner, Jokent Gaza, Nathaniel Greenwood, Rui Guo, Abel Gurung, Vivian Haas, Seyed Alireza Hashemi, Dieter Hoffmann, Sofiia Hoian, Corey Howe, Calvin XiaoYang Hu, Jinling Huang, Nader Ibrahim, Satoshi Ishida, Ansar Ahmad Javed, Aswini Javvadi, Roman Jerala, Daksh Joshi, Even Kiely, Haelyn Kim, Diego Kleiman, Sarah Knapp, Huan Koh, Petr Kouba, Yong Youn Kwon, Duško Lainšček, Jakub Lála, Hakyung Lee, Qiuzhen Li, Max Lingner, Yuxuan Liu, Yunchao Liu, Qianchen Liu, Ajasja Ljubetič, Marko Ludaic, Karl Lundquist, Ashish Makani, Javier Marchena-Hurtado, Freddie Martin, Jens Meiler, Fernando Meireles, David Miller, Nuria Mitjavila, Tjaša Mlakar, Rajarshi Mondal, Saeideh Moradvandi, Rocco Moretti, Sai Jaideep Reddy Mure, Pragati Naikare, Fatemeh Nasiri, Kaveh Nasrollahi, Yisel Martinez Noa, Joseph Openy, Aoi Otani, Vikrant Parmar, Jimin Pei, Alberto Perez, Rajat Punia, Francisco Requena, Dominic Rieger, Aparna Sahu, Elias Sanchez, Rohit Satija, Tadej Satler, Tom Schlegel, Yang Shen, Diwakar Shukla, Rangana De Silva, Benedikt Singer, Arjun Singh, Amardeep Singh, Bhumika Singh, Jinwoong Song, Harish Srinivasan, Hannah Stewart, Zhaoqian Su, Julia Varga, Xin Wang, Dion Whitehead, Matthew Williams, Max Witwer, Rujie Yin, Song Yin, Rongqing Yuan, Nikola Zadorozhny, Dishant Parag Zaveri, Genhui Zheng, Yizhen Zheng, Jian Zhou, Shaowen Zhu, Lin Zhu, Stephen C Ekker, Andrew D Ellington, Wesley A Wierson, Edward M Marcotte

**Affiliations:** 1 Department of Molecular Biosciences, University of Texas at Austin, Austin, TX USA; 2 The BioML Society, Austin, TX USA; 3 LifEngine Animal Health (LEAH) Laboratories Incorporated, Minneapolis, MN USA; 4 Department of Materials, Imperial College London, London, United Kingdom; 5 Department of Pediatrics, Dell Medical School, University of Texas at Austin, Austin, TX USA; 6 University of Texas at Austin, Austin, TX USA; 7 Imperial College London, London, UK; 8 Monash University, Melbourne, VIC Australia; 9 University of Toronto, Toronto, ON Canada; 10 Institute for Drug Discovery, Leipzig University, Leipzig, Germany; 11 EPFL, Lausanne, Switzerland; 12 University of Oldenburg, Oldenburg, Germany; 13 Stockholm University, Stockholm, Sweden; 14 Czech Technical University in Prague, Prague, Czechia; 15 University of California San Diego, San Diego, CA USA; 16 University of Oxford, Oxford, UK; 17 University of Texas Southwestern Medical Center, Dallas, TX USA; 18 Virtue Therapeutics, Gurugram, India; 19 University of Illinois Urbana-Champaign, Champaign, IL USA; 20 McGill University, Montreal, QC Canada; 21 Sungkyunkwan University, Suwon, South Korea; 22 Vanderbilt University, Nashville, TN USA; 23 Uppsala University, Uppsala, Sweden; 24 Gensaic, Cambridge, MA USA; 25 Hebrew University of Jerusalem, Jerusalem, Israel; 26 University of Florida, Gainesville, FL USA; 27 University of Washington, Seattle, WA USA; 28 Purdue University, West Lafayette, IN USA; 29 University of Tehran, Tehran, Iran; 30 Taras Shevchenko National University of Kyiv, Kyiv, Ukraine; 31 Myriad Genetics, Salt Lake City, UT USA; 32 Harvard University, Cambridge, MA USA; 33 Aibio AB, Klagshamn, Sweden; 34 Boltzmann Labs, Hyderabad, India; 35 National Institute of Chemistry, Ljubljana, Slovenia; 36 Seoul National University, Seoul, South Korea; 37 Texas A&M University, College Station, TX USA; 38 Charité – Universitätsmedizin Berlin, Berlin, Germany; 39 University of Copenhagen, Copenhagen, Denmark; 40 IIIT Delhi, New Delhi, India; 41 TU Dortmund, Dortmund, Germany; 42 Weill Cornell Medicine, New York, NY USA; 43 Pivotal Life Sciences, San Francisco, CA USA; 44 University of Pittsburgh, Pittsburgh, PA USA; 45 University of Michigan, Ann Arbor, MI USA; 46 Astera Institute, Emeryville, CA USA; 47 Northwestern University, Evanston, IL USA

**Keywords:** Biotechnology & Synthetic Biology, Immunology

## Abstract

Artificial intelligence (AI) methods for proteins have advanced rapidly, improving structure prediction and design, particularly for de novo binders. However, most evaluations emphasize binding affinity rather than higher-order biological function. We present *Bits to Binders*, a global competition evaluating de novo binder design in the context of chimeric antigen receptor (CAR) T cells. Teams from 42 countries submitted 12,000 designs of 80-amino acid de novo minibinders targeting human CD20 as CAR binding domains. Designs were screened by pooled CAR-T proliferation, identifying 707 designs exhibiting significant CD20-specific enrichment, with team hit rates from 0.6% to 38.4%. Top-performing candidates were validated as individual constructs, measuring CD20-specific proliferation, expansion, cytokine production, and targeted cell lysis. We identified common design methodologies and factors correlated with DNA synthesis, expression, and target-specific T cell activation which nearly double the success rates when applied as a retrospective filter. We release this dataset as an open resource, with practical recommendations to support more effective AI-driven binder design.

## Synopsis

A global competition benchmarked 12,000 AI-designed CD20 binders in primary human CAR-T cells, linking design strategies and sequence features to a variety of antigen independent and dependent outcomes to help inform future protein design.

- Twenty-eight teams applied various AI-driven workflows to design 12,000 putative CD20 binders for functional evaluation as CAR recognition domains.
- Pooled screening identified 707 designs with antigen-dependent enrichment, and individual assays validated CAR-T cell activity for 7 of the 10 leading designs.
- Functional outcomes were associated with design methodology and sequence features that affect DNA synthesis, cellular recovery and target-dependent activity.
- Retrospective sequence filters identified common failure modes and nearly doubled the proportion of designs exhibiting antigen-dependent proliferation.

## Abstract (web summary)

A global competition benchmarked 12,000 AI-designed CD20 binders in primary human CAR-T cells, linking design strategies and sequence features to a variety of antigen independent and dependent outcomes to help inform future protein design.

## Graphical abstract

**Figure:**
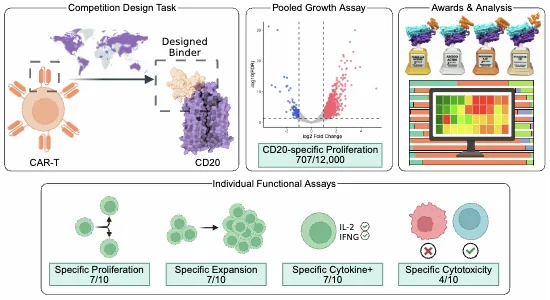

## Introduction

The application of artificial intelligence (AI) methods to proteins has shown remarkable success in recent years (Koh et al, 2025). One application area, protein binder design, has garnered significant interest due to its demonstrated tractability and potential to create antibody-like therapeutics (Fox et al, 2025). Many AI-driven models have been validated on their ability to design proteins that bind to arbitrary targets, with reported success rates exceeding 10% of tested designs (Kosonocky et al, 2026). Despite this progress, the best practices for protein binder design are not yet standardized, and it is unclear which computational approaches are most effective across diverse contexts.

Regularly occurring benchmarks like the Critical Assessment of Structure Prediction (CASP) and the Critical Assessment of Predicted Interactions (CAPRI) have been essential in advancing protein structure prediction models to their current level of performance (Pereira et al, 2021; Janin et al, 2003). Similar efforts are emerging in AI-driven protein design, such as the Protein Engineering Tournament and Adaptyv Bio’s EGFR binder challenge, which validate crowd-sourced designs against measurable objectives such as binding affinity and activity, providing insight into method performance (Armer et al, 2025; Cotet et al, 2025). While these competitions have been instrumental in assessing AI-driven tools for designing proteins that bind, the suitability of designs for other functional endpoints remains largely unknown.

Binding is one of several important features of immune function, and engineered binding by Chimeric Antigen Receptor (CAR) T Cells is of great therapeutic importance (Larson and Maus, 2021). CAR-T cells are derived from a patient’s normal T cells through the introduction and expression of a chimeric T cell receptor, where the extracellular domain enables the recognition of a targeted tumor surface antigen (Jayaraman et al, 2020; June et al, 2018). Upon target engagement by that antigen, receptor clustering occurs, and downstream pathways are activated that drive T cell proliferation, cytokine secretion, and cytotoxic killing.

Prior work has demonstrated the general feasibility of AI-designed CAR binding domains (Broske et al, 2025; Mergen et al, 2024; Johansen et al, 2025; Chow et al, 2025), and thus, this system provides an excellent opportunity for evaluating different design approaches in a competition setting. Designing binders in the context of a CAR-T cell requires construct expression and membrane localization of the receptor that is compatible with cell viability and engages downstream signaling upon target recognition, leading to proliferation, expansion, cytokine production, and target cell lysis. We created the *Bits to Binders* competition, which engaged a total of 28 teams to generate 12,000 80-amino-acid de novo protein minibinders that targeted the lymphoma surface antigen CD20, to be synthesized and assayed for CAR-T cell function. From the resulting dataset, we extracted design principles and predictive metrics that contribute to functional binder performance in the context of a CAR-T cell therapy, with the goal of moving the field closer to standardized and effective methods for AI-driven protein design.

## Results

### Crowd-sourcing de novo-designed CD20-binding CARs for the *Bits to Binders* competition

**Figure 1:**
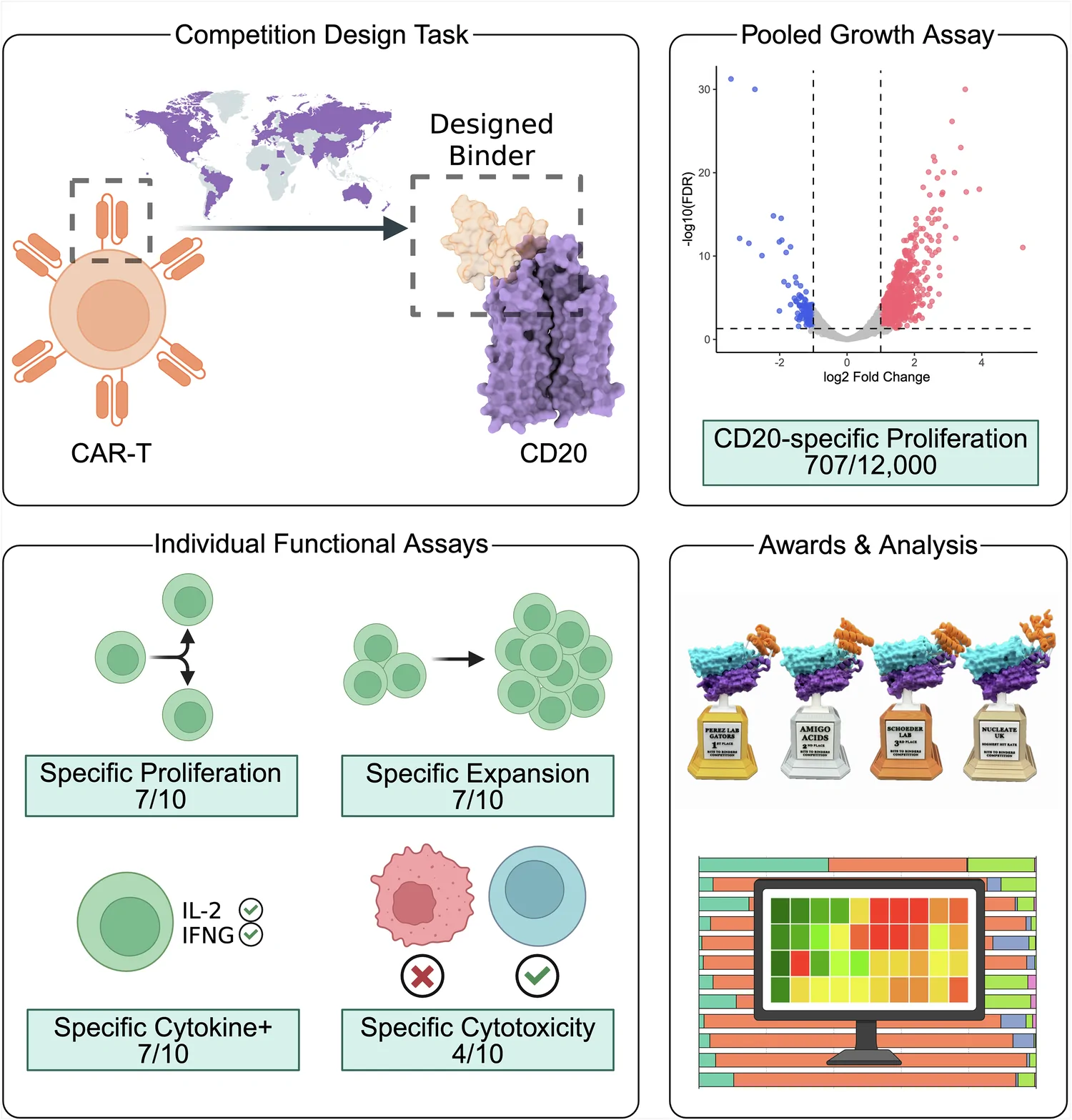
Overview of the *Bits to Binders* competition. Competitors from around the world used AI-driven protein design methods to design 12,000 80-amino-acid de novo protein minibinders that bind CD20 and initiate a T cell immune response when used as the binding domain of a CAR-T cell. The 12,000 designs were tested in a competitive growth assay, in which CD20-specific CAR-T cell proliferation was measured. The designs with the greatest CD20-specific fold change in growth were tested individually for CD20-specific proliferation, expansion, cytokine production, and target cell lysis. The results were then analyzed to identify computational features leading to increased success rates for AI-driven CAR design. Figure 1 was created using BioRender (https://biorender.com).

Competitors from 42 countries were tasked to use their chosen AI-driven protein design methods to design 500 80-amino-acid binders to the extracellular domain of human CD20 that could be accommodated in the context of a CAR (Fig. 1; Appendix Figs. S23, S24, and S26). This task differed from a pure binder design task in several ways. First, while the competitors submitted only binding domains, they were aware that these should eventually be compatible with the greater CAR scaffold, in particular, the transmembrane domain. Second, success was dependent on achieving sufficient affinity to effectively recognize the target, but not so much that the CAR-T cell becomes exhausted (Zebley and Youngblood, 2022; Benzaoui et al, 2022). Third, teams were tasked to design minibinders of exactly 80 amino acids, much shorter than a typical scFv (230–260 amino acids). And finally, success would ultimately depend on CAR-T function, not just binding. These nuances, along with a primer on CAR-T biology, the exact CAR scaffold used, and an in-depth explanation of all assays, were conveyed to the participants during the solicitation (Appendix Information C). In addition, several research and review articles on AI-driven protein design, CAR-T therapies, and CD20 biology were provided to the participants (Appendix Information C). The format of this study, being a competition evaluating AI-driven protein design tools, carries inherent caveats: the goal is to assess how well current methods design proteins in a therapeutic context rather than to produce a clinical-ready therapeutic; designs are tested within a single CAR scaffold for comparability and scalability; and method usage is uncontrolled, drawn from a crowd-sourced pool in which dominant pipelines risk skewing downstream analysis. That said, the competition format invited methodological creativity that might not otherwise have been explored, while serving as a learning and collaboration exercise for scientists around the world (Appendix Information E). Organizational lessons from running the competition are summarized in Table 1 (further discussion in Appendix Information C).

**Table 1:** Organizational lessons and recommendations derived from running the *Bits to Binders* competition.

**Build the task around a scalable assay that you have already performed**. We only began planning the competition and task after partnering with LEAH Labs and learning of their existing scalable assay. We think this was a major factor in our success as we had full confidence throughout in being able to provide experimental results to the competitors.
**Ensure that you have the money and resources for the event before announcing**. As an organizer, your main goal is to ensure that the competition finishes and the competitors feel satisfied with having participated. One failure mode that can occur is that you run out of money or resources, and so this should be mitigated prior to establishing public expectations.
**The task should be doable, but it shouldn’t be trivially easy**. Prior to forming the competition, LEAH had evidence that this task was possible, though at a 1/1000 success rate. The task being possible can help encourage competitors to see it through, and the task being not trivially easy can provide excitement about tackling a hard problem.
**The task should answer a question that you personally want to know**. We at the BioML Society wanted to know which protein design workflows worked best in real-world protein design, and so we decided to host a competition to find out. This allowed us organizers to be interested in the problem throughout and convey this interest to the participants. Moreover, our own interest in this problem was a sign that others might be interested too.
**Decide if you want to do a prospective or retrospective competition**. Our competition was a prospective competition, in which we ordered and evaluated the designs after they had been submitted. This aligned well with the competition being generative, as we could not have planned in advance which sequences people would design. This is in contrast to a “Kaggle-style” retrospective competition in which people compete to predict outcomes from an already-measured set. The latter works better for regression- and classification-based tasks.
**Reach out to industry and academic partners to help sponsor the event**. We had sponsors cover most of the competition’s supplies and labor costs. In exchange, we advertised their company and gave them time to speak about their products during the kickoff event.
**Simplify the logistics as much as possible**. We initially wanted to do an in-person kickoff event in Austin, Texas. However, this would have required us to reserve an event space, provide catering, and help coordinate travel. This was outside our organizational capacity, so we simplified it to a virtual kickoff event.
**If you provide compute, it should be as easy to use as possible**. We provided node hours to competitors for an ARM64 system that ended up not being used because many of the packages were not yet compatible.
**Allow competitors to assemble their own teams**. We created a Slack workspace for competitors to join and create teams. Many of the teams that submitted in the end were formed in this manner, and this mechanism served to establish relationships between researchers around the world.

At the end of the 6-week design stage, a total of 12,000 designs were collected. The approaches used spanned a wide range of generative design and selection strategies (Appendix Information E). Generative diffusion models were the most common approach, with 18 teams using RFdiffusion (Watson et al, 2023) and two teams using sequence-structure co-design models like Chroma (Ingraham et al, 2023). The non-diffusion approaches included constraint- or hallucination-based design such as BindCraft (Pacesa et al, 2025) (three teams), EvoBind (Li et al, 2024; Bryant and Elofsson, 2022) (2), and ColabDesign (3), as well as protein language models like PepMLM (Chen et al, 2024) (1), ESM-2 (Lin et al, 2023) (2), and RayGun (Devkota et al, 2024) (1). Fixed backbone design tools were widely used for sequence generation and diversification, notably ProteinMPNN (Dauparas et al, 2022) (19 teams) followed by SolubleMPNN (Goverde et al, 2023) (2) and CARBonAra (Krapp et al, 2024) (1). Twelve teams generally followed the default RFdiffusion–ProteinMPNN–AlphaFold2 pipeline, while nine teams opted to perform “iterative diffusion”, in which the initial binder was generated, the low-scoring segments were removed, and the process was repeated until the scores were sufficient.

Many teams conditioned their generative models on CD20 in the Rituximab-bound state (PDB: 6VJA) or by starting with fragments of CD20-specific antibodies. Initial structures were frequently refined using FastRelax (Tyka et al, 2011), GROMACS (Van Der Spoel et al, 2005), or OpenMM (Eastman et al, 2017). After generation, it was common to select sequences using scores, structure prediction models, or other bioinformatics tools. To confirm that generated sequences were predicted to fold into the desired structures, AlphaFold2 (Jumper et al, 2021) was used by 17 teams, with others using ColabFold (Mirdita et al, 2022) (4), ESMFold (Lin et al, 2023) (4), Chai-1 (Boitreaud et al, 2024) (1), or HelixFold (Liu et al, 2024) (1). Interestingly, only one team reported folding their designs in the context of the entire CAR sequence. Other selection criteria included Rosetta energy scores (Leman et al, 2020; Leaver-Fay et al, 2011), HDOCK scores (Yan et al, 2020), and binding affinity predictions from Prodigy (Xue et al, 2016). The wide variety of inputs, model types, and scoring strategies resulted in substantial sequence diversity between teams: over 83% of submissions did not produce a significant MMseqs2 (Steinegger and Söding, 2017) alignment to sequences from other teams (Appendix Fig. S1). In contrast, within-team sequence redundancy was relatively high, with 20% of sequences aligning nearly perfectly to another design from the same team (≥0.95 MMseqs2 identity).

### Screening 12,000 de novo CAR binding domains in a T cell proliferation assay

**Figure 2:**
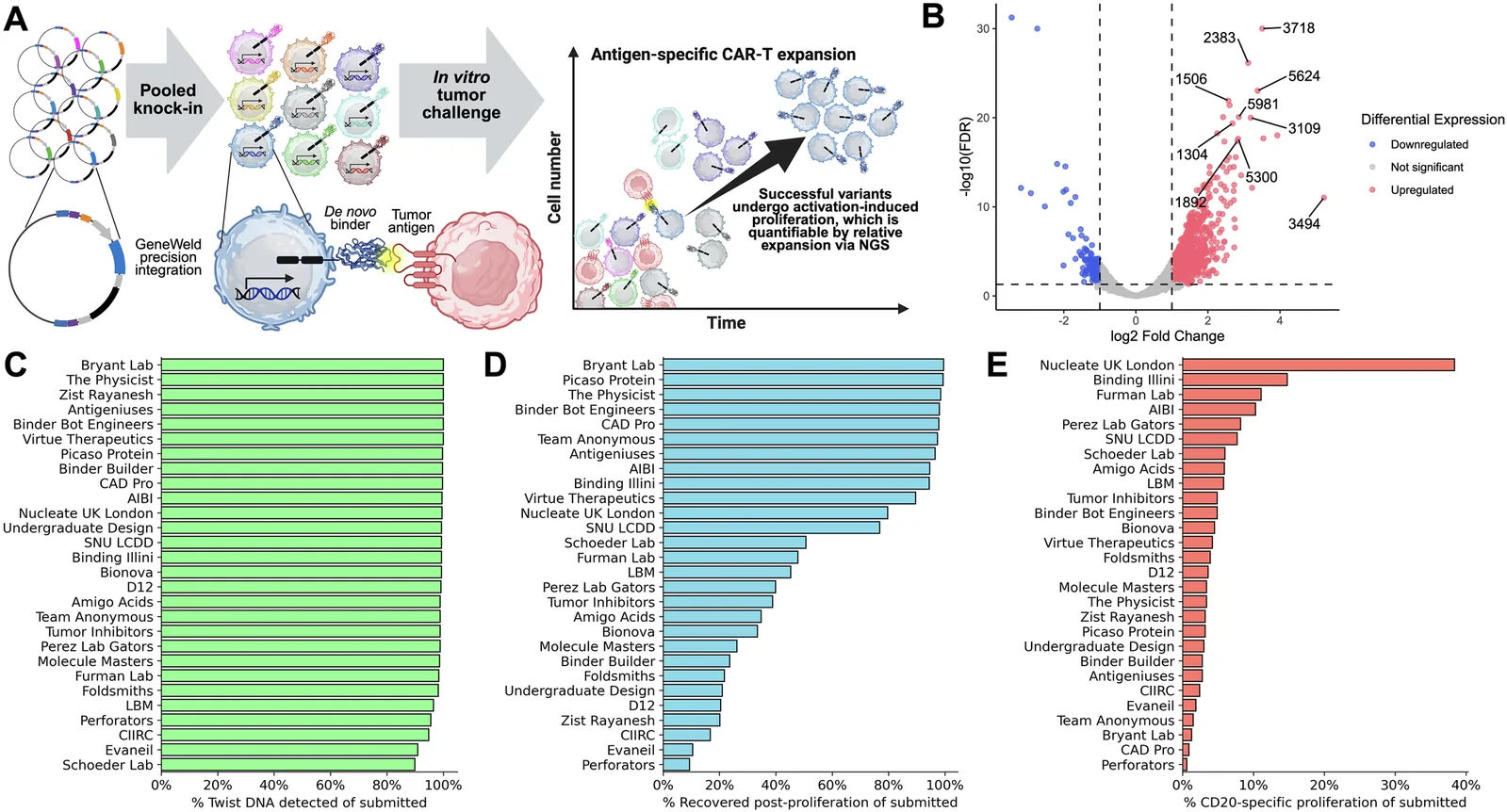
Pooled proliferation assay identifies designs with CD20-specific CAR-T cell response. (**A**) Transgenes for the de novo minibinder-based CARs are knocked into a population of T cells, which is then subjected to either a tumor challenge or a control condition. Target-specific T cell proliferation indicates antigen recognition and is quantified as the relative change in abundance of each transgene sequence in the tumor challenge condition compared with the control. (**B**) Of 12,000 binders, 707 showed a significant CD20-specific ≥2-fold increase in growth, indicating potential recognition of CD20 by the binder. Statistically significant hits were determined using FDR-controlled edgeR on three technical replicates per condition from one donor cell line. (**C**) Percent of each team’s submitted sequences detected in the plasmid pool during Twist quality control. (**D**) Percent of each team’s submitted sequences with ≥25 reads in each of the six proliferation assay replicates. Post-proliferation recovery suggests that the binder produced a viable CAR-T cell. (**E**) Percent of each team’s submitted sequences with a significant ≥2-fold change in CD20-specific proliferation. Panel (**A**) was created using BioRender (https://biorender.com). Source data are available online for this figure.

To functionally evaluate the 12,000 binder designs, we employed a pooled CAR-T challenge assay that measures antigen-dependent T cell proliferation in primary T cells from a single clinical isolate (Fig. 2A). Pools of codon-optimized DNA fragments encoding the 80-amino-acid binding domain were synthesized and cloned into a plasmid encoding a second-generation CAR backbone by replacing the binder region of a second-gen CAR28z construct (Methods). The plasmid library was used as a DNA donor for targeted integration of the CAR at the T cell receptor alpha chain (TRAC) to engineer a pool of TRAC(−), CAR(+) T cells (Webber et al, 2023; Wierson et al, 2020).

The sequence of the integrated, designed binder in each CAR construct served as its own unique DNA barcode for each cell. To enrich for T cells carrying integrations of the pooled CAR library and negatively select unedited T cells, we subjected the population to methotrexate selection (Ng, 2012; Balke-Want et al, 2023) via the co-expressed DHFR^F/S^ variant. After selection and expansion, the remaining CAR-expressing cells were co-cultured either alone or with the CD20-positive Raji cell line (Dorvignit et al, 2012), both in triplicate. Enrichment of binders was measured by the relative change in “barcode” sequences after two weeks in Raji co-culture compared to those that were cultured without CD20+ targets. Enrichment or depletion in the non-target group may be indicative of dysregulation in CAR-T signaling or decreased cellular fitness independent of binding. Thus, comparing the Raji co-culture with this group allows the effect of CD20+ targets on proliferation to be robustly quantified.

NGS reads were quantified across the three technical replicates to identify significant CD20-specific enrichment (Fig. 2B, Methods), with few jackpot effects occurring between the replicates (Appendix Fig. S2). Across the 12,000 designs, 98.3% passed Twist’s DNA quality check (Fig. 2C), but only 56.8% passed the minimum read thresholds after growth. Given that a functional CAR is required to provide the basal signaling required for sustained T cell fitness and survival, this drop-off indicates that many of the designs may have failed to yield a valid receptor (Fig. 2D).

Ultimately, 707 clones showed significant CD20-specific enrichment of at least twofold relative to controls. Hit rates varied substantially between teams, ranging from 0.6 to 38.4% of submitted sequences (Fig. 2E). The top ten non-redundant designs were chosen to proceed to a series of individual T cell functional assays.

### Confirming function with individual T cell assays

**Figure 3:**
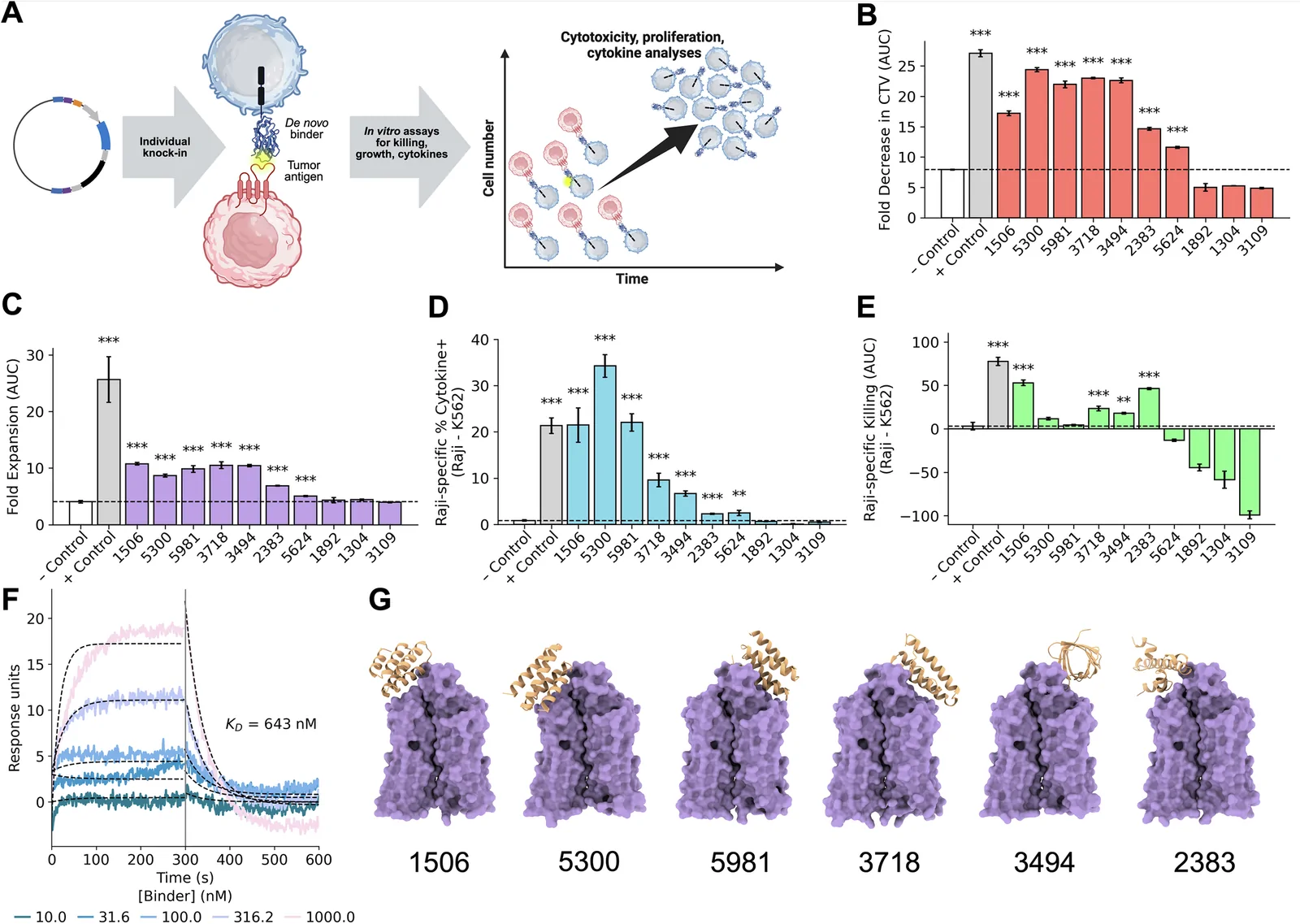
In vitro assays confirm designed binders function as CAR-T cells. (**A**) DNA constructs of designed de novo minibinder-based CARs are integrated into primary T cells for functional analysis against CD20+ tumor cells. (**B**) CD20-specific proliferation, computed by AUC of CTV dilution (Raji − K562) across four E:T ratios (Fig. EV2). Bars show mean CTV-MFI ± SE; significance by BH-corrected one-sided Welch’s t-test vs. negative control. Significance stars relative to negative controls: *p < 0.05; **p < 0.01; ***p < 0.001; *n* = 3 technical replicates. Exact BH-corrected *p* values are as follows: *p*(–Control): 1.00E + 00, *p*(+Control): 2.58E-237, p(1506): 2.24E-127, p(5300): 0.00E + 00, p(5981): 8.93E-132, p(3718): 0.00E + 00, p(3494): 2.51E-289, p(2383): 4.51E-157, p(5624): 2.59E-84, p(1892): 1.00E + 00, p(1304): 1.00E + 00, p(3109): 1.00E + 00. (**C**) CD20-specific expansion, computed as AUC of fold-expansion (Raji − K562) across four E:T ratios (Fig. EV3). Bars show mean ± SE; BH-corrected one-sided Welch’s *t*-test vs. negative control. Significance stars relative to negative controls: *p < 0.05; **p < 0.01; ***p < 0.001; *n* = 3 technical replicates. Exact BH-corrected *p* values are as follows: *p*(–Control): 1.00E + 00, *p*(+Control): 8.44E-08, *p*(1506): 2.07E-85, p(5300): 2.52E-36, *p*(5981): 1.80E-19, *p*(3718): 2.28E-24, *p*(3494): 1.53E-83, *p*(2383): 3.13E-27, *p*(5624): 2.27E-04, *p*(1892): 3.68E-01, *p*(1304): 1.22E-01, *p*(3109): 6.71E-01. (**D**) CD20-specific production of IL2 and IFNγ. Bars show mean percent of cytokine+ cells in the vs-Raji condition ± SE; BH-corrected one-sided Welch’s *t*-test vs. negative control. Significance stars relative to negative controls: *p < 0.05; **p < 0.01; ***p < 0.001; *n* = 3 technical replicates. Exact BH-corrected *p* values are as follows: *p*(–Control): 1.00E + 00, *p*(+Control): 1.01E-33, *p*(1506): 3.15E-08, *p*(5300): 1.31E-41, *p*(5981): 1.65E-28, *p*(3718): 3.20E-09, *p*(3494): 4.74E-24, *p*(2383): 5.39E-14, *p*(5624): 4.32E-03, *p*(1892): 1.00E + 00, *p*(1304): 1.00E + 00, *p*(3109): 1.00E + 00. (**E**) CD20-specific cytotoxicity, computed as AUC (Raji − K562) across three E:T ratios (Fig. EV5). Bars show mean ± SE; BH-corrected one-sided *z*-test vs. negative control. Significance stars relative to negative controls: *p < 0.05; **p < 0.01; ***p < 0.001; *n* = 3 technical replicates. Exact BH-corrected *p* values are as follows: *p*(–Control): 1.00E + 00, *p*(+Control): 2.58E-29, *p*(1506): 3.71E-19, *p*(5300): 7.04E-02, *p*(5981): 6.79E-01, *p*(3718): 1.25E-04, *p*(3494): 1.39E-03, *p*(2383): 2.72E-20, *p*(5624): 1.00E + 00, *p*(1892): 1.00E + 00, *p*(1304): 1.00E + 00, *p*(3109): 1.00E + 00. (**F**) SPR measurements for design 2383 indicate moderate binding affinity to detergent-solubilized CD20 when expressed as an isolated 80-mer (*K*_D_ = 643 nM). (**G**) Boltz-1 structures of selected CD20-bound designs that broadly outperformed the negative control. Panel (**A**) was created using BioRender (https://biorender.com). Source data are available online for this figure.

Each of the top ten binders were cloned as individual CAR constructs in a manner identical to how they would have appeared in the pooled library. CAR-T cells expressing each of these binders were characterized in a series of assays probing different aspects of T cell activation and effector function, including assays for expression, proliferation, expansion, cytokine production, and target cell lysis (Fig. 3A). CD20-targeting, scFv-based CAR-T (Press et al, 1987; O’connor et al, 2013; Panjwani et al, 2016) and untransfected TCR + T cells served as positive and negative controls, respectively.

**Figure EV1:**
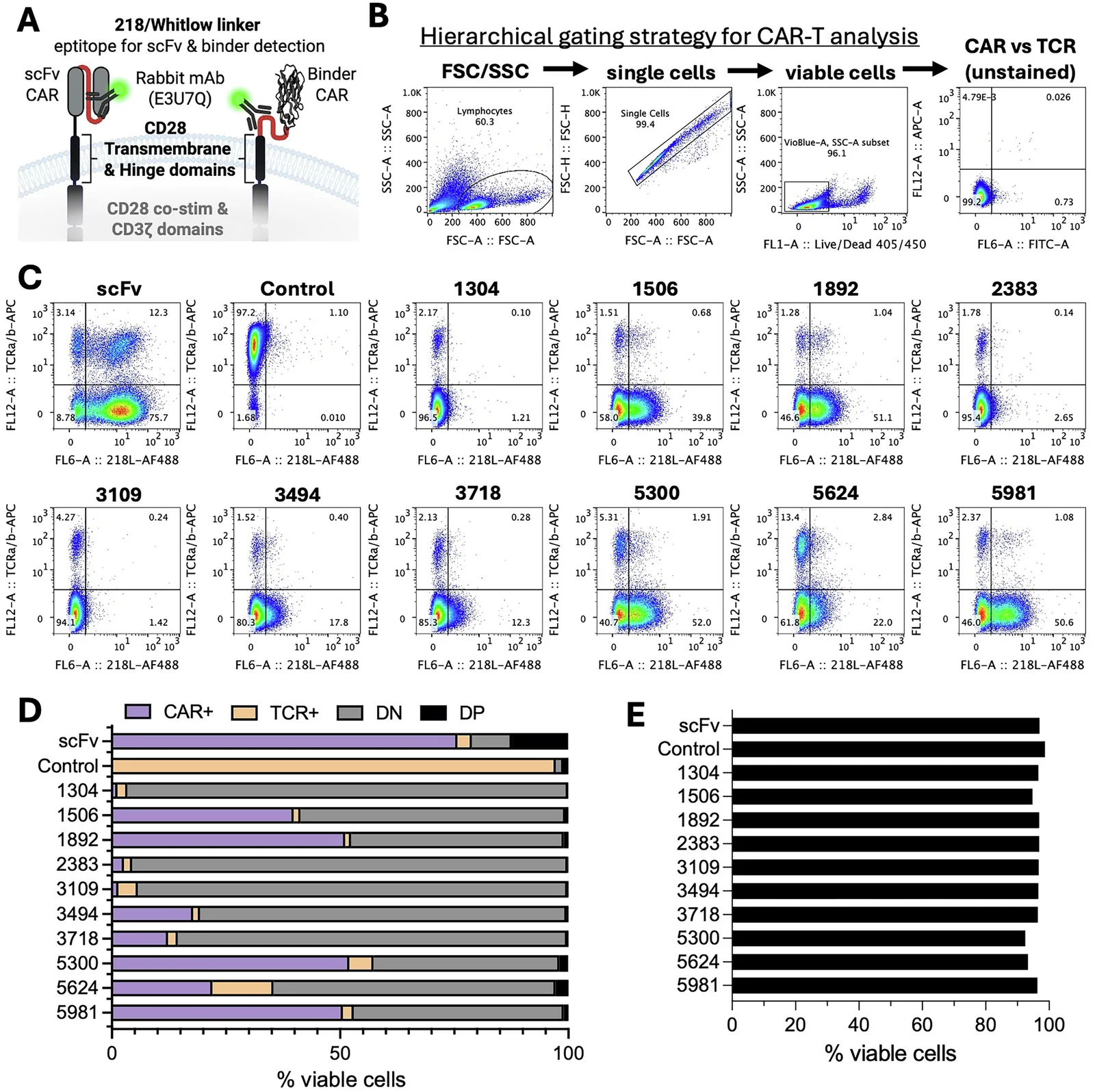
CAR and TCR expression determined by flow cytometry. (**A**) 218/Whitlow linker present in the scFv was designed into the binder construct as an antibody epitope. (**B**) Hierarchical gating strategy for viable CAR-T enumeration, as shown in (**C**) scatter plots depicting CAR and TCR surface expression for controls and binder variant CAR-T cells. (**D**) graphical representation of flow cytometry data showing T cell populations expressing CAR, TCR, neither, or both. (**E**) Viability assessment of controls and binder variant CAR-T cells. Source data are available online for this figure.

Flow cytometry was used to immunophenotype TCR and CAR expression (Fig. EV1A,B). Seven of the designs (1506, 1892, 3494, 3718, 5300, 5624, and 5981) proved to be CAR+ cells, with 10–50% of the population being CAR+ cells (Fig. EV1D). Design 2383 had <5% CAR+ cells, while 1304 and 3109 all but lacked expression (Fig. EV1D). 1304 and 3109 were kept for the remaining functional assays to serve as internal negative controls.

**Figure EV2:**
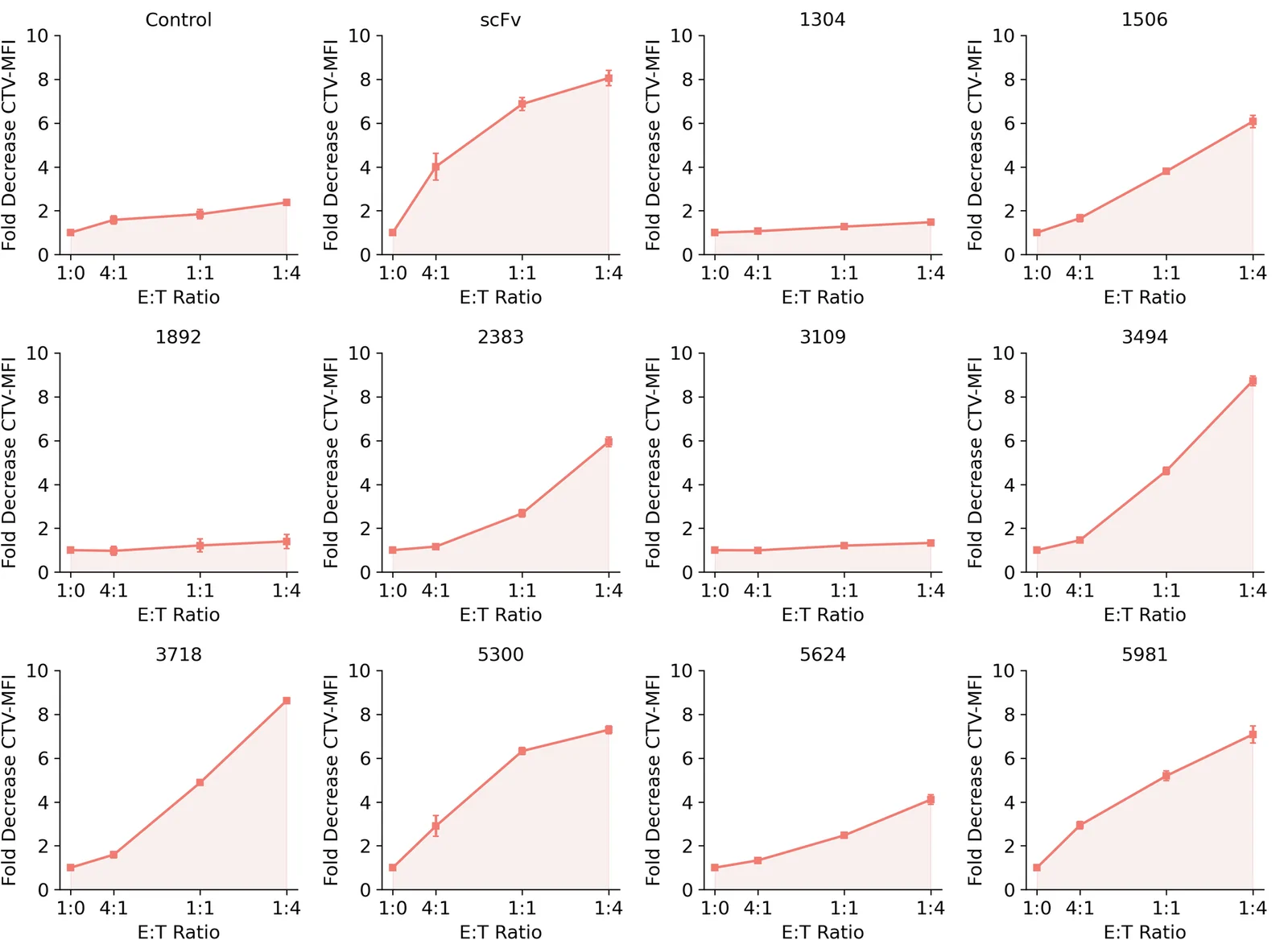
Proliferation via a decrease in CTV-MFI. Proliferation was measured over 5 days following stimulation with Raji (CD20+) targets across multiple E:T ratios by decrease in median fluorescence intensity (MFI) of the cell trace violet (CTV) signal. Data represent three technical replicates from one donor; bars indicate standard error centered on the mean. Source data are available online for this figure.

**Figure EV3:**
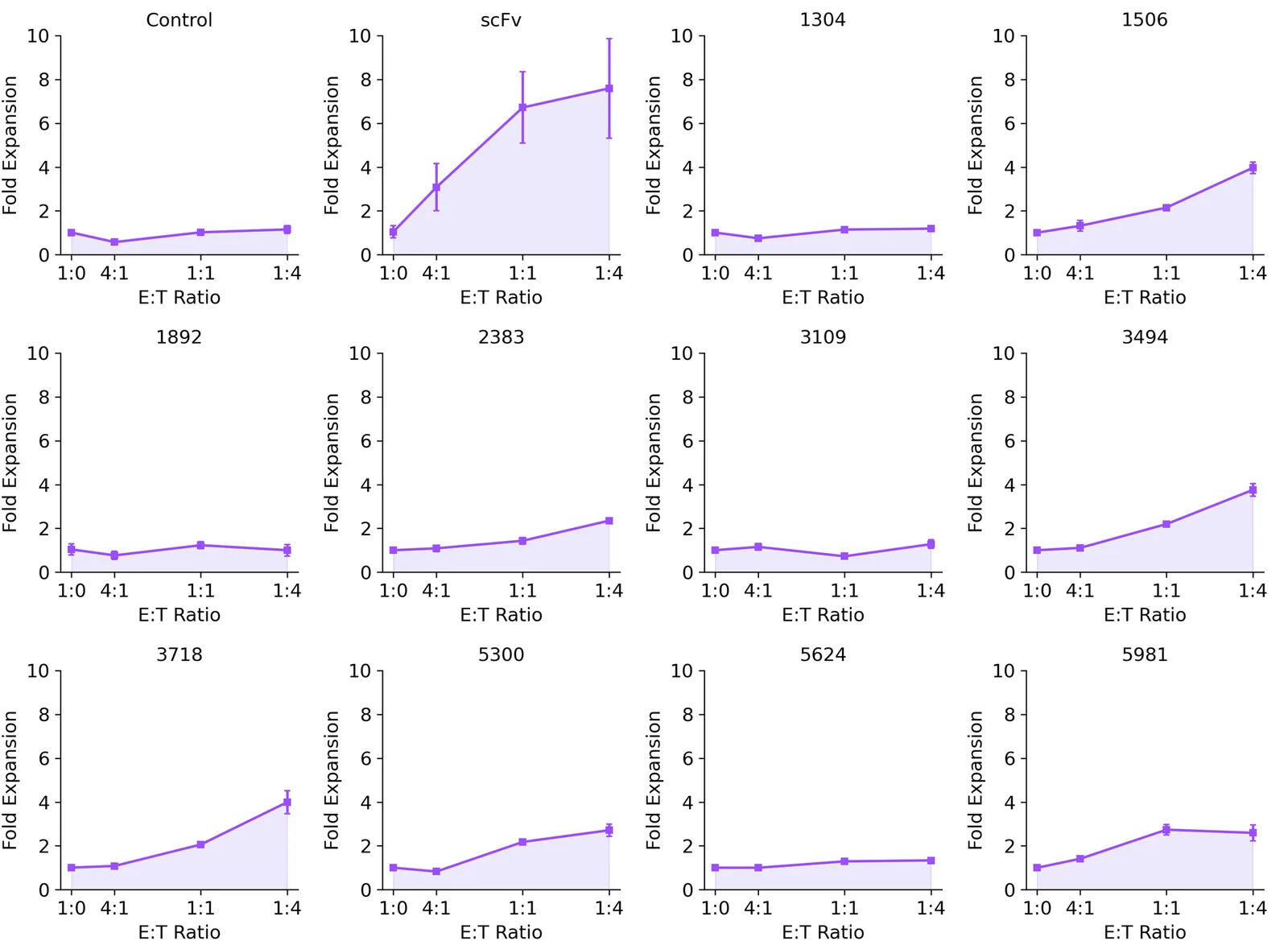
Fold expansion by cell counts. Total CAR-T expansion was measured over 5 days following stimulation with Raji (CD20+) targets across multiple E:T ratios by total viable CAR-T yield using volumetric flow cytometry. Data represent three technical replicates from one donor; bars indicate standard error centered on the mean. Source data are available online for this figure.

CAR-T cell cumulative expansion and cell division were evaluated five days after stimulation with CD20-positive target cells at varying effector-to-target ratios. Seven clones outperformed the CAR-negative untransfected control T cells in expansion and proliferation (Figs. 3B,C and EV2 and EV3; Appendix Fig. S3; clones 1506, 2383, 3494, 3718, 5300, 5624, and 5981). The remaining three designs (1304, 1892, and 3109) underperformed or were equal to the negative control. These results suggest that the hit rate of 707/12,000 in the pooled proliferation assay is likely an upper bound.

**Figure EV4:**
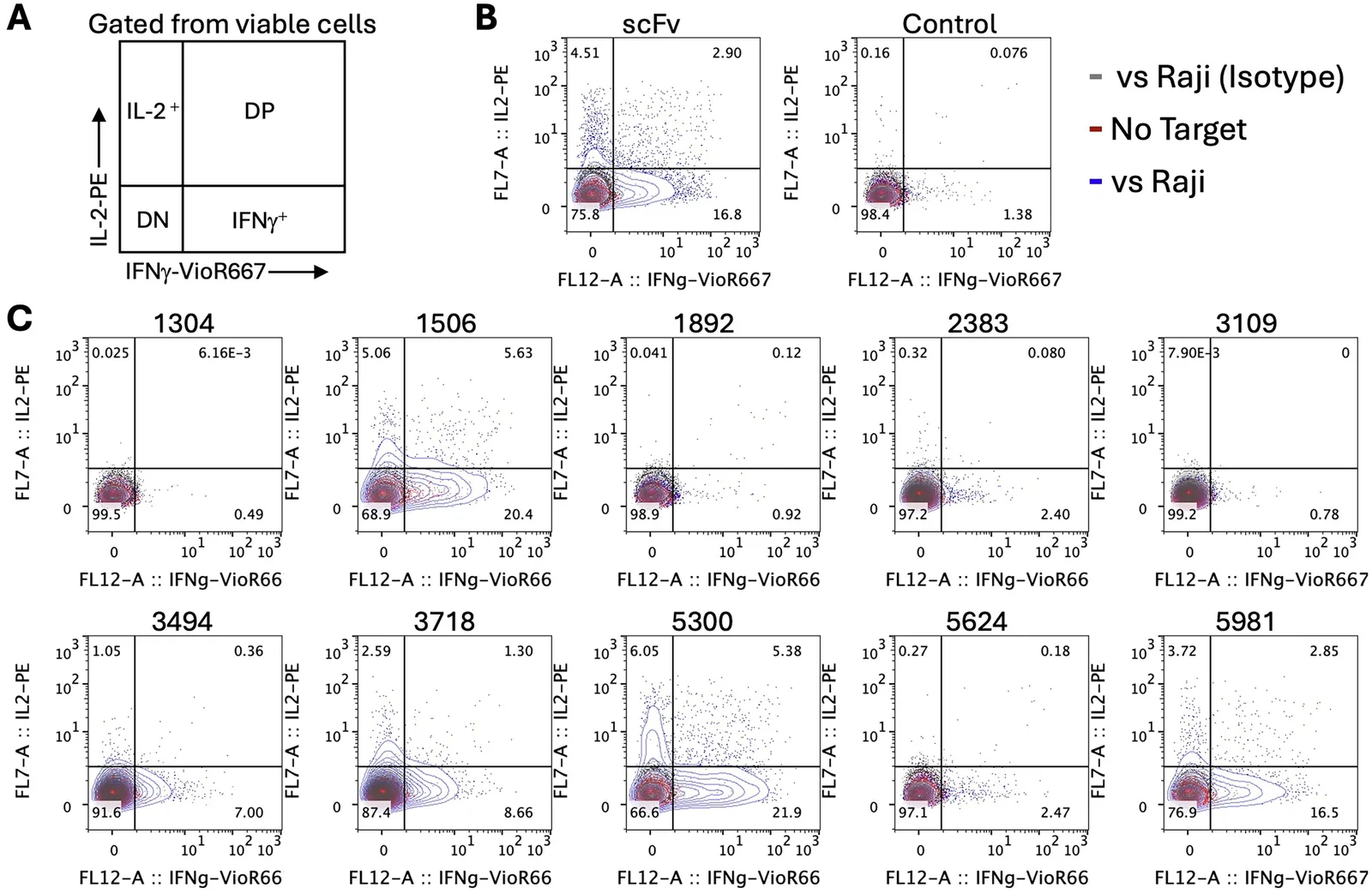
CAR-T cell cytokine generation induced by CD20+ Raji target cells. (**A**) Gating strategy illustrating cytokine-generating populations as analyzed by flow cytometry. (**B**) CD20 scFv CAR and Control (unedited, TCR + CAR-neg T cells), were stained with fluorochrome-conjugated antibodies after incubating alone (red) or in co-culture with Raji target cells. Specifically, anti-IFNg and anti-IL2 antibodies, or the requisite isotype antibody controls, were used to analyze both controls and (**C**) individual CD20 binders as CAR-T cells. Source data are available online for this figure.

Target-specific production of the cytokines IL2 and IFNγ was assessed with flow cytometry, and the same seven designs outperformed the negative control when cultured with CD20+ targets (Figs. 3D and EV4). Clones 1506, 5300, and 5981 showed the most activity, generating similar, if not more, cytokines than the scFv control. The remaining three designs (1304, 1892, and 3109) once again did not outperform the negative control.

**Figure EV5:**
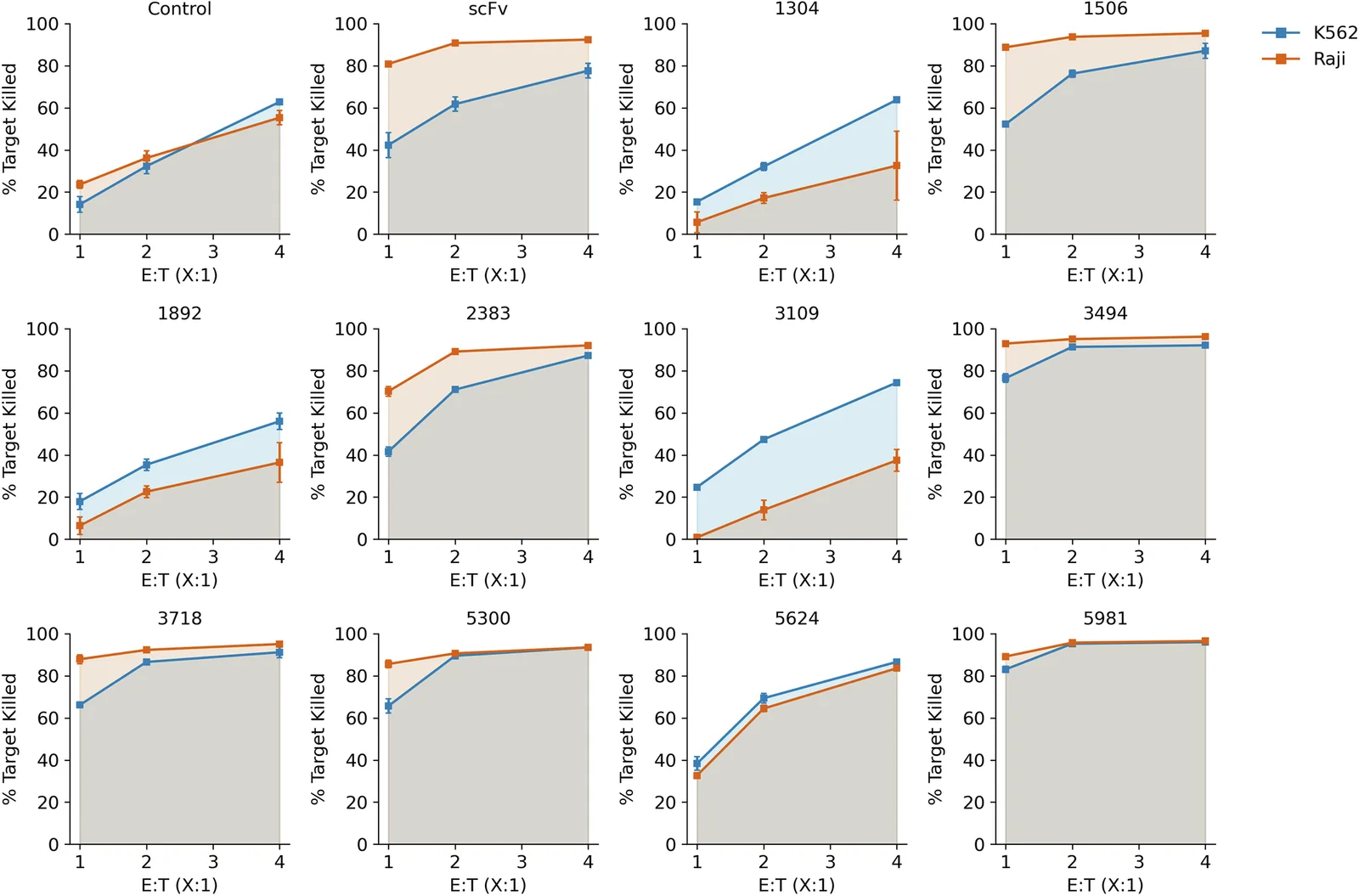
Raji-specific cytotoxicity vs K562 control. GFP assay readouts across multiple E:T ratios for Raji (CD20+) and K562 (CD20−) targets, overlaid for direct comparison. Data represents three technical replicates from one donor, bars indicate standard error centered on the mean. Source data are available online for this figure.

Finally, specific lysis of CD20+ target cells over 48 h was evaluated by co-incubating CAR-T cells with CD20-positive target cells (Raji-GFP) and CD20-negative controls (K562-GFP) at varying effector-to-target ratios. Although the same seven designs outperformed the negative control in terms of non-specific cytotoxicity, lysing both CD20+ and CD20- target cell lines at the measured E:T ratios, only four of these (1506, 2383, 3494, and 3718) had significant, specific lysis of CD20+ cell lines above the negative control (Figs. 3E and EV5). Two of these (1506 and 2383) had comparable specificity and non-specific K562 killing to the scFv positive control (Fig. EV5). The results of the individual functional assays are summarized in Table EV1.

### Characterization of binding

Activation in the presence of CD20+ Raji cells implies that there were productive interactions with the designed binders, so we further assessed binding via a surface plasmon resonance assay in which detergent-solubilized human CD20 was flowed across the immobilized 80-amino-acid designs. The known scFv was used as a positive control and was found to have a *K*_*D*_ of 1.75 nM, confirming that the assay preserves at least the epitope engaged by the scFv (Appendix Fig. S4). Of the top ten binders, only three were found to have appreciable binding in this assay: 2383 had a *K*_*D*_ of 643 nM and designs 1506 and 5981 had detectable binding but weak signal, estimated by Anabel (Krämer et al, 2019) to be between 200–600 nM (Fig. 3F; Appendix Fig. S4). This weak signal is perhaps unsurprising, since optimal CAR function often occurs within a moderate range of binding constants; if binding is too high, trogocytosis and T cell exhaustion can occur, and many lower-affinity CARs have been found to elicit good if not better tumor elimination (Olson et al, 2022; Mao et al, 2022; Ghorashian et al, 2019). However, there is no universal optimal affinity range, as CAR activity also depends on antigen density, epitope location, receptor architecture, and assay conditions. Our results are consistent with functional activity from moderate- or low-affinity binders, but the limited number of designs with quantifiable binding does not establish that successful CD20 CARs universally require low-affinity binding.

One caveat to these results is that the binding assay tested the binders as isolated 80-mers rather than in the context of the CAR scaffold, and thus proper folding, display, and avidity effects may have failed to occur. Secondarily, these binders were created using prokaryotic cell-free protein synthesis, which may have contributed to differences in folding efficiency, disulfide formation (design 5838, for example, is predicted to form 3-4 disulfide bonds), and post-translational processing between a prokaryotic lysate and the mammalian secretory pathway. As such, failure to bind in this assay may instead reflect differences in binder structural context and expression rather than design quality (Thomas et al, 2023).

### Competition winners and outcomes of methodological choices

Overall, design 2383 from Nucleate UK, London bound strongly to CD20, had high specific lysis, moderate proliferation and expansion, low cytokine %, and low CAR+ % (Table EV1). Design 1506 from Perez Lab Gators had weaker binding but high specific lysis, cytokine %, proliferation, expansion, and CAR + %. Design 5981 from the Schoeder Lab also had weaker binding and high proliferation, expansion, and CAR + %, but had strong lysis with weak specificity. Designs 3494, 3718, 5300, and 5624 (from SNU LCDD, SNU LCDD, amigo acids, and binding illini, respectively) had no measured binding despite being broadly functional, with two of the four having target-specific lysis, and all four having overall lysis, suggesting that these designs may have largely relied on avidity effects or mechanisms other than CD20 recognition. Designs 1304 and 3109 did not express, while design 1892 expressed but failed all functional assays. One conclusion is thus that seven of the top ten designs were successful in functional assays (Fig. 3G), suggesting that a variety of generative design approaches (from teams Nucleate UK London, Perez Lab Gators, Amigo Acids, Schoeder Lab, SNU LCDD, and Binding Illini) can yield functional CAR-T cells (Appendix Fig. S25).

**Figure 4:**
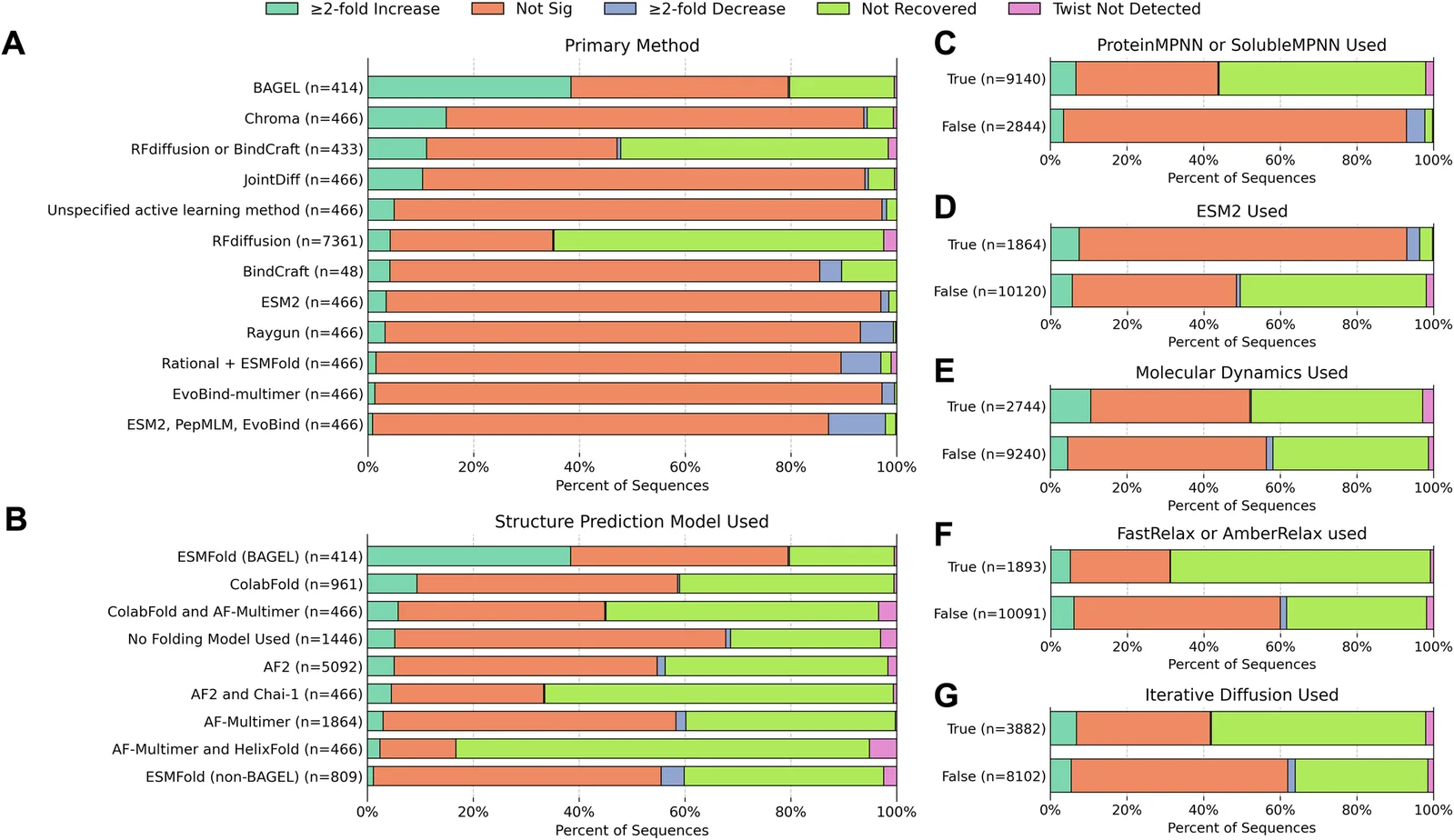
Experimental outcomes based on methodological choices. Methodological choices were categorized into: (**A**) choice of primary generative method, (**B**) choice of structure prediction model, (**C**) the use of ProteinMPNN or SolubleMPNN, (**D**) the use of ESM-2, (**E**) the use of molecular dynamics prior to or after generation, (**F**) the use of FastRelax or AmberRelax prior to or after generation, and (**G**) the use of diffusion models in an iterative manner. Although some trends are visible, the causality is unclear and may result from artifacts of this categorization failing to capture higher-order methodological details. Source data are available online for this figure.

**Figure 5:**
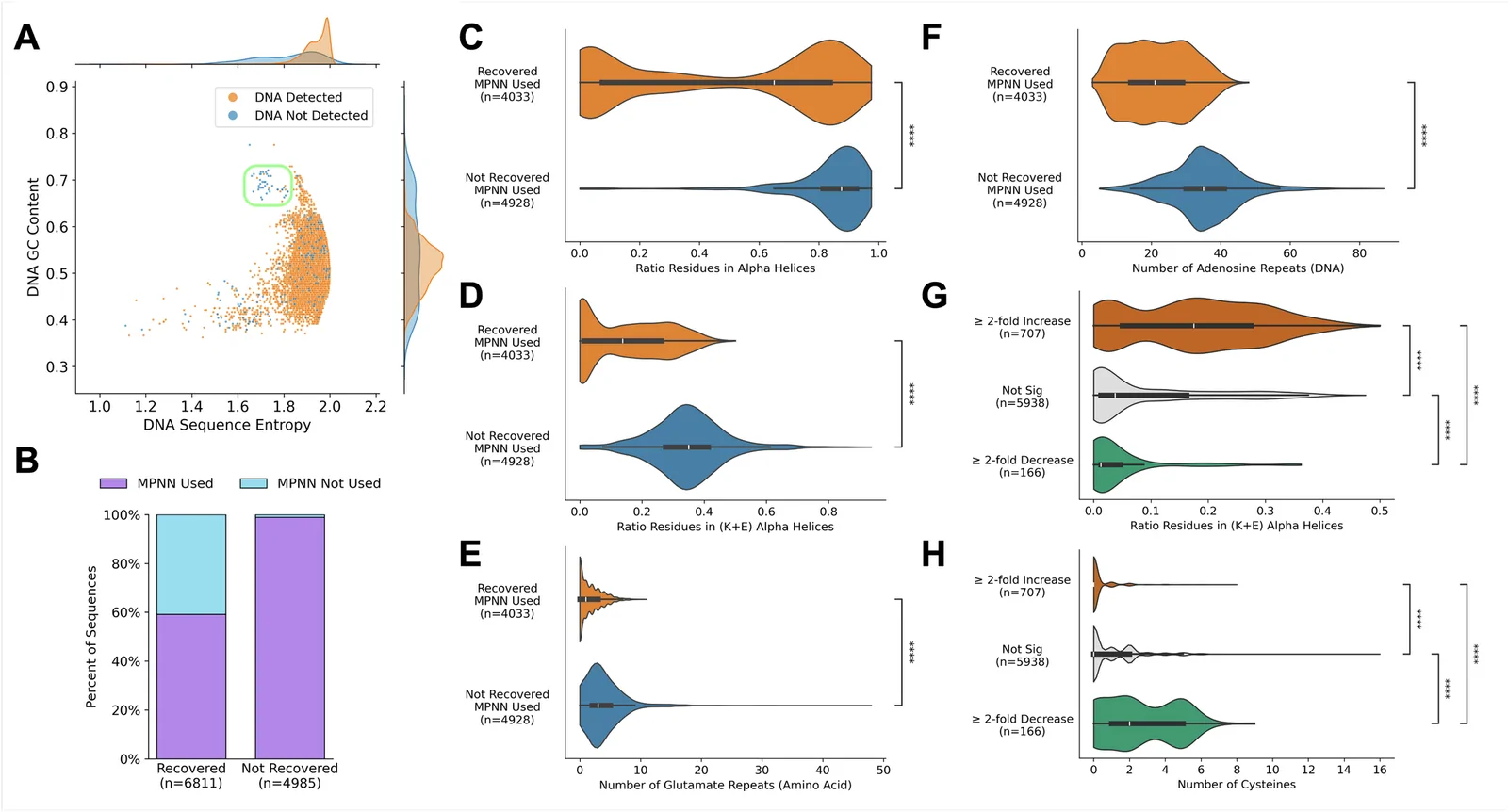
Computational analysis suggests mechanisms leading to success and failure. (**A**) The combination of DNA GC content and Shannon entropy separates out 55 designs that failed to synthesize as DNA. (**B**) Almost all of the designs that failed to be recovered were created with ProteinMPNN or SolubleMPNN. (**C**,** D**) The MPNN designs that failed to be recovered were enriched in alpha helices that contained mostly lysine and glutamate. (**E**,** F**) These designs had significant glutamate repeats (EE) in the amino acid sequence, and adenosine repeats (AA) in the DNA sequence, both of which have been shown to cause ribosomal termination, suggesting a possible mechanism for the absence of these sequences. (**G**) The designs that caused a ≥2-fold increase in CD20-specific proliferation were enriched in (K + E) alpha helices, though to a lesser extent than those that were not recovered. (**H**) The designs that caused a ≥2-fold decrease in CD20-specific proliferation were enriched in cysteines predicted to form disulfide bonds within the binder chain (Appendix Fig. S8). Significance from two-sided BH-corrected Mann–Whitney–Wilcoxon tests (**** for *p* ≤ 0.0001, exact *p* values as follows: Panel (**C**): 4.58E-496; Panel (**D**): 3.67E-742; Panel (**E**): 7.54E-381; Panel (**F**): 2.33E-657; Panel (**G**): increase vs not sig: 2.49E-66, increase vs decrease: 3.61E-40, not sig vs decrease: 8.38E-09, Panel (**H**): increase vs not sig: 1.92E-54, increase vs decrease: 3.35E-76, not sig vs decrease: 1.09E-30); group sizes reported in subpanels. For the boxplots within the violins, the white dot represents the median (50th percentile), the box bounds represent the inner quartile range (IQR; 25th to 75th percentiles), and the thin line represents whiskers extending to the most extreme observations lying within 1.5 x IQR. Outliers are not shown in the box plot. Source data are available online for this figure.

The choices made by each team in the design process were numerous and provide ample ground for further methodological analysis. A summary of the predominant methodologies and experimental outcomes for all 28 teams can be found in Table 2. The generative methods associated with high performance were BAGEL (Lála et al, 2025) and Chroma, followed by RFdiffusion, BindCraft, and JointDiff (Zhu et al, 2025) (Fig. 4A). Many designs were verified or generated with structure prediction models, with BAGEL’s use of ESMFold in first, then ColabFold and AlphaFold-like models, and non-BAGEL ESMFold in last (Fig. 4B). ESMFold was used to generate designs in BAGEL, whereas it and the other structure prediction models were used largely for verification by the other teams. As such, the causal impact of the structure prediction models is unclear. That said, the effect of ProteinMPNN or SolubleMPNN (MPNN) usage is very strong, with the use of these models causing an increase in CD20-specific enrichment (6.6% ≥2-fold increase with MPNN vs 3.4% without; χ² test *p* < 1e-4) and simultaneous lack of detection in the proliferation assay (53.9% not recovered with MPNN vs 2.0% without; χ² test *p* < 1e-4), as further analyzed below (Figs. 4C and 5B). Conversely, ESM-2 usage resulted mostly in sequences that were recovered in the proliferation assay (3.3% not recovered with ESM-2 vs 48.6% without; χ² test *p* < 1e-4) (Fig. 4D). Using molecular dynamics tools on the structure either before generating to obtain an optimal pose, or afterwards for filtering, correlated with a modest increase in successful designs (10.5% ≥2-fold increase with MD vs 4.5% without; χ² test *p* < 1e-4), though this was not true for FastRelax or AmberRelax (5.1% ≥2-fold increase with Amber/FastRelax vs 6.0% without; χ² test *p* = 0.117) (Fig. 4E,F).

**Table 2:** Summary of team methods and outcomes.

Team	Primary generative method	Sequence design method	Designs tested, *n*	DNA detected, *n* (%)	Recovered, *n* (%)	≥2-fold proliferation, *n* (%)
AIBI*	JointDiff	JointDiff	466	464 (99.6%)	441 (94.6%)	48 (10.3%)
Amigo Acids*	RFdiffusion	ProteinMPNN	187	185 (98.9%)	65 (34.8%)	11 (5.9%)
Antigeniuses	RFdiffusion	ProteinMPNN	466	466 (100%)	450 (96.6%)	13 (2.8%)
Binder Bot Engineers	Active learning	ProteinMPNN	466	466 (100%)	457 (98.1%)	23 (4.9%)
Binder Builder	RFdiffusion	SolubleMPNN	466	465 (99.8%)	110 (23.6%)	13 (2.8%)
Binding Illini*	Chroma, ESM	ProteinMPNN	466	463 (99.4%)	440 (94.4%)	69 (14.8%)
Bionova	RFdiffusion	ProteinMPNN	466	463 (99.4%)	156 (33.5%)	21 (4.5%)
Bryant Lab	EvoBind-multimer	EvoBind-multimer	466	466 (100%)	464 (99.6%)	6 (1.3%)
CAD Pro*	ESM-2, PepMLM, EvoBind	ESM-2, PepMLM, EvoBind	466	465 (99.8%)	456 (97.9%)	4 (0.9%)
CIIRC	RFdiffusion	ProteinMPNN	466	442 (94.9%)	78 (16.7%)	11 (2.4%)
D12	RFdiffusion	ProteinMPNN	466	462 (99.1%)	95 (20.4%)	17 (3.6%)
Evaneil	RFdiffusion	ProteinMPNN	466	424 (91.0%)	49 (10.5%)	9 (1.9%)
Foldsmiths	RFdiffusion	ProteinMPNN	465	458 (98.5%)	101 (21.7%)	18 (3.9%)
Furman Lab	RFdiffusion, BindCraft	ProteinMPNN	433	426 (98.4%)	207 (47.8%)	48 (11.1%)
LBM*	RFdiffusion	ProteinMPNN, CARBonAra	466	450 (96.6%)	211 (45.3%)	27 (5.8%)
Molecule Masters	RFdiffusion	ProteinMPNN	466	460 (98.7%)	122 (26.2%)	16 (3.4%)
N6 (Team Anonymous)	Rational design	Rational design	466	461 (98.9%)	454 (97.4%)	7 (1.5%)
Nucleate UK London*	BAGEL	BAGEL, SolubleMPNN	414	412 (99.5%)	330 (79.7%)	159 (38.4%)
Perez Lab Gators*	RFdiffusion	ProteinMPNN	466	461 (98.9%)	186 (39.9%)	38 (8.2%)
Perforators	RFdiffusion	ProteinMPNN	343	328 (95.6%)	32 (9.3%)	2 (0.6%)
Picaso Protein	Raygun	Rules-based mutation	466	465 (99.8%)	463 (99.4%)	15 (3.2%)
Schoeder Lab*	RFdiffusion	ProteinMPNN	466	419 (89.9%)	236 (50.6%)	28 (6.0%)
SNU LCDD*	RFdiffusion	ProteinMPNN	466	463 (99.4%)	358 (76.8%)	36 (7.7%)
The Physicist	Rational design	Rational design	466	466 (100%)	459 (98.5%)	16 (3.4%)
Tumor Inhibitors	RFdiffusion	ProteinMPNN	466	461 (98.9%)	181 (38.8%)	23 (4.9%)
Undergraduate Design	RFdiffusion	ProteinMPNN	466	463 (99.4%)	98 (21.0%)	14 (3.0%)
Virtue Therapeutics	BindCraft	BindCraft	48	48 (100%)	43 (89.6%)	2 (4.2%)
Zist Rayanesh	RFdiffusion	ProteinMPNN	308	308 (100%)	62 (20.1%)	10 (3.2%)

Methods indicate the primary generative and sequence design approaches reported by each team; secondary generative strategies, templates, structural refinement, filtering, and ranking methods are detailed in Appendix Information E. An asterisk after a team name indicates that at least one design was selected for individual functional validation.

During the solicitation, teams were asked to submit their sequences in the rank order that they deemed would produce successful CARs, in which teams ranked by perplexity, folding scores, and other combined metrics. Rank ordering was significantly positively correlated with recovery for Binder Bot Engineers (ρ = 0.20), Molecule Masters (ρ = 0.17), and CAD Pro (ρ = 0.14), and negatively correlated for Perez Lab Gators (ρ = −0.11), SNU LCDD (ρ = −0.12), Tumor Inhibitors (ρ = −0.12), Binding Illini (ρ = −0.14), Zist Rayanesh (ρ = −0.22), Evaneil (ρ = −0.41), and Foldsmiths (ρ = −0.51) (Appendix Fig. S5). Rank ordering was not significantly positively correlated with successful proliferation for any teams, but it was significantly negatively correlated with successful proliferation for Binder Bot Engineers (ρ = −0.25) and Foldsmiths (ρ = −0.32) (Appendix Fig. S6). The strong anticorrelations found in several teams, in which the lower-ranked designs were more successful, may be indicative of optimal CAR binding being of moderate affinity, and further suggests that optimizing with structure-based metrics is not always optimal.

There were common methodologies shared between the top-performing teams (Perez Lab Gators, Amigo Acids, Schoeder Lab, Nucleate UK London, SNU LCDD, Binding Illini, and Furman Lab). First, all of these teams used a diffusion model, either RFdiffusion or Chroma. Second, all of these teams used ProteinMPNN or SolubleMPNN for some stage of the design. Third, many of these teams employed these models in an iterative or refinement strategy, which was found to slightly improve success, though this effect was less significant than the other findings (6.8% ≥2-fold increase with iterative diffusion vs 5.4% without; χ² test *p* = 0.0033) (Fig. 4G). The teams with the two best-performing binders (Perez Lab Gators, Amigo Acids, Appendix Fig. S7) both had highly similar pipelines employing iterative RFdiffusion, ProteinMPNN and FastRelax. Notably, the team with the top-performing design (Perez Lab Gators, Appendix Fig. S7) was the only team that reported folding their designs within the greater CAR sequence. As doing this is computationally trivial and well-motivated by first principles, we recommend folding designs within the full CAR sequence for future efforts.

### Exploratory analysis of factors leading to design failure and success

The diversity of sequences generated during the competition by various methods are themselves a wealth of information on the success of AI-driven tools, as applied by many different users, for designing proteins to modify cellular functionality. The nucleotide and amino acid sequences of the 12,000 designed binders were used to compute over 400 metrics (Appendix Tables S1–S7), ranging from simple readouts like sequence entropy to more involved ones such as the average energy of the binder–CD20 interface on a predicted structure (Methods; Appendix Information B). The varied features were further analyzed for their ability to predict (a) detection in the DNA quality report, (b) whether or not the design was recovered by NGS after proliferation, and (c) the CD20-specific change in proliferation.

The Twist plasmid pool quality report indicated that 204 of the 12,000 designs were not detected. Almost all of the failed designs (71%) originated from 5 of the 28 teams (Fig. 2C), suggesting that, despite codon optimization, particular design features led to synthesis failures. One common cause seemed to be high GC content and repetitive sequences; a cluster of failed sequences with high GC content and moderately low Shannon entropy can be observed (Fig. 5A). Applying a more stringent filter to remove sequences with <1.85 Shannon entropy and ≥0.65 GC content would have removed a quarter of the DNA failures (55 designs), while having no impact on functional sequences.

Of the synthesized designs, 6811 were recovered by NGS after the proliferation assay (≥25 reads in all six replicates), and 4985 were not recovered. As a functional CAR is necessary for sustained T cell fitness and survival, failure in this readout suggests failed protein expression, solubility, or folding. The recovered and non-recovered designs differ markedly in their amino acid compositions, with the non-recovered sequences being preferentially enriched in glutamate, lysine, and alanine (Appendix Fig. S8).

Strikingly, 98.9% of the non-recovered sequences were designed with ProteinMPNN or SolubleMPNN (MPNN), whereas the recovered sequences came from both these models (59.2%) and other methods (40.8%) (Fig. 5B). To determine why some MPNN designs failed where others succeeded, the designs created without MPNN were removed, resulting in 4033 recovered and 4928 non-recovered sequences. Categorizing the secondary structure of these designs’ Boltz-1 predicted structures indicated that most of the non-recovered MPNN sequences were almost entirely alpha helices, with an average of 83% of the residues being part of a helix (Fig. 5C). Compared to the alpha helices from the recovered sequences, those from the non-recovered sequences were highly enriched in lysine (K) and glutamate (E) (Fig. 5D). ProteinMPNN has been found to preferentially choose K and E when using the default sampling temperature of T = 0.1, an effect which is not present at higher temperatures of T = 1.0, as shown in Fig. S6 of the original ProteinMPNN paper (Dauparas et al, 2022; Ertelt et al, 2024). It has also been found that RFdiffusion commonly produces alpha helices, which may compound the choice of lysine (Sappington et al, 2026; Nick Pace and Martin Scholtz, 1998). In fact, the ratio of residues in K + E alpha helices was by far the strongest predictor of sequence recovery, averaging 35% of residues for non-recovered MPNN and 15% for recovered MPNN and achieving 0.91 ROC-AUC using a logistic regression model trained with stratified fivefold cross-validation (Table EV2, Appendix Fig. S9, Methods). A subsequent study independently confirmed that K + E content is strongly associated with recovery failure in a phage display system (Didi et al, 2026), supporting the generality of this signal beyond CAR-T.

We thus formulated working hypotheses to explain the mechanism behind the importance of K + E alpha helices. Repeats of acidic amino acids are known to directly impact protein synthesis since “nascent chains enriched in aspartic acid (D) or glutamic acid (E) in their N-terminal regions alter canonical ribosome dynamics, stochastically aborting translation” (Ito et al, 2022). There were 3.9 EE repeats in the non-recovered MPNN sequences on average versus 1.6 in the recovered, and this feature achieves 0.82 ROC-AUC (Fig. 5E). Similarly, adenosine repeats in the nucleotide sequence have been shown to be potent translational disrupters (Arthur and Djuranovic, 2018; Tesina et al, 2020), which may correlate with the use of lysine (AAA, AAG) and glutamate (GAA) codons. The non-recovered sequences were significantly enriched in adenosine repeats in the DNA sequence, with means of 35.4 adenosine dinucleotide repeats in the non-recovered versus 21.7 in the recovered (Fig. 5F). The number of AA repeats was one of the most predictive features for sequence recovery, achieving 0.88 ROC-AUC, and also plausibly explaining why DNA sequence entropy alone had high predictive power at 0.90 ROC-AUC (Table EV2). One alternative explanation for the K + E trend is that high enrichment causes poor folding or rapid degradation by a post-translational mechanism (Das and Pappu, 2013). That said, sequences that had ≥2-fold increase in proliferation still had a mean of 18% of residues in K + E alpha helices (relative to the 35% seen in non-recovered sequences and to the 4% seen in the designs without significant change in proliferation (Fig. 5G)), suggesting that low-temperature ProteinMPNN sampling may be desirable up to a limit, after which adverse effects take place.

The sequences that exhibited CD20-specific depletion contained a large number of cysteines that were predicted to form disulfides within the binder chain (Fig. 5H; Appendix Fig. S8). Given that tonic signaling can be enhanced through disulfide bond formation between hinge domain sulfhydryls (Lazar-Molnar et al, 2006; Long et al, 2015; Folimonova et al, 2024), and that inefficient folding (or, more likely, misfolding) may expose the hinge sulfhydryls or those in the binding domain, weak tonic signals might be greatly enhanced upon CD20 contact and subsequent disulfide bond formation.

Beyond these individual correlations, global confidence statistics from structure prediction models, such as ipTM and pLDDT, did not predict experimental outcomes (Table EV2). Likewise, ipSAE (Dunbrack Jr, 2025), LIS score (Kim et al, 2024), SAP score (Chennamsetty et al, 2010), Rosetta InterfaceAnalyzer metrics (Stranges and Kuhlman, 2013), PDockQ (Bryant et al, 2022; Zhu et al, 2023), ProteinMPNN likelihoods, and ESM-2 pseudo-log likelihood (PLL) also lacked substantial predictive power, which, for the structure-derived metrics, may be due to the additional layer of noise from using predicted structures (Table EV2). Additional features and their mutual information are shown in Appendix Figs. S10–S12.

The two likelihood-based metrics, ProteinMPNN likelihood and ESM-2 pseudo-log likelihood (PLL), were additionally affected by artifacts in the data, leading to deviation from expected scoring behavior. Likelihoods obtained from ProteinMPNN and SolubleMPNN on the Boltz-1-predicted structures were generally biased in favor of designs created using these models (Appendix Fig. S13). ESM-2 PLL correlated with and slightly underperformed in predictive power (Table EV2), amino acid sequence entropy for most sequences (Appendix Fig. S14). This trend can be explained by the masked language modeling task becoming easier the less entropic the sequence is, which is further exemplified by the two outlier clusters defying this trend, which contained large, repeated regions that plausibly make the task of single residue infilling trivially easy with an in-context lookup, in agreement with existing literature (Kantroo et al, 2025).

These findings exemplify how simple metrics can be more predictive of function than structure-based or layered deep learning-based scores. To explore their practical efficacy, several retrospective filters were applied to the 12,000-sequence dataset (Appendix Figs. S15 and S16; Tables EV3 and EV4). Removing designs with ≥60% GC content (Appendix Fig. S17) and <1.9 DNA sequence entropy (Appendix Fig. S18), ≥43 AA repeats in the DNA (Appendix Fig. S19), ≥8 EE repeats in the amino acid sequence (Appendix Fig. S20), and ≥30% of residues in a K + E alpha helix (Appendix Fig. S21) results removes 4600 sequences and increases the percent recovered after proliferation from 56.8 to 81.2% while increasing the percent of those that had CD20-specific proliferation from 5.9 to 7.6%. Further removing any sequences with cysteines (Appendix Fig. S22) raises the CD20-specific proliferation rate to 10.6%, though it reduces the percent recovered slightly to 72.6%. Notably, this filter removes two of the designs from the top 10 that lacked function entirely (1304 and 1892) while preserving the rest. In contrast, filtering out sequences with <0.5 Boltz-1 ipTM, chosen as an extreme lower bound, would have removed two of the top ten designs that were broadly functional and had binding affinity (designs 1506 and 5981) while only increasing the recovery rate by 0.7% and the CD20-specific proliferation rate by 0.3%. While cysteine content may be CAR-specific, the other features of GC content, sequence entropy, and K + E alpha helix ratio, may reasonably generalize to other protein design applications, both by AI-driven methods and otherwise.

## Discussion

The *Bits to Binders* competition was hosted to evaluate AI-driven protein design tools via a discovery-to-therapeutic pipeline. We collected 12,000 binder designs from 28 teams around the world that were created with a wide variety of approaches. Only 204 variants could not be cloned, in part due to repetitive sequences with high GC content, and the rest were tested in LEAH Labs’ high-throughput screen to evaluate a variety of phenotypes, starting with CD20-specific CAR-T proliferation. 4,985 of the designs (42%) dropped out of the population, indicating that some sequences or proteins interfered with cell viability. To verify that CD20-specific proliferation in the pooled growth assay translated to overall T cell function, we performed a series of functional assays on the ten best-performing unique designs, measuring specific cytotoxicity, cytokine release, proliferation, and expansion. We found that the majority of binders (7/10) outperformed the negative control across CD20-specific cytokine release, proliferation, and expansion, and 4/10 outperformed it on CD20-specific cytotoxicity. Ultimately, all of the designs underperformed the scFv positive control in all functional readouts except for cytokine production, in which design 5300 achieved more than 50% higher intracellular accumulation when cultured with CD20+ targets. One explanation for the general underperformance of the designs compared to the scFv is that the designs were only 80-amino-acid long compared to the 250-amino-acid scFv.

Almost all of the designs that failed to be detected in the proliferation screen (98.9%) were created with ProteinMPNN or SolubleMPNN. We found that most of the failed MPNN designs preferentially contained highly adenosine-rich, lysine- and glutamate-containing alpha-helices, encompassing an average of 35% of residues versus only 15% in the recovered designs. While unnatural structures may pose problems on their own, potentially misfolding or being degraded by post-translational mechanisms, we believe that many of the MPNN designs failed because of translational interference by glutamate and/or lysine (Ito et al, 2022; Arthur and Djuranovic, 2018; Tesina et al, 2020). To the extent that this hypothesis is correct, it provides a cautionary note that beyond the structure-centric task of binding, optimizing a protein’s amino acid sequence, and corresponding nucleotide sequence, is important for success, especially designing specifically for cellular expression. That said, there was clearly a sweet spot for design, as lower abundance K + E alpha-helices (18%) were moderately predictive of CD20-specific proliferation, in contrast to only 4% K + E in designs that did not show significant changes in proliferation. Designs associated with CD20-specific depletion, potentially indicative of exhaustion (Chen et al, 2023), were highly enriched in cysteines. This validates the cautionary choice many model developers have made in explicitly avoiding cysteines in generated proteins.

Analyzing the top teams, we found some correlations between methodological choices and experimental outcomes. BAGEL, Chroma, Bindcraft, and RFdiffusion all performed moderately well at CAR binder design. However, most of the sequences in this competition were created using RFdiffusion and ProteinMPNN, and this monoculture eventually skewed failure modes (K + E failures). Annealing structures with molecular dynamics prior to or after generation seemed to increase design success, whereas FastRelax and AmberRelax had no effect. While the top-performing teams employed iterative diffusion, this did not increase success across the board. We additionally found little predictive power of common structure-based metrics for the functional outcomes of recovery and proliferation, consistent with the lack of positive correlation between most teams’ submitted rank order and experimental outcomes, suggesting that optimizing solely for structure may not entail functional success.

This study has several important caveats and limitations. First, only a single CAR scaffold was used, and so the performance of these binders in other scaffolds is unclear. Alternative linker, hinge, transmembrane and signaling domains could alter binder orientation and surface expression, as well as antigen sensitivity, tonic signaling and persistence, potentially rescuing inactive designs or diminishing those that performed well in this scaffold (Bernard and Evgin, 2025). Second, sequencing was not done on the pooled populations at an input timepoint, so it is unclear whether initial input abundance impacted subsequent proliferation outcomes. Third, the high-throughput assay tested CD20+ Raji cells versus the absence of Raji cells, not CD20– Raji cells. This means that the interpretation of the specificity is Raji-specific rather than CD20-specific. However, the results from the binding assay indicate that this approximation is mostly valid, given that several bound CD20. This limitation is unlikely to impact many of the takeaways from the exploratory analysis, as those were largely derived from cell viability. We established that some of the designs have broad T cell function, but these assays were only performed in a single clinical isolate. Further, the binding assay used binders outside of their CAR context, made with prokaryotic cell-free protein synthesis, against detergent-solubilized targets. As such, the binding measurements for a given design may not be representative of its cellular context. It was also not established if low CAR + % was due to issues with expression rates or editing efficiency. The baseline non-specific cytotoxicity observed in the individual assays would need to be remediated, alongside other suboptimal properties of the CAR-T cells, before any of these designs could be considered for animal or human testing. It should be stressed that the goal of this competition was not to create therapeutics that are ready for the clinic, but rather to evaluate AI-driven protein design methods in a therapeutic context. The non-specific cytotoxicity is also a potential confounding variable to success in the high-throughput screen and thus adds a layer of noise to the computational analysis. Finally, the scope of the findings is limited both by the sequences coming mostly from RFdiffusion workflows and the within-team sequence redundancy, reducing the effective number of independent observations; in the future, it may prove to be worthwhile to test designs in a more systematic manner or by encouraging a diversity of approaches. Furthermore, the binder designs were limited to 80 amino acids, relative to a typical scFv (230–260 amino acids), which constrains fold diversity and thus the conclusions that can be drawn between generative methods.

Overall, the *Bits to Binders* competition demonstrates that generative protein design has advanced to the point that it can directly be assessed by functional validation. In particular, the identification of potential failure modes (GC dropouts, K + E abundance, excessive cysteines) can further guide CAR-T design, with a set of relatively simple filters almost doubling the rate of CD20-specific proliferating designs. In comparing data from other de novo-designed binder libraries, notably the Adaptyv Bio competition for soluble EGFR engagers, it is evident that biological context is vital to consider for successful protein design (Bozkurt, 2026). Thus, by designing for a functional outcome in primary human T cells instead of evaluating binding affinity alone, the results begin to piece apart the complex interplay between receptor expression, surface display, background (tonic) signaling, ligand-dependence, and induced proliferation. In particular, these results provide a counterpoint to the common perception that robust scFVs are the only viable starting points for CAR-T design and raise the interesting prospect of initially canting design towards lower-affinity spaces. The datasets and principles emerging from efforts like *Bits to Binders* should continue to serve as milestones for standardizing evaluation practices, guiding the development of more biologically informed models, and accelerating the application of AI-driven protein design for functional and ultimately clinical applications.

## Methods

**Table:** Reagents and tools table

Reagent/resource	Reference or source	Identifier or catalog number
**Experimental models**		
Primary human T cells isolated from healthy-donor leukopaks by buoyancy-activated cell sorting (BACS)	Sanguine Biosciences; Akadeum Life Sciences	
Raji-GFP cells (Homo sapiens; CD20-positive target cells)	Walcheck Lab at UMN	CCL-86
K562-GFP cells (Homo sapiens; CD20-negative control target cells)	ATCC	CCL-234, GFP+ made at LEAH
**Recombinant DNA**		
12,000-variant second-generation CAR28z plasmid library (CD8alpha leader-80-aa binder-Whitlow 218 linker-CD28 hinge/transmembrane and co-stimulatory domains-CD3zeta-P2A-DHFR F31S)	This study; oligo-pool cloning by Twist Bioscience; backbone modified from Van Der Stegen et al, 2015	Design sequences/data: https://doi.org/10.5281/zenodo.20451404
Binderless second-generation CAR28z GeneWeld donor plasmid with TRAC homology arms	This study; Webber et al, 2023; Wierson et al, 2020	Synthesized the CAR and cloned into a legacy vector system from Karl Clark.
F31S dihydrofolate reductase methotrexate-selection cassette	Watanabe et al, 2015; incorporated into the CAR donor construct in this study	As described in the CAR donor construct
Binder-GFP11-TwinStrep constructs for cell-free protein synthesis	This study; Gene Fragments from Twist Bioscience	Binder sequences/data: https://doi.org/10.5281/zenodo.20451404
Control scFv CAR and cell-free expression constructs	This study	1E4 antibody originally, https://patents.google.com/patent/WO2017011316A1/en
**Antibodies**		
Anti-Whitlow 218 linker-AlexaFluor 488 antibody (anti-218L-AF488; 1:50)	Cell Signaling Technology	Clone: E3U7Q / catalog no. 55809S
Anti-human TCR alpha/beta-APC antibody (1:50)	Miltenyi Biotec	Clone: REA652 / catalog no. 130-113-535
Anti-human IL2-PE REA antibody (1:50)	Miltenyi Biotec	Clone: REA689 / catalog no. 130-111-303
Anti-human IFN-gamma-Vio R667 REA antibody (1:50)	Miltenyi Biotec	Clone REA1343 / catalog no. 130-133-383
Fluorochrome-conjugated REA intracellular isotype controls (1:50)	Miltenyi Biotec	Clone REA293 / catalog nos. 130-118-347 (PE) 130-134-099 (Vio R667)
**Oligonucleotides and other sequence-based reagents**		
Codon-optimized oligonucleotide pool encoding the 12,000 submitted 80-aa binder sequences	Twist Bioscience; this study	Sequences/data: https://doi.org/10.5281/zenodo.20451404
TRAC-targeting and Universal guide RNAs used for GeneWeld integration	GeneWeld method; Webber et al, 2023; Wierson et al, 2020	https://pmc.ncbi.nlm.nih.gov/articles/PMC5558614/ - origin of TRAC gRNA. Wierson et al 2020 is the origin of UgRNA
GeneWeld 48-nt left and right TRAC homology arms	This study	TRAC Homology Arms: 5’ side: AAAGAGGGAAATGAGATCATGTCCTAACCCTGATCCTCTTGTCCCACA 3’ side: GATATCCAGAACCCTGACCCTGCCGTGTACCAGCTGAGAGACTCTAAA
PCR primers used to amplify integrated binder barcodes and add Nextera adapters	This study	Nextera-12K NGS F: TCGTCGGCAGCGTCAGATGTGTATAAGAGACAGTTCTACCATGGCGCTGCCT Nextera-12K NGS R: GTCTCGTGGGCTCGGAGATGTGTATAAGAGACAGTTTGTCACAAGATTTGGGCTCGAG
Codon-optimized binder Gene Fragments and 3-prime linker/GFP11-TwinStrep fragment	Adaptyv Bio	Binder amino acid sequences/data: https://doi.org/10.5281/zenodo.20451404; tag-fragment sequence not available to the authors and likely available by Adaptyv upon request
**Chemicals, enzymes and other reagents**		
NEBuilder HiFi DNA Assembly Kit	New England Biolabs	E5520S
X-VIVO 15 medium	Lonza	04-418Q
Human AB serum (5% v/v)	Gemini Bio	100-512-100
Dynabeads for T-cell stimulation (3:1 bead:cell ratio)	Thermo Fisher Scientific	I40203D
P3 nucleofection reagent	Lonza	V4XP-3012
Recombinant human IL-7 and IL-15 (10 ng/mL each)	Bio-Techne	BT-007-AFL-025 BT-015-AFL-025
Methotrexate	Med Chem Express	IHY-14519
CryoStor CS10 cryopreservation medium	STEMCELL Technologies	07930
Mitomycin C (10 microg/mL for 2 h)	STEMCELL Technologies	73274
Quick-DNA Miniprep Plus Kit	Zymo Research	D4069
Herculase II Fusion DNA Polymerase	Agilent Technologies	600677
SPRIselect DNA Size Selection Reagent	Beckman Coulter	B23318
eBioscience Fixable Viability Dye eFluor 450 (1:500)	Invitrogen/Thermo Fisher Scientific	65-0863-14
Brefeldin A	Bio-Techne	1231/5
CytoFix/CytoPerm fixation and permeabilization kit	BD Biosciences	554714
CellTrace Violet cell-proliferation dye	Thermo Fisher Scientific	C34557
Qubit DNA Quantification Kit	Invitrogen/Thermo Fisher Scientific	Q32851
Prokaryotic cell-free protein synthesis system	Adaptyv Bio	
Full-length human CD20 protein, Flag/His-tagged, detergent-solubilized	ACROBiosystems	Cat. no. CD0-H52D5
Strep-Tactin XT	IBA Lifesciences	
Carboxymethylated SPR sensor chip	Adaptyv Bio	
Split-GFP complementation and affinity-based protein-quantification reagents	Adaptyv Bio	
**Software**		
edgeR	Bioconductor; Robinson et al, 2010	
FlowJo v10	BD Life Sciences	v10; RRID:SCR_008520
MMseqs2	Steinegger and Soeding, 2017	
Boltz-1	Wohlwend et al, 2025	
ESMFold	Lin et al, 2023	
Chai-1	Boitreaud et al, 2024	
DSSP	Dictionary of Secondary Structure in Proteins software	
Rosetta FastRelax and InterfaceAnalyzer	Rosetta Commons; Leman et al, 2020; Stranges & Kuhlman, 2013	
ProteinMPNN, SolubleMPNN and ESM-2 scoring	Dauparas et al, 2022; Lin et al, 2023	
UCSF ChimeraX	UCSF Resource for Biocomputing, Visualization, and Informatics	
Biopython and peptide.bio solubility model	Biopython; peptide.bio	
ipSAE, AFM-LIS, pDockQ and pDockQ2	Dunbrack Jr, 2025; Kim et al, 2024; Bryant et al, 2022; Zhu et al, 2023	Implementations archived with analysis code
Adaptyv Fitting software	Adaptyv Bio	
Anabel	Krämer et al, 2019	
Custom competition-analysis and feature-generation code	This study; github.com/kosonocky/bits-to-binders	https://doi.org/10.5281/zenodo.20451404
**Other**		
Lonza nucleofection X Unit instrument (program EO-115)	Lonza	
G-Rex 6-well cell-culture plates	ScaleReady	80240 M
MACSQuant Analyzer 16 flow cytometer	Miltenyi Biotec	
ZAG DNA Analyzer	Agilent Technologies	
AVITI24 sequencing platform	Element Biosciences; University of Minnesota Genomics Center	
LSA XT high-throughput SPR platform with 96-channel array head	Carterra	
Gator Pro BLI system	Gator Bio	
Biomedical Research Computing Facility	University of Texas at Austin, Center for Biomedical Research Support	RRID:SCR_021979
Public study data and sequencing records	Zenodo; European Nucleotide Archive; GitHub	https://doi.org/10.5281/zenodo.20451404; ENA ERS29396515; github.com/kosonocky/bits-to-binders

### Selecting designs to test in each assay

Teams were asked to submit 500 sequences in order of confidence of success. In total, 28 teams submitted designs. The first 466 sequences from each team were selected to accommodate 12,000 total designs, including 16 designs chosen by LEAH Labs. Some teams submitted less than 466 designs or submitted duplicates, resulting in fewer total sequences: Foldsmiths: 465, Furman lab: 433, Nucleate UK London: 414, Perforators: 343, Zist Rayanesh: 308, Amigo Acids: 187, and Virtue Therapeutics: 48. 32 of the submitted sequences contained “X” as one of the amino acids, which was arbitrarily converted to “L” by Twist Bioscience.

The designs were sorted by −log10(FDR)*logFC, and the top ten non-redundant designs were chosen to proceed to the individual T cell functional assays. These designs came from the following teams: CAD Pro (1304), Perez Lab Gators (1506), LBM (1892), Nucleate UK London (2383), AIBI (3109), SNU LCDD (3494, 3718), Amigo Acids (5300), Binding Illini (5624), Schoeder Lab (5981). Sequence redundancy was determined by sharing at least 50% sequence identity (exact match).

For the binding affinity study, 100 designs were pulled from the three CD20-specific proliferation categories: 30 with ≥2-fold increase, 10 with ≥2-fold decrease, and 60 with no significant change. Sequences from each category were clustered with MMseqs2. For the ≥2-fold decrease category, 10 sequences were chosen in order of ascending −log10(FDR)*logFC, skipping if a sequence from the same cluster was already chosen. For the no significant change category, a sequence from each of 60 random clusters was randomly chosen. For the ≥2-fold increase category, the ten sequences that underwent individual functional assays were chosen, in addition to 20 sequences randomly chosen from the ≥2-fold increase category, ensuring that each sequence was chosen from a distinct cluster. In total, this amounted to 100 sequences along with the control scFv.

### Plasmid library construction

The CAR construct began with a CD8α leader sequence, “stuffer” sequence (deoxyribose-phosphate aldolase) in the place of a binder via HiFi Assembly (New England Biolabs), a CD28 hinge and transmembrane domain, a CD28 co-stimulatory domain, and a CD3ζ signaling domain (modified from Van Der Stegen et al, 2015). The F31S dihydrofolate reductase (DHFR) mutant was included after a P2A cleavage site, 3’ to the CAR construct, for further enrichment of the edited T cell population via selection with methotrexate (MTX) (Watanabe et al, 2015). The stuffer sequence was removed while the Whitlow 218 linker was introduced via HiFi Assembly (New England Biolabs). This “binderless” construct was onboarded for oligo-pool cloning of the 12,000 designed 80-amino-acid binders between the CD8α leader sequence and the 218 linker by Twist Bioscience.

### Pooled CAR-T generation and proliferation screen

CAR-T cell libraries were generated with GeneWeld (Webber et al, 2023; Wierson et al, 2020), which enables precision integration of the CAR construct through homology-mediated end joining by targeting the genome with CRISPR/Cas9 and the donor plasmid with a Universal gRNA to liberate the cargo and reveal 48 nucleotide homology arms. CAR variants were integrated near the N-terminus of the T cell receptor alpha chain, resulting in TRAC-negative, CAR-positive T cells.

Primary human T cells, previously isolated by BACS selection (Akadeum Life Science) from healthy-donor leukopaks (Sanguine Biosciences) and batch cryopreserved in CryoStor CS10 (STEMCELL Technologies). For experiments, T cells were thawed and allowed to recover overnight in T cell media comprised of X-Vivo15 + 5% AB serum and, after a brief stimulation with DynaBeads at a 3:1 bead to cell ratio, GeneWeld components were delivered via Nucleofection in P3 Buffer using the EO-115 program (Lonza). After a 72 h recovery in T cell media containing 10 ng/mL each of IL-7 and IL-15, CAR-T cells were transferred to G-Rex six-well plates (ScaleReady) for an additional 7 days of expansion under methotrexate selection. After sampling gDNA from 4E6 cells per replicate for NGS of the “Input” sample, 10E6 cells were subject to repeated challenge with 10E6 mitotically-inactivated (MI) - Raji-GFP target cells at an effector to target ratio of 1:1 in triplicate, once per week for 2 weeks in G-Rex six-well plates. Briefly, Raji-GFP cells were mitotically-inactivated with 10 ug/mL Mitomycin C (STEMCELL Technologies) for 2 h at a cell concentration of 1E6 cells/mL before washing three times and cryopreserving at 10-20E6 cells/mL in CryoStor CS10 for storage at −80 °C. Running in parallel with MI-Raji-GFP co-cultures, the same CAR-T cell libraries were cultured alone in a G-Rex six-well plate at 10E6 cells per well in triplicate. To control for false-positive outgrowth under similar culture conditions without target cells, 10E6 CAR-T cells were seeded into each G-Rex well in 10 mL, and the volume of each well was doubled to 20 mL after 7 days with T cell media containing 10 ng/mL each of IL-7 and IL-15, with MI-Raji-GFP targets for stimulated conditions. After 2 weeks, a minimum of 4E6 cells were harvested per replicate for genomic DNA isolation, using Quick-DNA Miniprep Plus Kit from Zymo Research, and NGS analysis in triplicate for both conditions (Joung et al, 2017). PCR was done with Herculase II Fusion DNA Polymerase from Agilent to add Nextera adapters. PCR purification was done using the SPRIselect DNA Size Selection Reagent from Beckman Coulter. Samples were submitted to the University of Minnesota Genomics Center for further library preparation and sequencing using the Element AVITI24 sequencing platform.

Variants were counted, and those represented by fewer than 25 reads in any replicate were excluded from further analysis. For each construct, CD20-specific proliferation was defined as the ratio of normalized barcode abundance in the CD20-positive condition relative to CAR-T cells alone as a control for false-positive, non-specific expansion. Statistical significance was assessed using edgeR (Robinson et al, 2010), and false discovery rates (FDRs) were controlled at 5%.

### CAR-T cell immunophenotyping by flow cytometry

Following methotrexate selection, CAR-T cells were characterized by multi-parameter flow cytometry. 1E5 cells were harvested into a round-bottom 96-well plate and washed with ice-cold MACS Buffer (PBS supplemented with 0.5% BSA and 2 mM EDTA). Surface antigen staining was performed by incubating cells with a fluorophore-conjugated antibody cocktail targeting the CAR (anti-218L-AlexaFluor 488, Cell Signaling Technologies) and the TCR (anti-TCRa/b-APC, Miltenyi Biotec), each at a 1:50 dilution in MACS buffer for 10 min at 4 °C in the dark. To assess viability and exclude non-viable cells from analysis, cells were washed with MACS buffer and stained with eBioscience Fixable Viability Dye eFluor 450 (Invitrogen) at a 1:500 dilution for 10 min at 4 °C. After two final washes, cells were resuspended in MACS buffer and acquired on a MACSQuant Analyzer 16 flow cytometer (Miltenyi Biotec). Data were analyzed using FlowJo software (v10) with the following hierarchical gating strategy: (1) Identification of lymphocytes based on forward scatter (FSC-A) and side scatter (SSC-A) to exclude debris, followed by (2) doublet discrimination via FSC-A vs FSC-H to ensure single-cell analysis, and (3) gating on the viability dye-negative population to exclude dead cells, allowing (4) CAR and TCR subset and expression analysis within the live cell population. Controls included Mock-transfected T cells (TCR + CAR−) and unstained (FMO) samples that did not receive anti-CAR (218 L) or anti-TCR antibodies.

### Specific lysis of individual T cell functional assay

CD20+ target cells -specific cytotoxicity was evaluated after co-incubating 25,000 CAR-T cells per well with CD20-positive target cells (Raji-GFP) and CD20-negative controls (K562-GFP) at varying effector-to-target ratios. After 48 h, specific lysis was quantified by counting the number of GFP+ target cells remaining per well after co-culture with CAR-T cells or untransfected controls, relative to target cells cultured alone as no lysis controls. The volumetric MACSQuant Analyzer 16 flow cytometer (Miltenyi Biotec) was used to obtain absolute cell counts per well. Percent targets killed = [1 - (number of GFP+ targets remaining/target cells alone) × 100%]. Specific lysis was determined by subtracting the percentage of CD20-negative K562-GFP targets killed from the percentage of Raji-GFP (CD20+) targets killed under each condition. Thus, multiple effector-to-target ratios allowed for AUC analysis to compare specific lysis of CD20+ targets (Raji^AUC^-K562^AUC^) between individual CAR-T clones of interest and control T cells.

### Cytokine generation in individual T cell functional assay

T cells were cultured alone or co-cultured with Raji-GFP target cells at a 1:1 E:T ratio for 16 h before adding Brefeldin A (Bio-Techne, 10 ug/mL) for an additional 4–5 h. After activation, cells were harvested, washed and stained with eBioscience Fixable Viability Dye eFluor 450 (Invitrogen) at a 1:500 dilution for 10 min at 4 °C to exclude non-viable cells from analysis before fixation, permeabilization, and intracellular cytokine staining using CytoFix/CytoPerm kit (BD Biosciences). Specifically, anti-IL2-PE and anti-IFNg-Vio R667 REA antibodies, along with fluorochrome-conjugated REA isotype controls (1:50 dilution, Miltenyi Biotec), were utilized to enumerate cytokine-producing T cells with a MACSQuant Analyzer 16 flow cytometer (Miltenyi Biotec). Fluorochrome-conjugated REA Intracellular isotype antibodies were used as controls to assess the percentages of T cells producing either IL2, IFNγ, or both, which were added together to reflect the total frequency of cytokine-producing T cells.

### Expansion and proliferation of individual T cell functional assays

CAR-T cell division and cumulative expansion were tracked over 5 days after stimulation with CD20-positive targets at varying effector-to-target ratios. Expansion was quantified as the total viable CAR-T yield 5 days after stimulation using volumetric sampling for accurate cell enumeration with the MACSQuant Analyzer 16 flow cytometer. T cell proliferation over 5 days was simultaneously inferred from cell division rates measured by the change in the mean fluorescence intensity (MFI) of Cell Trace Violet (Thermo Fisher Scientific) in T cells labeled according to the manufacturer’s instructions. Multiple effector-to-target (E:T) ratios, along with no MI-Raji target controls, allowed for AUC analysis to compare expansion and proliferation between individual CAR-T clones of interest and control T cells.

### Cell-free expression of binder variants for binding affinity

DNA constructs encoding the binder variants were designed by reverse-translating the protein sequences and optimizing them for manufacturability and yield using codon optimization algorithms. Each construct was fused to a C-terminal GFP11-TwinStrep (TST) tag to enable split-GFP-based expression quantification and affinity capture for kinetic characterization. Optimized variant sequences and the 3′ fragment encoding linker and affinity tags were ordered as Gene Fragments (Twist Bioscience). Constructs were assembled using the NEBuilder HiFi DNA Assembly Kit (NEB) in 2 μL reactions. Assembly quality was verified by capillary electrophoresis (Agilent ZAG DNA Analyzer), and DNA concentrations were quantified using the Qubit DNA Quantification Kit (Invitrogen).

Protein expression was performed in 8 μL reactions using an optimized prokaryotic cell-free protein synthesis (CFPS) system with 4 nM assembled DNA template. Reactions were incubated at 37 °C for 8–12 h. Soluble expression was confirmed using a split-GFP complementation assay, and protein yields were normalized using an affinity-based quantification assay. Crude lysates were used directly for downstream kinetic measurements.

### Surface plasmon resonance (SPR) affinity characterization

Binding kinetics were characterized using SPR on a Carterra LSA XT high-throughput platform. A carboxymethylated chip surface was functionalized with Strep-Tactin XT (IBA Lifesciences) using EDC/NHS chemistry. TwinStrep-tagged binder variants (from crude CFPS lysates diluted 125-fold) were captured onto the surface using the 96-channel array head (750 s capture, 600 s baseline).

The target antigen, full-length human CD20 (Acro Biosystems, CD0-H52D5; Flag/His-tagged, detergent-solubilized), was reconstituted in running buffer (50 mM HEPES, 150 mM NaCl, DDM + CHS, pH 7.5) and injected in solution over the immobilized variants. CD20 was tested at five half-log serial dilutions starting from 1000 nM. Measurements were performed in triplicate using multi-cycle kinetics, consisting of a 60 s baseline, 300 s association, and 600 s dissociation. Surfaces were regenerated with 10 mM glycine-HCl (pH 1.5) between full concentration series.

### Bio-layer interferometry (BLI)

A subset of variants was additionally tested by BLI on a Gator Pro system using the same CD20 preparation and detergent-containing buffer (50 mM HEPES, 100 mM NaCl, DDM + CHS). Binder variants were immobilized, and CD20 was run in solution as the analyte. Assay steps consisted of 60 s baseline, 120 s loading, 120 s baseline, 60 s association, and 300 s dissociation.

### Data processing and kinetic analysis

Sensorgrams were reference- and baseline-corrected and globally fitted to a 1:1 Langmuir binding model using Adaptyv Fitting software when signal quality permitted. Kinetic parameters (*KD, kon, koff*) were extracted from global fits across concentrations. When global fitting was not feasible, alternative models were applied, and parameters were selected based on fit quality.

Variants were classified as binders based on quantifiable kinetic fits. In cases where fitting was not reliable, but the association-phase signal exceeded 300% of the negative control, binding was assigned based on signal magnitude and estimated using Anabel (Krämer et al, 2019).

### Computational feature generation and statistical modeling

For each submitted binder, we computed a panel of >400 features from the nucleotide and amino acid sequences (Appendix Information B). Sequence-derived metrics included nucleotide and amino acid composition (for example, GC content), k-mer and repeat statistics (including homopolymers and dipeptide repeats), and Shannon entropy at the DNA and protein levels to quantify sequence complexity. Structure-informed features were derived from Boltz-1, ESMFold, and Chai-1 predictions of binder–CD20 complexes. These included model confidence statistics (e.g., pLDDT and ipTM), global and per-residue structural descriptors (e.g., secondary-structure composition and predicted disulfide content), and interface-focused metrics computed on the predicted complexes (e.g., contact counts and interface energy or interaction scores).

Features were analyzed against four outcomes: detection in the Twist plasmid pool quality report, recovery by NGS after the pooled proliferation assay, and CD20-specific change in proliferation. For binary outcomes, we trained logistic regression models using stratified fivefold cross-validation and evaluated performance with ROC-AUC. No class weighting or resampling was applied. To mitigate method-specific confounding, analyses were repeated on restricted subsets when appropriate (e.g., only ProteinMPNN/SolubleMPNN-generated designs). Feature relevance was assessed by comparing cross-validated performance across individual features and by inspecting class-conditional feature distributions.

## Supplementary information

Appendix

Table EV1

Table EV2

Table EV3

Table EV4

Peer Review File

Source data Fig. 2

Source data Fig. 3

Source data Fig. 4

Source data Fig. 5

Figure EV1 Source Data (pt 1)

Figure EV1 Source Data (pt 2)

Figure EV2 Source Data

Figure EV3 Source Data

Figure EV4 Source Data

Figure EV5 Source Data

Appendix source data

Expanded View Figures

## Data Availability

All data associated with this study is publicly available on Zenodo at https://doi.org/10.5281/zenodo.20451404. Summarized data used for the analysis can also be found at github.com/kosonocky/bits-to-binders. All sequencing data has been deposited in the European Nucleotide Archive (https://www.ebi.ac.uk/ena/browser/view/ERS29396515?dataType=SAMPLE) and supporting datasets. The source data of this paper are collected in the following database record: biostudies:S-SCDT-10_1038-S44320-026-00246-1.

## Code availability

Code used in the competition analysis is publicly available on Zenodo at https://doi.org/10.5281/zenodo.20451404 and at github.com/kosonocky/bits-to-binders. Code used by the competitors is listed in Appendix Information E, when available. Inquiries on the competitors’ code should be directed to the contacts listed therein.
