# Supplementary material for: Validation and analysis of 12,000 AI-driven CAR-T designs in the Bits to Binders competition
Source: Mol Syst Biol. 2026 Oct 8;22(11):1688–712. doi: 10.1038/s44320-026-00246-1 (PMC13728253; doi:10.1038/s44320-026-00246-1)
Supplement: Supplementary file 1 [file 44320_2026_246_MOESM18_ESM.pdf]

Expanded View Figures

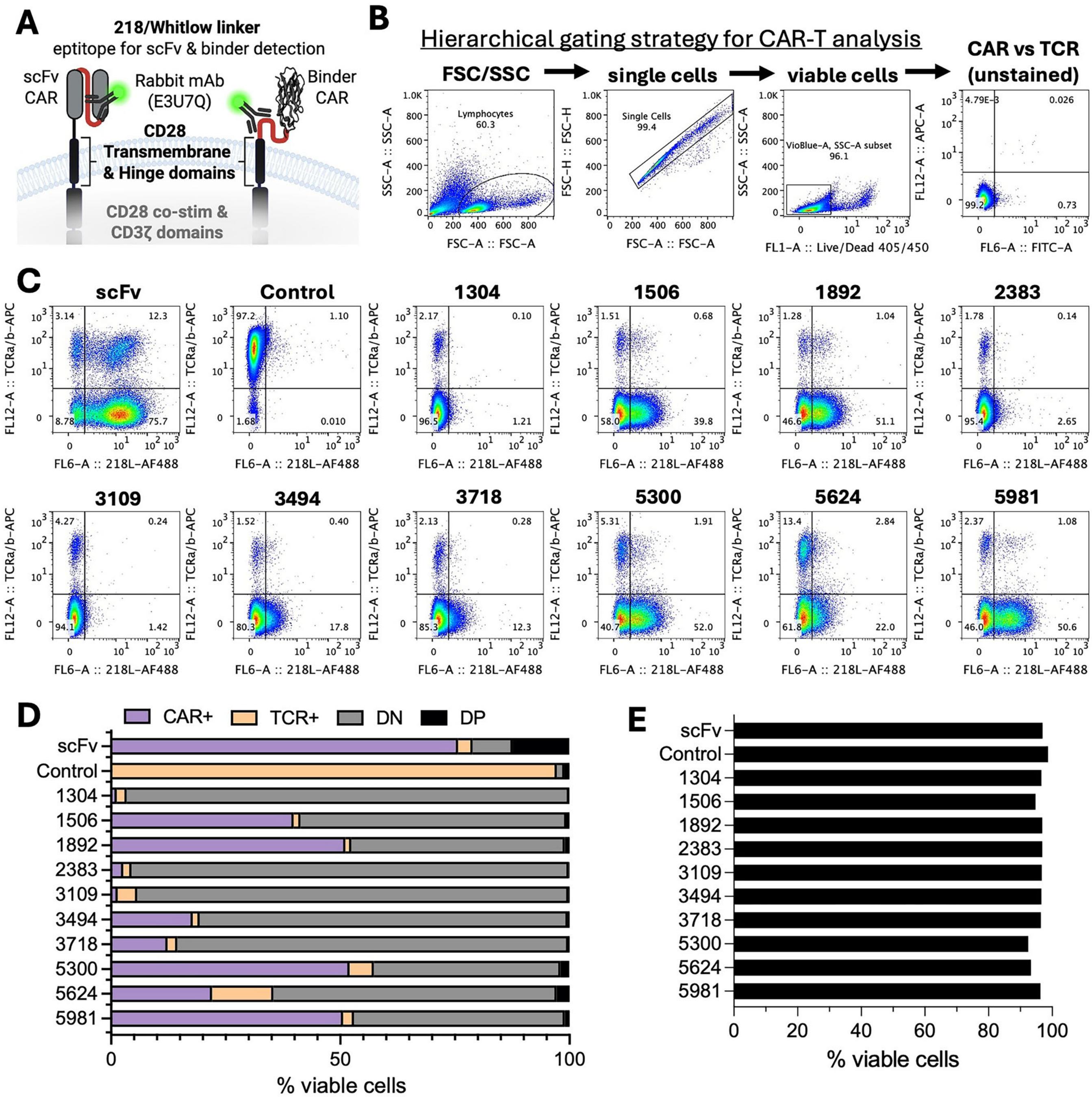

Figure EV1. CAR and TCR expression determined by flow cytometry.

(**A**) 218/Whitlow linker present in the scFv was designed into the binder construct as an antibody epitope. (**B**) Hierarchical gating strategy for viable CAR-T enumeration, as shown in (**C**) scatter plots depicting CAR and TCR surface expression for controls and binder variant CAR-T cells. (**D**) graphical representation of flow cytometry data showing T cell populations expressing CAR, TCR, neither, or both. (**E**) Viability assessment of controls and binder variant CAR-T cells. Source data are available online for this figure.

© The Author(s)

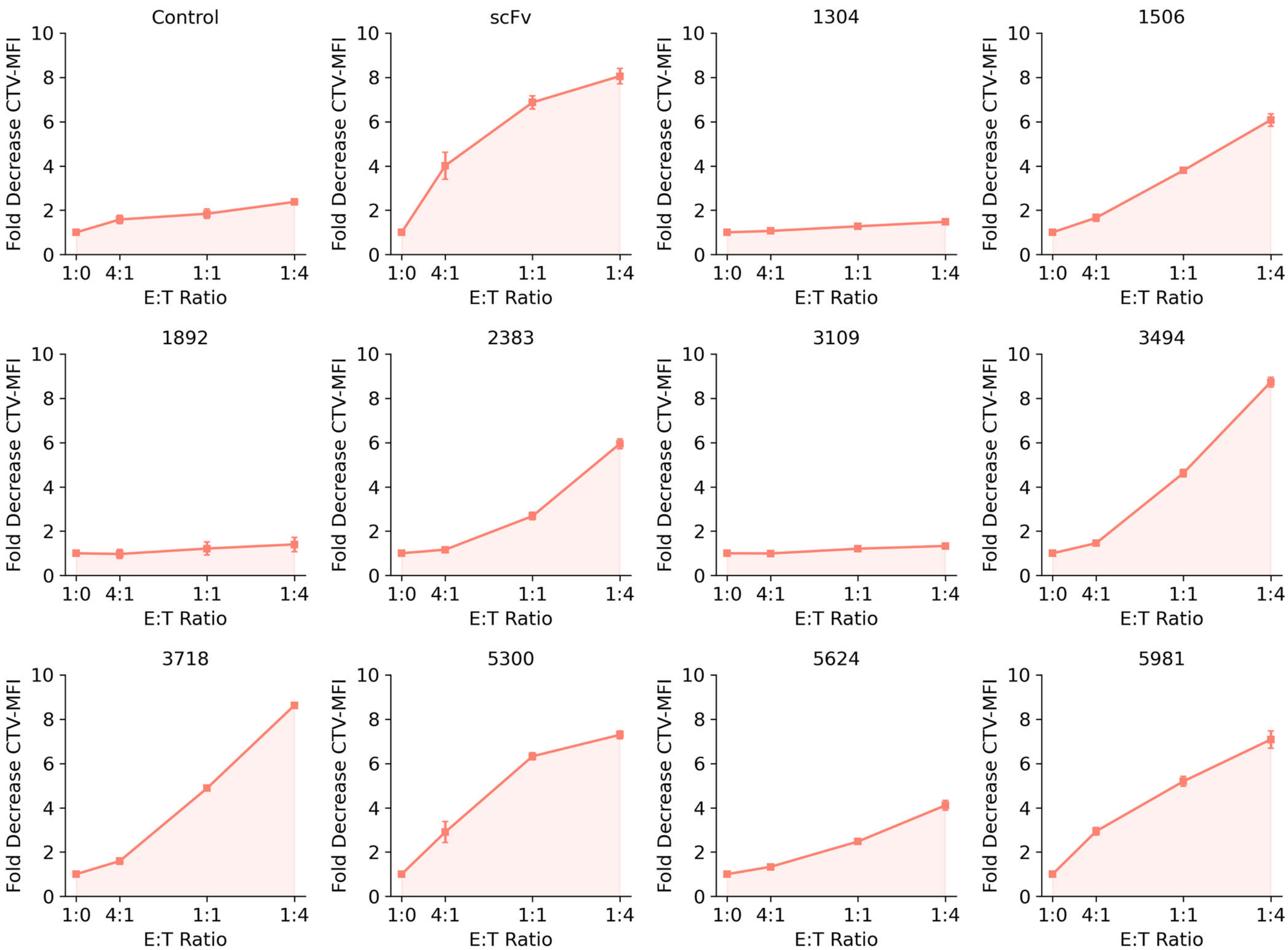

Figure EV2. Proliferation via a decrease in CTV-MFI.

Proliferation was measured over 5 days following stimulation with Raji (CD20+) targets across multiple E:T ratios by decrease in median fluorescence intensity (MFI) of the cell trace violet (CTV) signal. Data represent three technical replicates from one donor; bars indicate standard error centered on the mean. Source data are available online for this figure.

 © The Author(s)

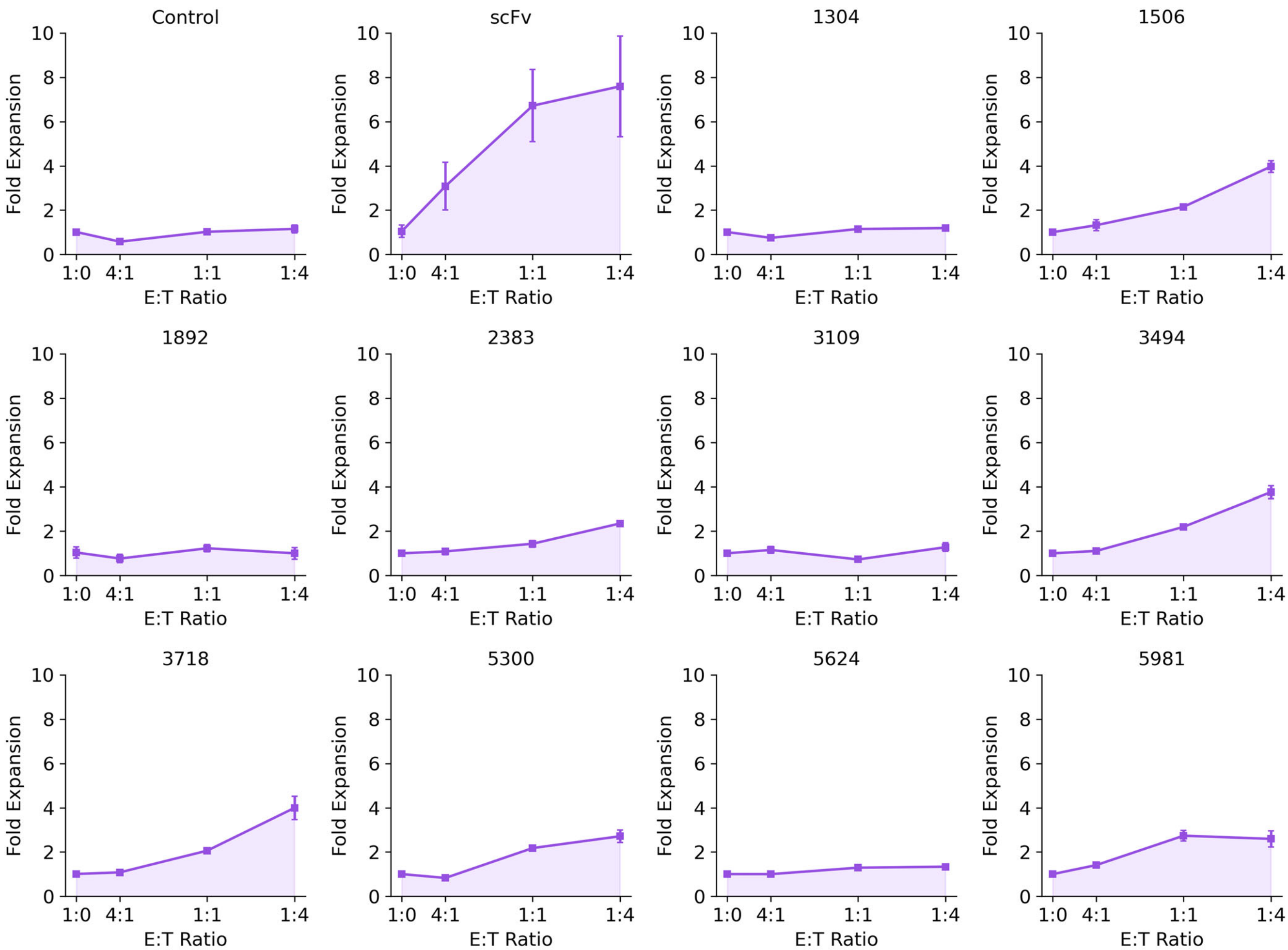

Figure EV3. Fold expansion by cell counts.

Total CAR-T expansion was measured over 5 days following stimulation with Raji (CD20+) targets across multiple E:T ratios by total viable CAR-T yield using volumetric flow cytometry. Data represent three technical replicates from one donor; bars indicate standard error centered on the mean. Source data are available online for this figure.

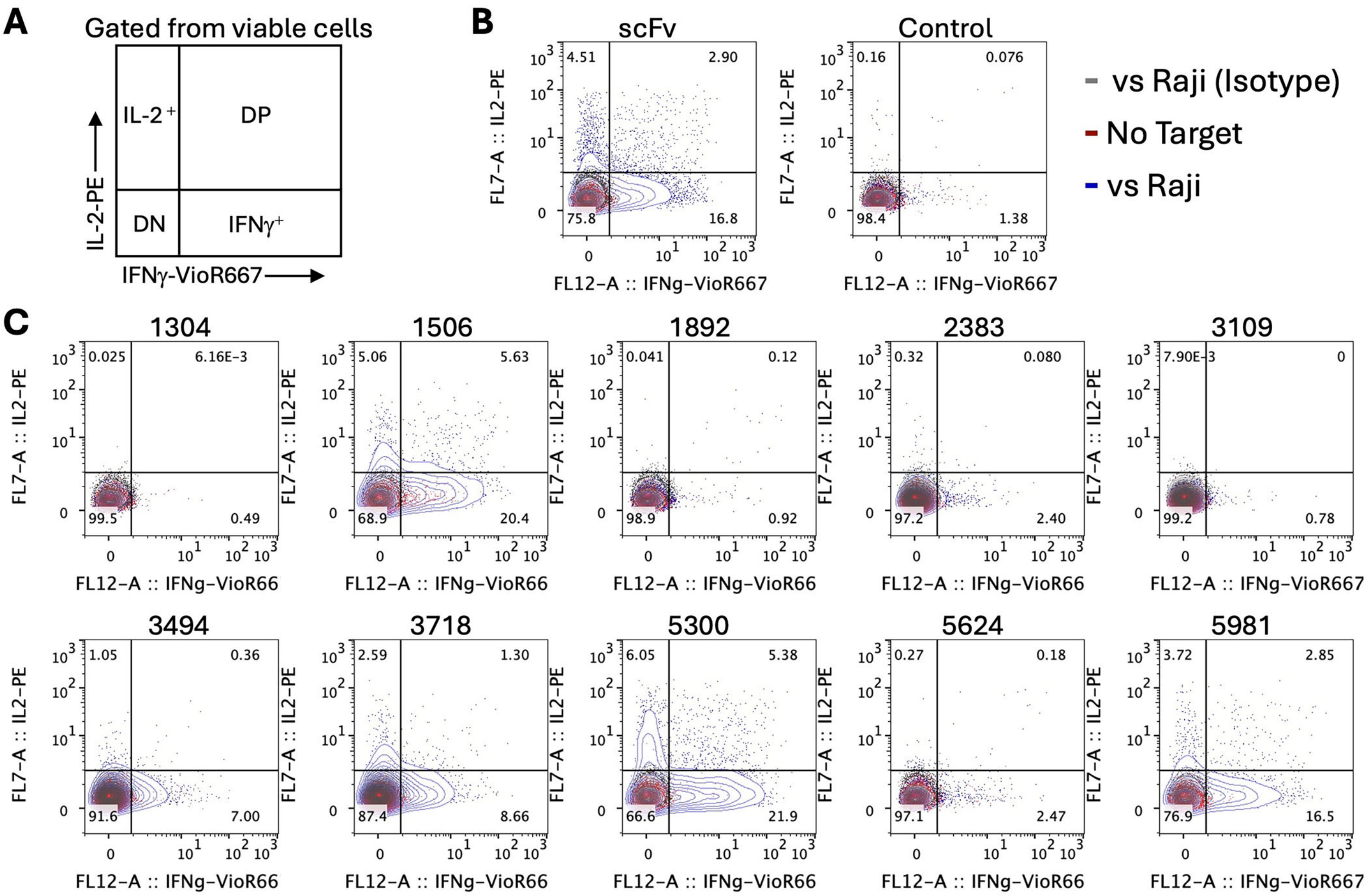

Figure EV4. CAR-T cell cytokine generation induced by CD20+ Raji target cells.

(**A**) Gating strategy illustrating cytokine-generating populations as analyzed by flow cytometry. (**B**) CD20 scFv CAR and Control (unedited, TCR + CAR-neg T cells), were stained with fluorochrome-conjugated antibodies after incubating alone (red) or in co-culture with Raji target cells. Specifically, anti-IFNg and anti-IL2 antibodies, or the requisite isotype antibody controls, were used to analyze both controls and (**C**) individual CD20 binders as CAR-T cells. Source data are available online for this figure.

 © The Author(s)

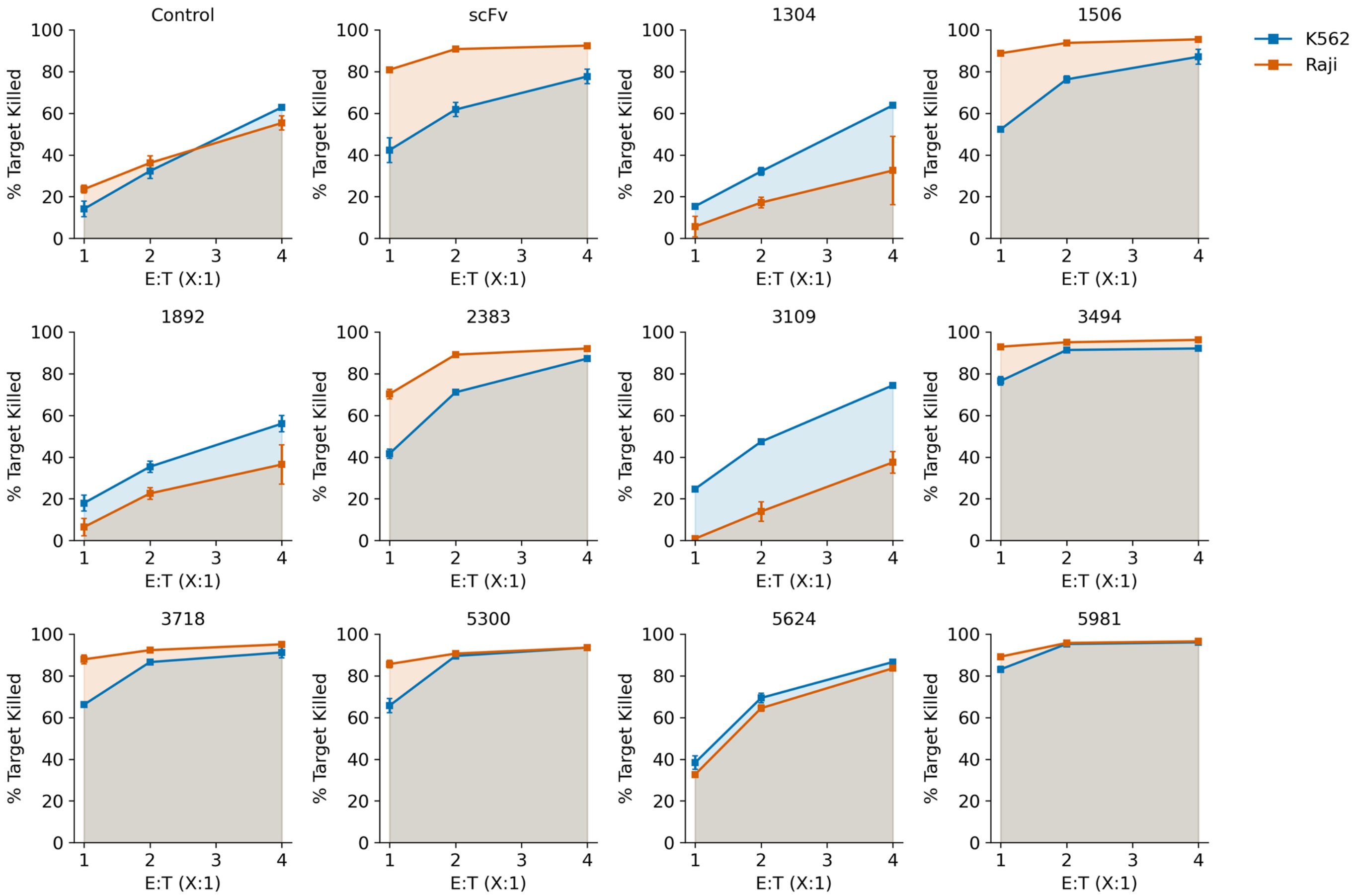

Figure EV5. Raji-specific cytotoxicity vs K562 control.

GFP assay readouts across multiple E:T ratios for Raji (CD20+) and K562 (CD20−) targets, overlaid for direct comparison. Data represents three technical replicates from one donor, bars indicate standard error centered on the mean. Source data are available online for this figure.

© The Author(s)
