# Supplementary material for: Validation and analysis of 12,000 AI-driven CAR-T designs in the Bits to Binders competition
Source: Mol Syst Biol. 2026 Oct 8;22(11):1688–712. doi: 10.1038/s44320-026-00246-1 (PMC13728253; doi:10.1038/s44320-026-00246-1)
Supplement: Supplementary file 3 [file 44320_2026_246_MOESM6_ESM.pdf]

molecular systems biology

Validation and analysis of 12,000 AI-driven CAR-T designs in the *Bits to Binders* competition

Clayton Kosonocky, Alex Abel, Aaron Feller, Amanda Cifuentes Rieffer, Phillip Woolley, Jakub Lála, Daryl Barth, Tynan Gardner, *Bits to Binders Competitors*, Stephen Ekker, Andrew Ellington, Wesley Wierson, and Edward Marcotte

Corresponding author(s): Edward Marcotte (marcotte@utexas.edu)

Review Timeline:

Submission Date:	15th Jun 26
Editorial Decision:	8th Jul 26
Revision Received:	25th Aug 26
Editorial Decision:	2nd Sep 26
Revision Received:	3rd Sep 26
Accepted:	9th Sep 26

Editor: Jingyi Hou

Transaction Report:

(Note: With the exception of the correction of typographical or spelling errors that could be a source of ambiguity, letters and reports are not edited. Depending on transfer agreements, referee reports obtained elsewhere may or may not be included in this compilation. Referee reports are anonymous unless the Referee chooses to sign their reports.)

8th Jul 2026

Manuscript Number: MSB-2026-13898
Title: Validation and analysis of 12,000 AI-driven CAR-T designs in the *Bits to Binders* competition
Author: Clayton Kosonocky
Alex Abel
Aaron Feller
Amanda Cifuentes Rieffer
Phillip Woolley
Jakub Lála
Daryl Barth
Tynan Gardner
Stephen Ekker
Andrew Ellington
Wesley Wierson
Edward Marcotte

Dear Dr Marcotte,

Thank you again for submitting your work to Molecular Systems Biology. We have now received feedback from the three reviewers who evaluated your manuscript. As you will see in the reports below, they express enthusiastic support for your study, while also raising several - relatively minor- issues that will need to be addressed in a revised version of the manuscript.

I think the reviewers' comments are clear and straightforward, so there is no need to repeat the points listed below. All the issues raised by the reviewers need to be satisfactorily addressed. As you may already know, our editorial policy allows in principle a single round of major revision so it is essential to provide responses to the reviewers' comments that are as complete as possible. Please feel free to contact me in case you would like to discuss in further detail any of the issues raised by the reviewers.

On a more editorial level, we would ask you to address the following issues:

- Please provide a .docx formatted version of the manuscript text (including legends for main figures, EV figures and tables). Please make sure that the changes are highlighted to be clearly visible.

- Please provide individual production quality figure files as .eps, .tif, .jpg (one file per figure). When assembling figures, please refer to our figure preparation guideline in order to ensure proper formatting and readability in print as well as on screen: https://media.springernature.com/original/springer-cms/rest/v1/content/27825798/data/v1

-Please provide a .docx formatted letter INCLUDING the reviewers' reports and your detailed point-by-point responses to their comments. As part of the EMBO Press transparent editorial process, the point-by-point response is part of the Review Process File (RPF), which will be published alongside your paper.

-Please note that all corresponding authors are required to supply an ORCID ID for their name upon submission of a revised manuscript.

-We replaced Supplementary Information with Expanded View (EV) Figures and Tables that are collapsible/expandable online. A maximum of 5 EV Figures can be typeset. EV Figures should be cited as 'Figure EV1, Figure EV2" etc... in the text and their respective legends should be included in the main text after the legends of regular figures.

Additional Tables/Datasets should be labeled and referred to as Table EV1, Dataset EV1, etc. Legends have to be provided in a separate tab in case of .xls files. Alternatively, the legend can be supplied as a separate text file (README) and zipped together with the Table/Dataset file.

For the figures and tables that you do NOT wish to display as Expanded View figures, they should be bundled together with their legends in a single PDF file called *Appendix*, which should start with a short Table of Content. Each legend should be below the corresponding Figure/Table in the Appendix. Appendix figures and tables should be referred to in the main text as: "Appendix Figure S1, Appendix Figure S2, Appendix Table S1" etc. See detailed instructions regarding expanded view here: https://link.springer.com/journal/44320/submission-guidelines#cms-Expanded-View-data.

-Before submitting your revision, primary datasets (and computer code, where appropriate) produced in this study need to be deposited in an appropriate public database.

Please remember to provide a reviewer password if the datasets are not yet public.
The accession numbers and database should be listed in a formal "Data Availability" section (placed after Materials & Method) that follows the model below (see also https://link.springer.com/partners/embo-press/editorial-policies#Data%20availability%20statement). Please note that the Data Availability Section is restricted to new primary data that are part of this study.
Data availability
The datasets (and computer code) produced in this study are available in the following databases:
- RNA-Seq data: Gene Expression Omnibus GSE46843 (https://www.ncbi.nlm.nih.gov/geo/query/acc.cgi?acc=GSE46843)
- [data type]: [name of the resource] [accession number/identifier/doi] ([URL or identifiers.org/DATABASE:ACCESSION])
*** Note - All links should resolve to a page where the data can be accessed. ***

- At EMBO Press we ask authors to provide source data for the main manuscript figures. You will receive a separate email with instructions for providing source data with your revised manuscript, including how to upload and organize the files.
Additional information on source data and instruction on how to label the files are available < https://link.springer.com/journal/44320/submission-guidelines#cms-Source-data >

- Our journal encourages inclusion of *data citations in the reference list* to directly cite datasets that were re-used and obtained from public databases. Data citations in the article text are distinct from normal bibliographical citations and should directly link to the database records from which the data can be accessed. In the main text, data citations are formatted as follows: "Data ref: Smith et al, 2001". In the Reference list, data citations must be labeled with "[DATASET]". A data reference must provide the database name, accession number/identifiers and a resolvable link to the landing page from which the data can be accessed at the end of the reference. Further instructions are available at < https://link.springer.com/journal/44320/submission-guidelines#cms-Reference-guidelines >.

- We updated our journal's competing interests policy in January 2022 and request authors to consider both actual and perceived competing interests. Please review the policy (https://link.springer.com/partners/embo-press/editorial-policies#Competing%20interest%20disclosures) and update your competing interests if necessary.
Please use the heading "Disclosure statement and competing interests".

- All Materials and Methods need to be described in the main text using our 'Structured Methods' format. According to this format, the Methods section includes a Reagents and Tools Table (listing key reagents, experimental models, software and relevant equipment and including their sources and relevant identifiers) followed by a Methods and Protocols section describing the methods, ideally using a step-by-step protocol format. The aim is to facilitate adoption of the methodologies across labs.

Please download and fill our Reagents and Tools Table template (.docx), which you can find in our author guidelines: https://link.springer.com/journal/44320/submission-guidelines#structuredmethods.

When submitting your revised manuscript, please do not include the Reagents and Tools Table in the Methods section of the manuscript but upload it as a separate file choosing the file type "Reagent Table".

-Regarding data quantification:
Please ensure to specify the name of the statistical test used to generate error bars and P values, the number (n) of independent experiments (please specify technical or biological replicates) underlying each data point and the test used to calculate p-values in each figure legend. Discussion of statistical methodology can be reported in the materials and methods section, but figure legends should contain a basic description of n, P and the test applied.
Graphs must include a description of the bars and the error bars (s.d., s.e.m.).
Please also include scale bars in all microscopy images.

- Please provide a "standfirst text" summarizing the study in one or two sentences (approximately 250 characters, including space), three to four "bullet points" highlighting the main findings. Please also provide a visual abstract as a separate PNG file with dimensions of exactly 550 px (width) × 300 px (height) to highlight the paper on our homepage.
Please refer to published papers for examples.

When you resubmit your manuscript, please download our CHECKLIST (https://media.springernature.com/original/springer-cms/rest/v1/content/27825796/data/v1) and include the completed form in your submission.
Please note that the Author Checklist will be published alongside the paper as part of the transparent process .

If you feel you can satisfactorily deal with these points and those listed by the referees, you may wish to submit a revised version of your manuscript. Please attach a covering letter giving details of the way in which you have handled each of the points raised by the referees. A revised manuscript will be once again subject to review and you probably understand that we can give you no guarantee at this stage that the eventual outcome will be favorable.

I look forward to receiving the revised manuscript soon.

Kind regards,
Jingyi

Jingyi Hou, PhD
Senior Editor
Molecular Systems Biology

We realize that it is difficult to revise to a specific deadline. In the interest of protecting the conceptual advance provided by the work, we recommend a revision within 3 months (6th Oct 2026). Please discuss the revision progress ahead of this time with the editor if you require more time to complete the revisions. Use the link below to submit your revision:

https://msb.msubmit.net/cgi-bin/main.plex

IMPORTANT: Please read our guidelines for revised submissions: https://link.springer.com/journal/44320/submission-guidelines#cms-Revised-submissions

*** PLEASE NOTE *** As part of the EMBO Press transparent editorial process initiative (see our Editorial at https://dx.doi.org/10.1038/msb.2010.72), Molecular Systems Biology publishes online a Review Process File with each accepted manuscripts. This file will be published in conjunction with your paper and will include the anonymous referee reports, your point-by-point response and all pertinent correspondence relating to the manuscript. If you do NOT want this File to be published, please inform the editorial office at contact@molsystbiol.org within 14 days upon receipt of the present letter.

Reviewer #1:

Kosonocky, Abel, et al. report the Bits to Binders competition, a crowdsourced benchmarking effort in which 28 teams from 42 countries submitted 12,000 de novo 80-amino acid binder designs targeting the B cell antigen CD20 for use as CAR-T cell binding domains. This is a particularly fun study design due to the proliferation and ease of AI-enabled binder design tools. From these designs, binders were cloned into a standardized second-generation CAR scaffold, integrated at the TRAC locus in primary human T cells, and screened in a pooled proliferation assay against CD20+ Raji cells. 707 designs showed significant CD20-specific enrichment ({greater than or equal to}2-fold). The top ten non-redundant hits were validated individually across proliferation, expansion, cytokine production, and cytotoxic killing assays. The authors then perform an extensive post-hoc computational analysis of the 12,000-sequence dataset, identifying sequence and structural features, including K+E alpha-helix content, adenosine repeat density, GC content, and cysteine abundance, that predict expression failure or tonic signaling dysregulation. Simple filters based on these features nearly double the retrospective hit rate. The paper is novel, timely, and of broad interest to the protein design and cell therapy communities and will likely be one of the most highly cited papers from this journal over the coming years. I also would recommend it be featured with a corresponding News and Views from the journal.

I enthusiastically recommend its eventual publication but would ask for a few points to be addressed in a revised version:

1 / In Figure 1 / 2, it may be helpful to clearly emphasize these binders are not antibody/scFvs (as is more common with CARs), particularly by showing a ChimeraX structure of a minibiner, a clinical-grade scFv, and Cd20 all in complex to a) emphasize the relative size disparity and b) reinforce the different binding mode.
2 / Under the limitations, additional context would be helpful noting that only one scaffold was used. Specifically, discussing where CARs may gain or lose function depending on various components would help clarify the point https://www.frontiersin.org/journals/immunology/articles/10.3389/fimmu.2025.1664403/full. Said another way, could the authors help contextualize what may possibly happen with different scaffolds? What's the range of potential outcomes?
3/ Related work has implicated positively charge patches as drivers of tonic signalling in scFvs (https://pmc.ncbi.nlm.nih.gov/articles/PMC10156736/) and alpha helical bundled CARs (https://pmc.ncbi.nlm.nih.gov/articles/PMC12710756/). Does a similar result emerge if one determines CARs with tonic signaling (I would think an increased abundance / proliferation in the CD20- co-culture) ?

Minor:
1/ I think the JointDiff paper cited (#53) should instead be paper: https://pubmed.ncbi.nlm.nih.gov/41236386/

Other: we reviewed the code and data resources, which are in good shape.

Reviewer #2:

The authors present Bits to Binders competition that evaluates AI-driven de novo binder design in the functional context of a CAR-T cell rather than by binding affinity alone. Twenty-eight teams submitted 12,000 80-amino-acid binders against the extracellular domain of CD20 and screened in a pooled antigen-dependent proliferation assay. The authors identify 707 designs with significant CD20-specific enrichment, validate the top ten as individual constructs across expression, proliferation, expansion, cytokine, and cytotoxicity assays.
Overall, I think this is a valuable and honest piece of work. The premise, that binding affinity is a poor proxy for higher-order biological function and that the field needs functional benchmarks, is exactly right, and the execution is careful. It is also very useful that the organizational lessons of running such a competition are summarized in Table SC1, as this kind of practical knowledge is rarely written down and will be genuinely helpful for anyone attempting a similar effort in the future. My reservations are mostly about the strength of the causal claims drawn from an uncontrolled and heavily monocultured method pool, the risk of overfitting in the retrospective filters, the interpretation of the binding and specificity data, and a few framing issues. I will break these into major and minor points below.

Major points:
1. A theme that deserves its own explicit analysis is the considerable effort many teams spent improving structural or interface scores and whether they actually translated into functional outcome. The teams applied a wide array of confidence and interface filters, AlphaFold2, ColabFold, ESMFold, Rosetta energies, HDOCK, PDockQ, and so on, and yet the authors separately show that essentially none of these structure-derived scores predicted recovery or CD20-specific proliferation. This is arguably one of the most important messages of the paper, that optimizing structural metrics did not buy functional success, but it is currently distributed across the results rather than stated directly. I would recommenwd the authors analyze and state plainly whether teams that pushed hardest on structural scoring did any better functionally than those that did not, since this speaks to how the field is currently spending its effort and would be a valuable, actionable takeaway.
2. The retrospective filters that nearly double the success rate are derived and evaluated on the same 12,000-design dataset, which risks overfitting and makes the headline improvement optimistic. The thresholds quoted, less than 1.9 DNA entropy, at least 45 AA repeats, at least 8 EE repeats, at least 30% K+E alpha-helix residues, and the GC cutoffs, appear to have been chosen on this dataset and then applied back to it. To make the almost-doubling claim credible, the authors should demonstrate the filter on held-out data, either by cross-validation with the thresholds refit inside each fold, or ideally by validating on an independent set. The authors already cite a phage-display study that confirms the K+E signal, so applying their exact filter to that or another external dataset and reporting the transfer would considerably strengthen the practical recommendation.
3. The explanation offered for why most top designs failed to bind in the SPR assay is not the most likely one and should be reconsidered. The authors attribute non-binding to prokaryotic cell-free expression and to possible differences in disulfide formation or post-translational processing relative to the mammalian secretory pathway. However, most de novo binders of this size, 80 residues, are designed to be disulfide-free and do not depend on post-translational modification, so this explanation is unlikely to be the main factor. A more plausible interpretation is that these short binders require the CAR scaffold itself for productive presentation, that is, stabilization and correct display on the T cell receptor architecture, and simply do not fold or engage CD20 as isolated 80-mers in solution. This would also be consistent with the four functional-but-non-binding designs apparently relying on avidity or scaffold-dependent presentation. I would recommend the authors revise this passage.

Minor points:
4. The 80-amino-acid constraint deserves explicit discussion as a limitation of the competition design. At this length the accessible fold space is quite restricted, favoring small helical bundles and short mixed motifs, which limits both the structural diversity of what could be submitted and the functional ceiling, for example the achievable interface area, epitope coverage, and affinity. The authors already note the length difference relative to the 250-residue scFv when explaining underperformance, but they should discuss more directly how the 80-residue cap constrains fold diversity and therefore what conclusions can and cannot be drawn about the design methods themselves versus the length limit imposed on them.
5. The manuscript would benefit from a single summary table in the main text giving, for each of the 28 teams, the primary generative method, the sequence-design method, the number of designs, the DNA-detection rate, the recovery rate, and the hit rate. Much of this is spread across figures and supplementary information, and a consolidated table would make the methodological comparisons far easier to follow.
6. Building on the observation that the non-recovered sequences are short and almost entirely helical, one attractive structural hypothesis is that many of them are simply two-helix bundles or otherwise under-packed folds that do not form a stable hydrophobic core at this length. It would be interesting and cheap to test this by analyzing core compactness of the predicted structures, for example the number of buried hydrophobic residues, packing or radius-of-gyration relative to length, or a void or cavity metric, and asking whether the non-recovered helical designs are systematically less compact than the recovered ones. If so, this would complement the K+E and translational-interference explanation with a folding-stability explanation and would be a useful design flag in its own right.
7. Since the designs served as their own DNA barcodes, is there enough resolution in the pooled data to say anything about expression-level dependence of enrichment, for example whether higher-abundance barcodes at the input stage systematically enriched or depleted? This could help disentangle expression effects from genuine antigen-driven proliferation.

Reviewer #3:

This is an interesting paper that presents the results and analyses form the Bits to Binders competition, a crowd-source competition to design chimeric antigen receptors against a specified target, CD20. More than 12k designs from 28 teams were experimentally tested in a high-throughput assay, with hit rates between <1% and nearly 40%. A small number of top scoring sequences were individually tested. The paper contains useful observations about strategies for successful design and also, on reflection information about how to run these - very useful - competitions for evaluating design success rates. I very much hope these competitions will continue, including changes learnt in the design and experimental testing to give better feedback to all the design teams and community about where and why designs are succeeding and failing.

Designing chimeric antigen receptors (CAR) is a very biomedically important goal. However, the proliferation assay used requires CAR to (1) express as a fusion protein, (2) localise to the membrane (3) bind productively (and likely not too strongly), and (4) trigger downstream signalling for proliferation. Thus whilst a practical applied goal, it is perhaps not, in retrospect, the best design-goal (alone) to evaluate in a competition. Or, rather, in future iterations it might be very good to specify and also measure the intermediate design tasks to solve e.g. cell-surface expression and biophysical binding, not just downstream proliferation. Otherwise it is challenging to disentangle at which steps designs are failing and if this is the same or different for different teams and approaches. The authors mention the possibility of a non-monotonic relationship between binding affinity and proliferation, which would be particularly challenging as a design goal and for evaluating and optimising methods. It would be very useful if the authors could further elaborate on how well established this non-monotonic relationship is from data or previous work. If successful designs require low affinity binders, this should be specified. Indeed the task of designing affinity - rather than just binding - is a great problem to work on.

One limitation of the crowd-sourced approach - that the authors also highlight - is that many teams used very similar approaches. It might be interesting in future competitions to combine this crowdsourcing with more systematic evaluation of design parameters by one (or some) teams and/or encourage the use of a diversity of approaches.

"the rank ordering of the clearly successful versus failing designs is unlikely to change materially" Why is this?

Comments from the editor:

Please provide a .docx formatted version of the manuscript text (including legends for main figures, EV figures and tables). Please make sure that the changes are highlighted to be clearly visible.

We have made provided a .docx formatted version of the manuscript text and all changes have been made with green highlight.

Please provide individual production quality figure files as .eps, .tif, .jpg (one file per figure). When assembling figures, please refer to our figure preparation guideline in order to ensure proper formatting and readability in print as well as on screen: https://media.springernature.com/original/springer-cms/rest/v1/content/27825798/data/v1

We have provided individual production quality figures in .jpg format (one file per figure).

Please provide a .docx formatted letter INCLUDING the reviewers' reports and your detailed point-by-point responses to their comments. As part of the EMBO Press transparent editorial process, the point-by-point response is part of the Review Process File (RPF), which will be published alongside your paper.

We have provided a .docx formatted letter including the reviewers' reports and our detailed point-by-point responses to their comments.

Please note that all corresponding authors are required to supply an ORCID ID for their name upon submission of a revised manuscript.

Edward M Marcotte, the corresponding author, will provide an ORCID ID upon submission of the revised manuscript.

We replaced Supplementary Information with Expanded View (EV) Figures and Tables that are collapsible/expandable online. A maximum of 5 EV Figures can be typeset. EV Figures should be cited as 'Figure EV1, Figure EV2" etc... in the text and their respective legends should be included in the main text after the legends of regular figures.

We have designated five supplemental figures to be EV figures and have updated legends accordingly.

Additional Tables/Datasets should be labeled and referred to as Table EV1, Dataset EV1, etc. Legends have to be provided in a separate tab in case of .xls files. Alternatively, the legend can be supplied as a separate text file (README) and zipped together with the Table/Dataset file.

We have updated all relevant supplementary tables to be called EV tables.

*For the figures and tables that you do NOT wish to display as Expanded View figures, they should be bundled together with their legends in a single PDF file called *Appendix*, which should start with a short Table of Content. Each legend should be below the corresponding Figure/Table in the Appendix. Appendix figures and tables should be referred to in the main text as: "Appendix Figure S1, Appendix Figure S2, Appendix Table S1" etc. See detailed instructions regarding expanded view here: https://link.springer.com/journal/44320/submission-guidelines#cms-Expanded-View-data.*

We have reformatted all relevant supplemental figures and tables to be in the appendix and have updated legends accordingly.

Before submitting your revision, primary datasets (and computer code, where appropriate) produced in this study need to be deposited in an appropriate public database.
Please remember to provide a reviewer password if the datasets are not yet public.
The accession numbers and database should be listed in a formal "Data Availability" section (placed after Materials & Method) that follows the model below (see also https://link.springer.com/partners/embo-press/editorial-policies#Data%20availability%20statement). Please note that the Data Availability Section is restricted to new primary data that are part of this study.
# Data availability
The datasets (and computer code) produced in this study are available in the following databases:
RNA-Seq data: Gene Expression Omnibus GSE46843 (https://www.ncbi.nlm.nih.gov/geo/query/acc.cgi?acc=GSE46843)
[data type]: [name of the resource] [accession number/identifier/doi] ([URL or identifiers.org/DATABASE:ACCESSION])
**** Note - All links should resolve to a page where the data can be accessed. ****

All sequencing data have been deposited in the European Nucleotide Archive (ERS29396515) and supporting datasets, including all predicted structures, are available in a Zenodo repository. All computer code associated with the main manuscript is available on GitHub and Zenodo. We have updated the Data Availability section to state this clearly.

At EMBO Press we ask authors to provide source data for the main manuscript figures. You will receive a separate email with instructions for providing source data with your revised manuscript, including how to upload and organize the files. Additional information on source data and instruction on how to label the files are available <https://link.springer.com/journal/44320/submission-guidelines#cms-Source-data >

We have provided all source data used to generate the main manuscript figures in the requested format.

*Our journal encourages inclusion of *data citations in the reference list* to directly cite datasets that were re-used and obtained from public databases. Data citations in the article text are distinct from normal bibliographical citations and should directly link to the database records from which the data can be accessed. In the main text, data citations are formatted as follows: "Data ref: Smith et al, 2001". In the Reference list, data citations must be labeled with "[DATASET]". A data reference must provide the database name, accession number/identifiers and a resolvable link to the landing page from which the data can be accessed at the end of the reference. Further instructions are available at < https://link.springer.com/journal/44320/submission-guidelines#cms-Reference-guidelines >.*

We do not have any data citations to report.

We updated our journal's competing interests policy in January 2022 and request authors to consider both actual and perceived competing interests. Please review the policy (https://link.springer.com/partners/embo-press/editorial-policies#Competing%20interest%20disclosures) and update your competing interests if necessary. Please use the heading "Disclosure statement and competing interests".

We have reviewed the policy and have no changes to make to our existing competing interests statement.

All Materials and Methods need to be described in the main text using our 'Structured Methods' format. According to this format, the Methods section includes a Reagents and Tools Table (listing key reagents, experimental models, software and relevant equipment and including their sources and relevant identifiers) followed by a Methods and Protocols section describing the methods, ideally using a step-by-step protocol format. The aim is to facilitate adoption of the methodologies across labs. Please download and fill our Reagents and Tools Table template (.docx), which you can find in our author guidelines: https://link.springer.com/journal/44320/submission-guidelines#structuredmethods. When submitting your revised manuscript, please do not include the Reagents and Tools Table in the Methods section of the manuscript but upload it as a separate file choosing the file type "Reagent Table".

We have provided a reagents and tools table as a separate file according to the requested guidelines. We have also updated our methods and protocols to provide additional information.

Please ensure to specify the name of the statistical test used to generate error bars and P values, the number (n) of independent experiments (please specify technical or biological replicates) underlying each data point and the test used to calculate p-values in each figure legend. Discussion of statistical methodology can be reported

in the materials and methods section, but figure legends should contain a basic description of n, P and the test applied.

We have ensured that all statistical tests state the test type and number of replicates. We have ensured that all figure legends contain a basic description of n, P, and the test applied.

Graphs must include a description of the bars and the error bars (s.d., s.e.m.).

We have confirmed that all bars and errors bars are described in the legend.

Please also include scale bars in all microscopy images.

N/A

Please provide a "standfirst text" summarizing the study in one or two sentences (approximately 250 characters, including space), three to four "bullet points" highlighting the main findings. Please also provide a visual abstract as a separate PNG file with dimensions of exactly 550 px (width) × 300 px (height) to highlight the paper on our homepage. Please refer to published papers for examples.

We have added a standfirst text summarizing the study in one or two sentences as well as three-to-four bullet points highlighting the main findings. These are as follows:

Standfirst:
A global competition benchmarking 12,000 AI-designed CD20 binders in primary human CAR-T cells, linking design strategies and sequence features to a variety of antigen independent and dependent outcomes to help inform future protein design.

Key findings

- Twenty-eight teams applied various AI-driven workflows to design 12,000 putative CD20 binders for functional evaluation as CAR recognition domains.
- Pooled screening identified 707 designs with antigen-dependent enrichment, and individual assays validated CAR-T cell activity for 7 of the 10 leading designs.
- Functional outcomes were associated with design methodology and sequence features that affect DNA synthesis, cellular recovery and target-dependent activity.
- Retrospective sequence filters identified common failure modes and nearly doubled the proportion of designs exhibiting antigen-dependent proliferation.

We have also provided a visual abstract as a separate PNG file.

Reviewer #1:

Kosonocky, Abel, et al. report the Bits to Binders competition, a crowdsourced benchmarking effort in which 28 teams from 42 countries submitted 12,000 de novo 80-amino acid binder designs targeting the B cell antigen CD20 for use as CAR-T

cell binding domains. This is a particularly fun study design due to the proliferation and ease of AI-enabled binder design tools. From these designs, binders were cloned into a standardized second-generation CAR scaffold, integrated at the TRAC locus in primary human T cells, and screened in a pooled proliferation assay against CD20+ Raji cells. 707 designs showed significant CD20-specific enrichment ({greater than or equal to}2-fold). The top ten non-redundant hits were validated individually across proliferation, expansion, cytokine production, and cytotoxic killing assays. The authors then perform an extensive post-hoc computational analysis of the 12,000-sequence dataset, identifying sequence and structural features, including K+E alpha-helix content, adenosine repeat density, GC content, and cysteine abundance, that predict expression failure or tonic signaling dysregulation. Simple filters based on these features nearly double the retrospective hit rate. The paper is novel, timely, and of broad interest to the protein design and cell therapy communities and will likely be one of the most highly cited papers from this journal over the coming years. I also would recommend it be featured with a corresponding News and Views from the journal.

We thank the reviewer for recognizing the novelty and impact of our work.

I enthusiastically recommend its eventual publication but would ask for a few points to be addressed in a revised version:

1 / In Figure 1 / 2, it may be helpful to clearly emphasize these binders are not antibody/scFvs (as is more common with CARs), particularly by showing a ChimeraX structure of a minibiner, a clinical-grade scFv, and Cd20 all in complex to a) emphasize the relative size disparity and b) reinforce the different binding mode.

In Figures 1, 2, and 3, we have changed phrasing to say, "*de novo* minibinder-based CARs" and "80-amino-acid *de novo* protein minibinders" to clearly state that the length restriction and design scope. We have also introduced the phrase "*de novo* minibinders" where appropriate in the abstract, introduction, and results paragraph to make this clear. In the results section, we also added note on the relative size differences between the minibinders, clinical-grade scFvs, and CD20 to emphasize their size:

<u>Lines 96-97:</u> "Third, teams were tasked to design minibinders of exactly 80 amino acids, much shorter than a typical scFv (230-260 amino acids)".

Although we attempted so, we did not add a subpanel showing the difference between a clinical-grade scFv and a minibinder in complex with CD20 as we were unable to find a way to present this without the figure looking overly busy. We hope this is okay.

2 / Under the limitations, additional context would be helpful noting that only one scaffold was used. Specifically, discussing where CARs may gain or lose function depending on various components would help clarify the point https://www.frontiersin.org/journals/immunology/articles/10.3389/fimmu.2025.166440

3/full. Said another way, could the authors help contextualize what may possibly happen with different scaffolds? What's the range of potential outcomes?

In the limitations paragraph of the discussion, we have added the sentence:

Lines 481-486: "Alternative linker, hinge, transmembrane and signaling domains could alter binder orientation and surface expression, as well as antigen sensitivity, tonic signaling and persistence, potentially rescuing inactive designs or diminishing those that performed well in this scaffold (Bernard & Evgin, 2025)". This provides additional context for what might happen with these binders under different scaffolds.

3/ Related work has implicated positively charge patches as drivers of tonic signalling in scFvs (https://pmc.ncbi.nlm.nih.gov/articles/PMC10156736/) and alpha helical bundled CARs (https://pmc.ncbi.nlm.nih.gov/articles/PMC12710756/). Does a similar result emerge if one determines CARs with tonic signaling (I would think an increased abundance / proliferation in the CD20- co-culture) ?

We thank the reviewer for providing these findings and suggesting these analyses.

We first analyzed the mean coulombic potential of the Boltz-1 predicted binder bound-state structures using ChimeraX with Amber 20 recommended default atom charges. This measured the average electrostatic potential by the binder's atomic partial charges across its molecular surface. Though there was a significant difference ($p<1E-4$) between recovered and non-recovered sequences, the effect size was very small. For the proliferation outcomes, there was slightly significant ($p<1E-2$) higher coulombic potential among the depletion sequences, though this effect size was again relatively minor. We have provided plots for these in Appendix Figure S11.

We then computed the ratio of binder chain residues participating in alpha-helical bundles. There were significant differences between recovered and non-recovered sequences, as well as between the ≥2-fold increase, not significant, and ≥2-fold decrease sequences. There was a noticeable enrichment of alpha helical bundles in the non-recovered sequences, and further in the ≥2-fold compared, to the other groups. That said, the inner quartiles for these span most of the feature's quantitative range. We also provide these plots in Appendix Table S11.

1/ I think the JointDiff paper cited (#53) should instead be paper: https://pubmed.ncbi.nlm.nih.gov/41236386/

We have corrected this reference accordingly.

Other: we reviewed the code and data resources, which are in good shape.

We thank the reviewer for ensuring that our code and data is in good shape.

Reviewer #2:

The authors present Bits to Binders competition that evaluates AI-driven de novo binder design in the functional context of a CAR-T cell rather than by binding affinity alone. Twenty-eight teams submitted 12,000 80-amino-acid binders against the extracellular domain of CD20 and screened in a pooled antigen-dependent proliferation assay. The authors identify 707 designs with significant CD20-specific enrichment, validate the top ten as individual constructs across expression, proliferation, expansion, cytokine, and cytotoxicity assays.
Overall, I think this is a valuable and honest piece of work. The premise, that binding affinity is a poor proxy for higher-order biological function and that the field needs functional benchmarks, is exactly right, and the execution is careful. It is also very useful that the organizational lessons of running such a competition are summarized in Table SC1, as this kind of practical knowledge is rarely written down and will be genuinely helpful for anyone attempting a similar effort in the future. My reservations are mostly about the strength of the causal claims drawn from an uncontrolled and heavily monocultured method pool, the risk of overfitting in the retrospective filters, the interpretation of the binding and specificity data, and a few framing issues. I will break these into major and minor points below.

We thank the reviewer for recognizing the value our work and for providing valuable feedback. We agree on the importance of Table SC1 and have decided to move it up to be a main text table as we think that this organizational information would be valuable front-and-center rather than buried in the appendix.

Major Points:
1. A theme that deserves its own explicit analysis is the considerable effort many teams spent improving structural or interface scores and whether they actually translated into functional outcome. The teams applied a wide array of confidence and interface filters, AlphaFold2, ColabFold, ESMFold, Rosetta energies, HDOCK, PDockQ, and so on, and yet the authors separately show that essentially none of these structure-derived scores predicted recovery or CD20-specific proliferation. This is arguably one of the most important messages of the paper, that optimizing structural metrics did not buy functional success, but it is currently distributed across the results rather than stated directly. I would recommend the authors analyze and state plainly whether teams that pushed hardest on structural scoring did any better functionally than those that did not, since this speaks to how the field is currently spending its effort and would be a valuable, actionable takeaway.

We thank the reviewer for this suggestion as its corresponding analysis has led to very interesting and unexpected results. We were unable to determine a consistent and fair way to quantify structural effort, so we instead decided to correlate the teams' rank ordering (which frequently included structure-based scoring) with the various experimental outcomes. These data can be found in **Appendix Figure S5** and **Appendix Figure S6** and have summarized in the following paragraph in the results:

<u>Lines 287-299:</u> "During the solicitation, teams were asked to submit their sequences in

the rank order that they deemed would produce successful CARs, in which teams ranked by perplexity, folding scores, and other combined metrics. Rank ordering was significantly positively correlated with recovery for Binder Bot Engineers (ρ=0.20), Molecule Masters (ρ=0.17), and CAD Pro (ρ=0.14), and negatively correlated for Perez Lab Gators (ρ=-0.11), SNU LCDD (ρ=-0.12), Tumor Inhibitors (ρ=-0.12), Binding Illini (ρ=-0.14), Zist Rayanesh (ρ=-0.22), Evaneil (ρ=-0.41), and Foldsmiths (ρ=-0.51) (Appendix Figure S5). Rank ordering was not significantly positively correlated with successful proliferation for any teams, but it was significantly negatively correlated with successful proliferation for Binder Bot Engineers (ρ=-0.25) and Foldsmiths (ρ=-0.32) (Appendix Figure S6). The strong anticorrelations found in several teams, in which the lower-ranked designs were more successful, may be indicative of optimal CAR binding being moderate affinity, and further suggests that optimizing with structure-based metrics is not always optimal."

We think this data strongly suggests structure-based filtering as being largely inapplicable, and potentially even strongly detrimental, to the task of designing functional and expressible CARs. As such, we have made the following changes in the manuscript to more strongly imply that structure-based metrics are not always applicable to functional outcomes:

<u>Line 409:</u> "These findings exemplify how simple metrics can be more predictive of function than **structure-based or layered** deep learning-based scores."

<u>Lines 474-478:</u> "We additionally found little predictive power of common structure-based metrics for the functional outcomes of recovery and proliferation, consistent with the lack of positive correlation between most teams' submitted rank order and experimental outcomes, suggesting that optimizing solely for structure may not entail functional success."

> *2. The retrospective filters that nearly double the success rate are derived and evaluated on the same 12,000-design dataset, which risks overfitting and makes the headline improvement optimistic. The thresholds quoted, less than 1.9 DNA entropy, at least 45 AA repeats, at least 8 EE repeats, at least 30% K+E alpha-helix residues, and the GC cutoffs, appear to have been chosen on this dataset and then applied back to it. To make the almost-doubling claim credible, the authors should demonstrate the filter on held-out data, either by cross-validation with the thresholds refit inside each fold, or ideally by validating on an independent set. The authors already cite a phage-display study that confirms the K+E signal, so applying their exact filter to that or another external dataset and reporting the transfer would considerably strengthen the practical recommendation.*

We agree that a held-out dataset would be ideal to validate the proposed filter values. We investigated using the NVIDIA phage-display study but found that these sequences and their outcomes were not made publicly available, so this proved to not be an option.

We then investigated re-fitting the filters using a 5-fold cross-validation. After examining the team-wise distributions for each feature (added as **Appendix Figures S17 - S22**), we reasoned that a random sequence-based split was best for determining the threshold values. The following features were fit according to a 5-fold random sequence-based split cross validation, optimizing for holdout set recovery % while retaining at least 60% of the sequences: DNA GC content and DNA sequence entropy, number of adenosine repeats in the DNA, number of glutamate repeats, and the ratio of residues in (K+E) alpha helices. Additional filter results are shown for the exclusion of any sequences containing cysteine. The results of this cross validation are shown in **Appendix Figure S15** and the per-fold thresholds are shown in **Appendix Figure S16**. Our initially recommended values fell within the per-fold ranges for every feature except for the number of adenosine repeats (we initially recommended filtering out sequences ≥45 repeats). We have since adjusted this recommended value to fall within the per-fold thresholds and now recommend ≥43 repeats. That said, this adjustment did not change any of the sequences that were retained or removed, but we believe our threshold recommendations and doubling claims are now more justified. We thank the reviewer once again for the suggestion.

> *3. The explanation offered for why most top designs failed to bind in the SPR assay is not the most likely one and should be reconsidered. The authors attribute non-binding to prokaryotic cell-free expression and to possible differences in disulfide formation or post-translational processing relative to the mammalian secretory pathway. However, most de novo binders of this size, 80 residues, are designed to be disulfide-free and do not depend on post-translational modification, so this explanation is unlikely to be the main factor. A more plausible interpretation is that these short binders require the CAR scaffold itself for productive presentation, that is, stabilization and correct display on the T cell receptor architecture, and simply do not fold or engage CD20 as isolated 80-mers in solution. This would also be consistent with the four functional-but-non-binding designs apparently relying on avidity or scaffold-dependent presentation. I would recommend the authors revise this passage.*

We thank the reviewer for their suggestion. We agree that the lack of CAR scaffold is the most likely explanation for the binding / function discrepancy. That said, we wish to point out that the predicted structures of many sequences appear to contain explicitly designed disulfide bonds. Design 5838 is a particularly good example, in which Boltz-1 predicts it to form 3-4 disulfide bonds all within the binder chain. As such, we wish to leave in this sentence, and have placed it secondary to the comment on the CAR scaffold. This section has been revised as such:

<u>Lines 239-247</u> “One caveat to these results is that the binding assay tested the binders as isolated 80-mers rather than in the context of the CAR scaffold, and thus proper folding, display, and avidity effects may have failed to occur. Secondarily, these binders were created using prokaryotic cell-free protein synthesis which may have contributed differences in folding efficiency, disulfide formation (design 5838, for example, is predicted to form 3-4 disulfide bonds), and post-translational processing between a

prokaryotic lysate and the mammalian secretory pathway. As such, failure to bind in this assay may instead reflect differences in binder structural context and expression rather than design quality (Thomas et al, 2023)."

Minor points:
4. The 80-amino-acid constraint deserves explicit discussion as a limitation of the competition design. At this length the accessible fold space is quite restricted, favoring small helical bundles and short mixed motifs, which limits both the structural diversity of what could be submitted and the functional ceiling, for example the achievable interface area, epitope coverage, and affinity. The authors already note the length difference relative to the 250-residue scFv when explaining underperformance, but they should discuss more directly how the 80-residue cap constrains fold diversity and therefore what conclusions can and cannot be drawn about the design methods themselves versus the length limit imposed on them.

We thank the reviewer for this point. We have added a sentence to the discussion on the 80-amino-acid length constraint being a limitation of the competition design:

<u>Lines 508-510:</u> "Furthermore, the binder designs were limited to 80 amino acids, relative to a typical scFv (230-260 amino acids), which constrains fold diversity and thus the conclusions that can be drawn between generative methods."

5. The manuscript would benefit from a single summary table in the main text giving, for each of the 28 teams, the primary generative method, the sequence-design method, the number of designs, the DNA-detection rate, the recovery rate, and the hit rate. Much of this is spread across figures and supplementary information, and a consolidated table would make the methodological comparisons far easier to follow.

We agree that a team-wise summary table would be helpful for the reader and have added one to the main text (**Table 2)**. We have also introduced this table in the results section in the following sentence:

<u>Lines 266-267:</u> "A summary of the predominant methodologies and experimental outcomes for all 28 teams can be found in Table 2."

6. Building on the observation that the non-recovered sequences are short and almost entirely helical, one attractive structural hypothesis is that many of them are simply two-helix bundles or otherwise under-packed folds that do not form a stable hydrophobic core at this length. It would be interesting and cheap to test this by analyzing core compactness of the predicted structures, for example the number of buried hydrophobic residues, packing or radius-of-gyration relative to length, or a void or cavity metric, and asking whether the non-recovered helical designs are systematically less compact than the recovered ones. If so, this would complement the K+E and translational-interference explanation with a folding-stability explanation and would be a useful design flag in its own right.

We thank the reviewer for this suggestion. From the Boltz-1 predicted binder structures, we analyzed both radius of gyration and the hydrophobic surface fraction (fraction of surface belonging to hydrophobic residues).

Radius of gyration showed a significant difference between recovered and non-recovered, but the mean effect size was negligible. That said, there was a clear mass at around 35Å in the non-recovered sequences that is less pronounced in the recovered sequences. There were no significant differences between the proliferation outcomes according to radius of gyration. These plots are shown in **Appendix Figure S11**.

The hydrophobic surface fraction does have meaningful separation in both recovery and proliferation, though not in the expected direction. The recovered sequences have greater hydrophobic surface fraction (mean ~25%) than the non-recovered sequences (mean ~17%). For those that were recovered, the sequences that caused ≥2-fold increase in proliferation had the lowest hydrophobic surface fraction (mean ~22%), then the non-significant change in proliferation group (mean ~26%), and finally those that caused ≥2-fold decrease in proliferation had the highest hydrophobic surface fraction (mean ~28%). Upon closer examination, this feature is strongly inversely correlated with K+E ratio (Pearson $r = -0.58$, $p < 0.001$), which makes sense given that an increase in charged and polar residues would decrease surface hydrophobicity, thus explaining why the effect is the opposite of what we initially expected. These plots are provided in **Appendix Figure S11**.

> *7. Since the designs served as their own DNA barcodes, is there enough resolution in the pooled data to say anything about expression-level dependence of enrichment, for example whether higher-abundance barcodes at the input stage systematically enriched or depleted? This could help disentangle expression effects from genuine antigen-driven proliferation.*

We agree that input barcode abundance could influence apparent enrichment or depletion. However, the experiment did not include sequencing of the pooled population at an input timepoint, so we cannot directly test whether initial design abundance predicted subsequent outcome. The closest figure we have to answer this question is **Appendix Figure S2**, which shows that the assay has strong agreement among biological replicates in both the CD20- and CD20+ conditions, suggesting that substantial replicate-specific jackpot effects did not take place, but it cannot exclude systematic effects of initial abundance shared across replicates. We have clarified this limitation in the discussion section:

<u>Lines 484-486:</u> "Second, sequencing was not done on the pooled populations at an input timepoint, so it is unclear whether initial input abundance impacted subsequent proliferation outcomes."

<u>Reviewer #3:</u>

This is an interesting paper that presents the results and analyses form the Bits to Binders competition, a crowd-source competition to design chimeric antigen receptors against a specified target, CD20. More than 12k designs from 28 teams were experimentally tested in a high-throughput assay, with hit rates between <1% and nearly 40%. A small number of top scoring sequences were individually tested. The paper contains useful observations about strategies for successful design and also, on reflection information about how to run these - very useful - competitions for evaluating design success rates. I very much hope these competitions will continue, including changes learnt in the design and experimental testing to give better feedback to all the design teams and community about where and why designs are succeeding and failing.

We thank the reviewer for recognizing the value of our study, as well as competitions in general.

Designing chimeric antigen receptors (CAR) is a very biomedically important goal. However, the proliferation assay used requires CAR to (1) express as a fusion protein, (2) localise to the membrane (3) bind productively (and likely not too strongly), and (4) trigger downstream signalling for proliferation. Thus whilst a practical applied goal, it is perhaps not, in retrospect, the best design-goal (alone) to evaluate in a competition. Or, rather, in future iterations it might be very good to specify and also measure the intermediate design tasks to solve e.g. cell-surface expression and biophysical binding, not just downstream proliferation. Otherwise it is challenging to disentangle at which steps designs are failing and if this is the same or different for different teams and approaches. The authors mention the possibility of a non-monotonic relationship between binding affinity and proliferation, which would be particularly challenging as a design goal and for evaluating and optimising methods. It would be very useful if the authors could further elaborate on how well established this non-monotonic relationship is from data or previous work. If successful designs require low affinity binders, this should be specified. Indeed the task of designing affinity - rather than just binding - is a great problem to work on.

First, we agree that the multi-step goal of this competition lends difficulties to uncovering granular trends in the data. This competition was largely built around the high capacity of the proliferation assay that allowed us to evaluate 12,000 designs. We attempted to resolve some of the finer-grained questions by measuring cell-surface expression and biophysical binding for the top designs, though we could not do this for all 12,000 designs. An explanation of our thinking process here has been described in **Table SC1** (now **Table 1**).

In regards to the non-monotonicity of binding and function: at the kickoff event, we specified the nonlinear relationship between binding affinity and proliferation, stressing that stronger binding is not always better in this case. This has been mentioned at the beginning of the results section, followed by two sources that have found this behavior:

Lines 93-96: "Second, success was dependent on achieving sufficient affinity to effectively recognize the target, but not so much that the CAR-T cell becomes exhausted (Zebley & Youngblood, 2022; Benzaoui et al, 2022)."

To help clarify this point further, we have added the following sentences at the end of this paragraph (in bold):

Lines 229-238: "This weak signal is perhaps unsurprising, since optimal CAR function often occurs within a moderate range of binding constants; if binding is too high, trogocytosis and T cell exhaustion can occur and many lower affinity CARs have been found to elicit good if not better tumor elimination (Olson et al, 2022; Mao et al, 2022; Ghorashian et al, 2019). **However, there is no universal optimal affinity range, as CAR activity also depends on antigen density, epitope location, receptor architecture, and assay conditions. Our results are consistent with functional activity from moderate- or low-affinity binders, but the limited number of designs with quantifiable binding does not establish that successful CD20 CARs universally require low-affinity binding.**"

In summary, we specified that competitors should design for moderate, rather than strong, affinity. However, the exact range of affinity required is not well-documented nor established by our results and may vary based on the CAR properties.

> *One limitation of the crowd-sourced approach - that the authors also highlight - is that many teams used very similar approaches. It might be interesting in future competitions to combine this crowdsourcing with more systematic evaluation of design parameters by one (or some) teams and/or encourage the use of a diversity of approaches.*

We agree that analyzing some teams in more systematic detail would be a valuable addition to the manuscript. We have noted this in the limitations and future work section:

Lines 506-510: "[…] in the future it may prove to be worthwhile to test designs in a more systematic manner or by encouraging a diversity of approaches."

> *"the rank ordering of the clearly successful versus failing designs is unlikely to change materially" Why is this?*

We thank the reviewer for raising this point. This statement was intended to describe the consistent separation between successful and unsuccessful designs across the functional assays, rather than to claim demonstrated stability across T-cell donors. Because we did not test multiple donors, we have removed the unsupported statement.

2nd Sep 2026

Manuscript Number: MSB-2026-13898R
Title: Validation and analysis of 12,000 AI-driven CAR-T designs in the *Bits to Binders* competition
Author: Clayton Kosonocky
Alex Abel
Aaron Feller
Amanda Cifuentes Rieffer
Phillip Woolley
Jakub Lála
Daryl Barth
Tynan Gardner
Stephen Ekker
Andrew Ellington
Wesley Wierson
Edward Marcotte

Dear Edward,

Thank you for sending us your revised manuscript. We have now heard back from the two reviewers who were asked to re-evaluate your study. Both reviewers are now satisfied with the modifications made. Before we can formally accept your manuscript, we would ask you to address the following editorial-level issues:

1. Please move the "Code Availability" text to the "Data Availability" section. The separate "Code Availability" heading should be removed. Please also provide the specific URL for the ERS29396515 dataset in the Data Availability statement.

2. Please place the "Disclosure and competing interests statement" section after the Acknowledgments section.

3. Please remove the Author contributions section from the manuscript.

4. Funding information: BioMADE (T-OC-A-04-0087) should be removed from the Comment box and added as a separate entry using the "More Funders" option in the online submission system. The following funders are acknowledged in the manuscript but have not been provided as separate entries in the online submission system: the University of Texas at Austin Graduate Continuing Research Fellowship, the Charles Smith, Jr. Graduate Fellowship in Molecular Biosciences, the President's PhD scholarship at Imperial College London, and the Stengl-Wyer Endowment to The University of Texas at Austin's College of Natural Sciences. Please add these funders as separate entries using the "More Funders" option.

5. Callouts for Figure 3G, Appendix Figure S10, Appendix Figures S11 and S12, Appendix Figures S23-S26, and Appendix Tables S1-S7 are missing from the manuscript. Please add the relevant callouts in the text.

6. Four EV tables are currently included in the manuscript. Please remove them from the manuscript and upload each table as a separate XLSX file using the file type "Expanded View Content." The legend for each EV table should be placed above the corresponding table.

7. Please provide a "standfirst text" in a separate DOCX file that summarizes the study in one or two sentences (approximately 250 characters, including spaces), along with three to four "bullet points" highlighting the main findings. Please refer to published papers for examples.

8. Please upload only one clean Appendix PDF without highlights, using the file type "Expanded View Content." The Word version should not be uploaded. Please also add page numbers for all listed items to the table of contents on the Appendix title page and use "Appendix Information" instead of "Supplementary Information." Please note this file will not be typeset.

10. Source data: Please remove the source data for Figure 1 and uncheck the corresponding entry in the source data checklist. The source data for EV figures should be uploaded as one (ZIP) file per figure. All source data files for the Appendix figures should be combined into a single ZIP file.

11. Please remove the ORCID IDs from the manuscript title page.

12. Please rename the "Methods and Protocols" section to "Methods."

13. Please address the following issues in the figure legends, as identified by our Data Editor:

- Please note that the exact p values are not provided in the legends of Figures 3B-E and 5C-H.
- Please note that the box plots need to be defined in terms of minima, maxima, centre, bounds of box and whiskers, and percentile in the legends of Figures 5C-H.
- Please note that information related to n is missing in the legend of Figure EV5.
- Please note that the error bars are not defined in the legend of Figure EV5.
- Please note that the measure of centre for the error bars needs to be defined in the legends of Figures EV2 and EV3.

Use the following link to submit your revised paper:

https://msb.msubmit.net/cgi-bin/main.plex

As a matter of course, please make sure that you have correctly followed the instructions for authors as given on the submission website.

Thank you for submitting this interesting paper to Molecular Systems Biology.

Kind regards,
Jingyi

Jingyi Hou, PhD
Senior Editor
Molecular Systems Biology

If you do choose to resubmit, please click on the link below to submit the revision online before 2nd Oct 2026.

https://msb.msubmit.net/cgi-bin/main.plex

*** PLEASE NOTE *** As part of the EMBO Press transparent editorial process initiative (see our Editorial at https://dx.doi.org/10.1038/msb.2010.72 , Molecular Systems Biology will publish online a Review Process File to accompany accepted manuscripts. When preparing your letter of response, please be aware that in the event of acceptance, your cover letter/point-by-point document will be included as part of this File, which will be available to the scientific community. More information about this initiative is available in our Instructions to Authors. If you have any questions about this initiative, please contact the editorial office (msb@embo.org).

Reviewer #1:
(no additional comment)

Reviewer #2:

Thank you to the authors for performing all the analyses requested, I think it make the results stronger and I am happy with the outcome.

All editorial and formatting issues were resolved by the authors.

9th Sep 2026
Manuscript number: MSB-2026-13898RR
Title: Validation and analysis of 12,000 AI-driven CAR-T designs in the *Bits to Binders* competition

Dear Edward,

Thank you again for sending us your revised manuscript. We are now satisfied with the modifications made and I am pleased to inform you that your paper has been accepted for publication.

Your manuscript will be processed for publication by EMBO Press. It will be copy edited and you will receive page proofs prior to publication. Please note that you will be contacted by Springer Nature Author Services to complete licensing and payment information.

You may qualify for financial assistance for your publication charges - either via a Springer Nature fully open access agreement or an EMBO initiative. Check your eligibility: https://link.springer.com/journal/44320/how-to-publish-with-us

Should you be planning a Press Release on your article, please notify the production team via this form in order to coordinate publication and release dates: https://forms.cloud.microsoft/e/eH4A8JVP6g

If you have any questions, please do not hesitate to contact the Editorial Office. Thank you for your contribution to Molecular Systems Biology.

Kind regards,
Jingyi

Jingyi Hou, PhD
Senior Editor
Molecular Systems Biology

>>> Please note that it is Molecular Systems Biology policy for the transcript of the editorial process (containing referee reports and your response letter) to be published as an online supplement to each paper. If you do NOT want this, you will need to inform the Editorial Office via email immediately. More information is available here: https://link.springer.com/partners/embo-press/editorial-policies#Peer%20review

EMBO Press Author Checklist

Corresponding Author Name: Edward Marcotte	
Journal Submitted to: Molecular Systems Biology	
Manuscript Number: MSB-2026-13898	

USEFUL LINKS FOR COMPLETING THIS FORM
The EMBO Journal - Author Guidelines
EMBO Reports - Author Guidelines
Molecular Systems Biology - Author Guidelines
EMBO Molecular Medicine - Author Guidelines

Reporting Checklist for Life Science Articles (updated January
This checklist is adapted from Materials Design Analysis Reporting (MDAR) Checklist for Authors. MDAR establishes a minimum set of requirements in transparent reporting in the life sciences (see Statement of Task: 10.31222/osf.io/9sm4x). Please follow the journal's guidelines in preparing your
Please note that a copy of this checklist will be published alongside your article.

Abridged guidelines for figures
1. Data
The data shown in figures should satisfy the following conditions:
- the data were obtained and processed according to the field's best practice and are presented to reflect the results of the experiments in an accurate and unbiased manner.
- ideally, figure panels should include only measurements that are directly comparable to each other and obtained with the same assay.
- plots include clearly labeled error bars for independent experiments and sample sizes. Unless justified, error bars should not be shown for technical
- if n<5, the individual data points from each experiment should be plotted. Any statistical test employed should be justified.
- Source Data should be included to report the data underlying figures according to the guidelines set out in the authorship guidelines on Data

2. Captions
Each figure caption should contain the following information, for each panel where they are relevant:
- a specification of the experimental system investigated (eg cell line, species name).
- the assay(s) and method(s) used to carry out the reported observations and measurements.
- an explicit mention of the biological and chemical entity(ies) that are being measured.
- an explicit mention of the biological and chemical entity(ies) that are altered/varied/perturbed in a controlled manner.
- the exact sample size (n) for each experimental group/condition, given as a number, not a range;
- a description of the sample collection allowing the reader to understand whether the samples represent technical or biological replicates (including how many animals, litters, cultures, etc.).
- a statement of how many times the experiment shown was independently replicated in the laboratory.
- definitions of statistical methods and measures:
  - common tests, such as t-test (please specify whether paired vs. unpaired), simple χ2 tests, Wilcoxon and Mann-Whitney tests, can be unambiguously identified by name only, but more complex techniques should be described in the methods section;
  - are tests one-sided or two-sided?
  - are there adjustments for multiple comparisons?
  - exact statistical test results, e.g., P values = x but not P values < x;
  - definition of 'center values' as median or average;
  - definition of error bars as s.d. or s.e.m.

Please complete ALL of the questions below.
Select "Not Applicable" only when the requested information is not relevant for your study.

Materials

Newly Created Materials	Information included in the manuscript?	In which section is the information available? (Reagents and Tools Table, Materials and Methods, Figures, Data Availability Section)
New materials and reagents need to be available; do any restrictions apply?	Not Applicable	Reagents and Tools Table, Materials and Methods

Antibodies	Information included in the manuscript?	In which section is the information available? (Reagents and Tools Table, Materials and Methods, Figures, Data Availability Section)
For **antibodies** provide the following information:<br>- Commercial antibodies: RRID (if possible) or supplier name, catalogue number and or/clone number<br>- Non-commercial: RRID or citation	Yes	Reagents and Tools Table, Materials and Methods

DNA and RNA sequences	Information included in the manuscript?	In which section is the information available? (Reagents and Tools Table, Materials and Methods, Figures, Data Availability Section)
**Short novel DNA or RNA including primers, probes**: provide the sequences.	Yes	Data Availability Section

Cell materials	Information included in the manuscript?	In which section is the information available? (Reagents and Tools Table, Materials and Methods, Figures, Data Availability Section)
**Cell lines:** Provide species information, strain. Provide accession number in repository **OR** supplier name, catalog number, clone number, and/**OR** RRID.	Yes	Reagents and Tools Table, Materials and Methods
**Primary cultures:** Provide species, strain, sex of origin, genetic modification status.	Yes	Materials and Methods
Report if the cell lines were recently **authenticated** (e.g., by STR profiling) and tested for mycoplasma contamination.	Yes	Materials and Methods

Experimental animals	Information included in the manuscript?	In which section is the information available? (Reagents and Tools Table, Materials and Methods, Figures, Data Availability Section)
**Laboratory animals or Model organisms:** Provide species, strain, sex, age, genetic modification status. Provide accession number in repository **OR** supplier name, catalog number, clone number, **OR** RRID.	Not Applicable	
**Animal observed in or captured from the field:** Provide species, sex, and age where possible.	Not Applicable	
Please detail **housing and husbandry conditions**.	Not Applicable	

Plants and microbes	Information included in the manuscript?	In which section is the information available? (Reagents and Tools Table, Materials and Methods, Figures, Data Availability Section)
**Plants:** provide species and strain, ecotype and cultivar where relevant, unique accession number if available, and source (including location for collected wild specimens).	Not Applicable	
**Microbes:** provide species and strain, unique accession number if available, and source.	Not Applicable	

Human research participants	Information included in the manuscript?	In which section is the information available? (Reagents and Tools Table, Materials and Methods, Figures, Data Availability Section)
If collected and within the bounds of privacy constraints report on age, sex and gender or ethnicity for all study participants.	Not Applicable	

Core facilities	Information included in the manuscript?	In which section is the information available? (Reagents and Tools Table, Materials and Methods, Figures, Data Availability Section)
If your work benefited from core facilities, was their service mentioned in the acknowledgments section?	Yes	Reagents and Tools Table, Acknowledgements

Design

Study protocol	Information included in the manuscript?	In which section is the information available? (Reagents and Tools Table, Materials and Methods, Figures, Data Availability Section)
If study protocol has been **pre-registered, provide DOI in the manuscript**. For clinical trials, provide the trial registration number **OR** cite DOI.	Not Applicable	
Report the **clinical trial registration number** (at ClinicalTrials.gov or equivalent), where applicable.	Not Applicable	

Laboratory protocol	Information included in the manuscript?	In which section is the information available? (Reagents and Tools Table, Materials and Methods, Figures, Data Availability Section)
Provide DOI OR other citation details if **external detailed step-by-step protocols** are available.	Not Applicable	

Experimental study design and statistics	Information included in the manuscript?	In which section is the information available? (Reagents and Tools Table, Materials and Methods, Figures, Data Availability Section)
Include a statement about **sample size** estimate even if no statistical methods were used.	Yes	Figure legends
Were any steps taken to minimize the effects of subjective bias when allocating animals/samples to treatment (e.g. **randomization procedure**)? If yes, have they been described?	Yes	Materials and Methods
Include a statement about **blinding** even if no blinding was done.	Not Applicable	Not applicable
Describe **inclusion/exclusion criteria** if samples or animals were excluded from the analysis. Were the criteria pre-established?<br><br>If sample or data points were omitted from analysis, report if this was due to attrition or intentional exclusion and provide justification.	Yes	Materials and Methods
For every figure, are **statistical tests** justified as appropriate? Do the data meet the assumptions of the tests (e.g., normal distribution)? Describe any methods used to assess it. Is there an estimate of variation within each group of data? **I**s the variance similar between the groups that are being statistically compared?	Yes	Figure legends, Materials and Methods

Sample definition and in-laboratory replication	Information included in the manuscript?	In which section is the information available? (Reagents and Tools Table, Materials and Methods, Figures, Data Availability Section)
In the figure legends: state number of times the experiment was **replicated** in laboratory.	Yes	Figure legends
In the figure legends: define whether data describe **technical or biological replicates**.	Yes	Figure legends

Ethics

Ethics	Information included in the manuscript?	In which section is the information available? (Reagents and Tools Table, Materials and Methods, Figures, Data Availability Section)
Studies involving **human participants**: State details of **authority granting ethics approval** (IRB or equivalent committee(s), provide reference number for approval.	Not Applicable	
Studies involving **human participants**: Include a statement confirming that **informed consent** was obtained from all subjects and that the experiments conformed to the principles set out in the WMA Declaration of Helsinki and the Department of Health and Human Services Belmont Report.	Not Applicable	
Studies involving **human participants:** For publication of **patient photos**, include a statement confirming that consent to publish was obtained.	Not Applicable	
Studies involving experimental **animals**: State details of **authority granting ethics approval** (IRB or equivalent committee(s), provide reference number for approval. Include a statement of compliance with ethical regulations.	Not Applicable	
Studies involving **specimen and field samples:** State if relevant **permits** obtained, provide details of authority approving study; if none were required, explain why.	Not Applicable	

Dual Use Research of Concern (DURC)	Information included in the manuscript?	In which section is the information available? (Reagents and Tools Table, Materials and Methods, Figures, Data Availability Section)
Could your study fall under dual use research restrictions? Please check biosecurity documents and list of **select agents and toxins** (CDC): https://www.selectagents.gov/sat/list.htm	Not Applicable	
If you used a select agent, is the security level of the lab appropriate and reported in the manuscript?	Not Applicable	
If a study is subject to dual use research of concern regulations, is the name of the **authority granting approval and reference number** for the regulatory approval provided in the manuscript?	Not Applicable	

Reporting

The MDAR framework recommends adoption of discipline-specific guidelines, established and endorsed through community initiatives. Journals have their own policy about requiring specific guidelines and recommendations to complement MDAR.

Adherence to community standards	Information included in the manuscript?	In which section is the information available? (Reagents and Tools Table, Materials and Methods, Figures, Data Availability Section)
State if relevant guidelines or checklists (e.g., **ICMJE, MIBBI, ARRIVE, PRISMA**) have been followed or provided.	Not Applicable	
For **tumor marker prognostic studies,** we recommend that you follow the **REMARK** reporting guidelines (see link list at top right). See author guidelines, under 'Reporting Guidelines'. Please confirm you have followed these guidelines.	Not Applicable	
For **phase II and III randomized controlled trials**, please refer to the **CONSORT** flow diagram (see link list at top right) and submit the CONSORT checklist (see link list at top right) with your submission. See author guidelines, under 'Reporting Guidelines'. Please confirm you have submitted this list.	Not Applicable	

Data Availability

Data availability	Information included in the manuscript?	In which section is the information available? (Reagents and Tools Table, Materials and Methods, Figures, Data Availability Section)
Have **primary datasets** been deposited according to the journal's guidelines (see 'Data Deposition' section) and the respective accession numbers provided in the Data Availability Section?	Yes	Data Availability Section
Were **human clinical and genomic datasets** deposited in a public access-controlled repository in accordance to ethical obligations to the patients and to the applicable consent agreement?	Not Applicable	
Are **computational models** that are central and integral to a study available without restrictions in a machine-readable form? Were the relevant accession numbers or links provided?	Yes	Data Availability Section
If publicly available data were reused, provide the respective **data citations in the reference list**.	Not Applicable	
