# Supplementary material for: Validation and analysis of 12,000 AI-driven CAR-T designs in the Bits to Binders competition
Source: Mol Syst Biol. 2026 Oct 8;22(11):1688–712. doi: 10.1038/s44320-026-00246-1 (PMC13728253; doi:10.1038/s44320-026-00246-1)
Supplement: Supplementary file 5 [file 44320_2026_246_MOESM11_ESM.zip › Figure EV1 (pt 1)/06-Aug-2025-Immunophenotyping Analysis-Panel 3 - Layout.pdf]

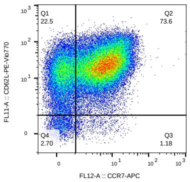

CAR28z-WT_Panel 3 CD45RA CD62L CCR7_2025-07-27.0001.mqd
VioBlue-A, SSC-A subset
69842

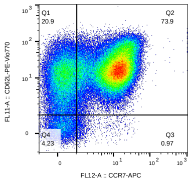

S2 Mock_Panel 3 CD45RA CD62L CCR7_2025-07-27.0001.mqd
VioBlue-A, SSC-A subset
158372

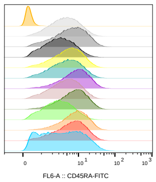

	Sample Name	Subset Name	Count
	CAR28z-WT_Panel 4 No mAbs_2025-07-27.0001.mqd	VioBlue-A, SSC-A subset	62632
	5981_Panel 3 CD45RA CD62L CCR7_2025-07-27.0001.mqd	VioBlue-A, SSC-A subset	51546
	5624_Panel 3 CD45RA CD62L CCR7_2025-07-27.0001.mqd	VioBlue-A, SSC-A subset	37786
	5300_Panel 3 CD45RA CD62L CCR7_2025-07-27.0001.mqd	VioBlue-A, SSC-A subset	51314
	3718_Panel 3 CD45RA CD62L CCR7_2025-07-27.0001.mqd	VioBlue-A, SSC-A subset	62959
	3494_Panel 3 CD45RA CD62L CCR7_2025-07-27.0001.mqd	VioBlue-A, SSC-A subset	44013
	3109_Panel 3 CD45RA CD62L CCR7_2025-07-27.0001.mqd	VioBlue-A, SSC-A subset	40318
	2383_Panel 3 CD45RA CD62L CCR7_2025-07-27.0001.mqd	VioBlue-A, SSC-A subset	68662
	1892_Panel 3 CD45RA CD62L CCR7_2025-07-27.0001.mqd	VioBlue-A, SSC-A subset	64199
	1506_Panel 3 CD45RA CD62L CCR7_2025-07-27.0001.mqd	VioBlue-A, SSC-A subset	82913
	1304_Panel 3 CD45RA CD62L CCR7_2025-07-27.0001.mqd	VioBlue-A, SSC-A subset	54331
	CAR28z-WT_Panel 3 CD45RA CD62L CCR7_2025-07-27.0001.mqd	VioBlue-A, SSC-A subset	69842
	S2 Mock_Panel 3 CD45RA CD62L CCR7_2025-07-27.0001.mqd	VioBlue-A, SSC-A subset	158372

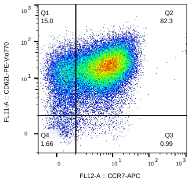

1304_Panel 3 CD45RA CD62L CCR7_2025-07-27.0001.mqd
VioBlue-A, SSC-A subset
54331

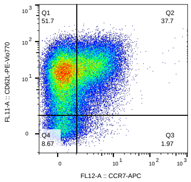

1506_Panel 3 CD45RA CD62L CCR7_2025-07-27.0001.mqd
VioBlue-A, SSC-A subset
82913

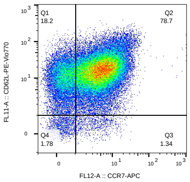

1892_Panel 3 CD45RA CD62L CCR7_2025-07-27.0001.mqd
VioBlue-A, SSC-A subset
64199

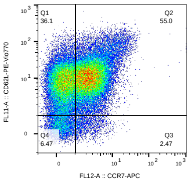

2383_Panel 3 CD45RA CD62L CCR7_2025-07-27.0001.mqd
VioBlue-A, SSC-A subset
68662

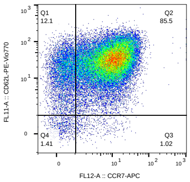

3109_Panel 3 CD45RA CD62L CCR7_2025-07-27.0001.mqd
VioBlue-A, SSC-A subset
40318

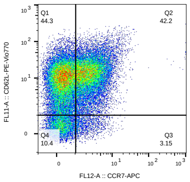

3494_Panel 3 CD45RA CD62L CCR7_2025-07-27.0001.mqd
VioBlue-A, SSC-A subset
44013

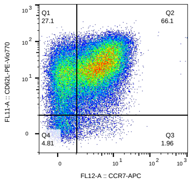

3718_Panel 3 CD45RA CD62L CCR7_2025-07-27.0001.mqd
VioBlue-A, SSC-A subset
62959

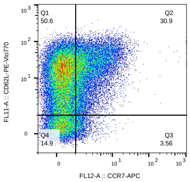

5300_Panel 3 CD45RA CD62L CCR7_2025-07-27.0001.mqd
VioBlue-A, SSC-A subset
51314

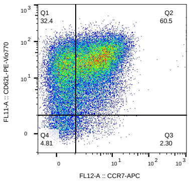

5624_Panel 3 CD45RA CD62L CCR7_2025-07-27.0001.mqd
VioBlue-A, SSC-A subset
37786

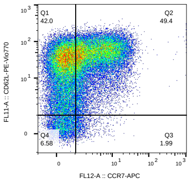

5981_Panel 3 CD45RA CD62L CCR7_2025-07-27.0001.mqd
VioBlue-A, SSC-A subset
51546

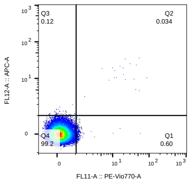

CAR28z-WT_Panel 4 No mAbs_2025-07-27.0001.mqd
VioBlue-A, SSC-A subset
62632
