# Supplementary material for: Validation and analysis of 12,000 AI-driven CAR-T designs in the Bits to Binders competition
Source: Mol Syst Biol. 2026 Oct 8;22(11):1688–712. doi: 10.1038/s44320-026-00246-1 (PMC13728253; doi:10.1038/s44320-026-00246-1)
Supplement: Supplementary file 5 [file 44320_2026_246_MOESM11_ESM.zip › Figure EV1 (pt 1)/06-Aug-2025-Immunophenotyping Analysis-Panel 2 - Layout.pdf]

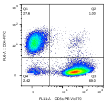

CAR28z-WT_Panel 2 CD4 CD8 PD1_2025-07-27.0001.mqd
VioBlue-A, SSC-A subset
81647

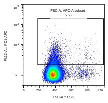

CAR28z-WT_Panel 2 CD4 CD8 PD1_2025-07-27.0001.mqd
VioBlue-A, SSC-A subset
81647

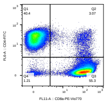

S2 Mock_Panel 2 CD4 CD8 PD1_2025-07-27.0001.mqd
VioBlue-A, SSC-A subset
157917

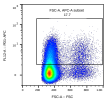

S2 Mock_Panel 2 CD4 CD8 PD1_2025-07-27.0001.mqd
VioBlue-A, SSC-A subset
157917

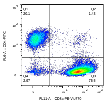

1304_Panel 2 CD4 CD8 PD1_2025-07-27.0001.mqd
VioBlue-A, SSC-A subset
70752

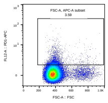

1304_Panel 2 CD4 CD8 PD1_2025-07-27.0001.mqd
VioBlue-A, SSC-A subset
70752

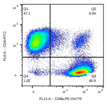

1506_Panel 2 CD4 CD8 PD1_2025-07-27.0001.mqd
VioBlue-A, SSC-A subset
83328

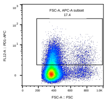

1506_Panel 2 CD4 CD8 PD1_2025-07-27.0001.mqd
VioBlue-A, SSC-A subset
83328

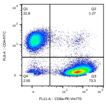

1892_Panel 2 CD4 CD8 PD1_2025-07-27.0001.mqd
VioBlue-A, SSC-A subset
75951

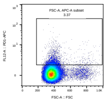

1892_Panel 2 CD4 CD8 PD1_2025-07-27.0001.mqd
VioBlue-A, SSC-A subset
75951

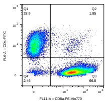

2383_Panel 2 CD4 CD8 PD1_2025-07-27.0001.mqd
VioBlue-A, SSC-A subset
67146

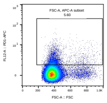

2383_Panel 2 CD4 CD8 PD1_2025-07-27.0001.mqd
VioBlue-A, SSC-A subset
67146

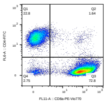

3109_Panel 2 CD4 CD8 PD1_2025-07-27.0001.mqd
VioBlue-A, SSC-A subset
52949

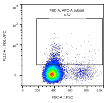

3109_Panel 2 CD4 CD8 PD1_2025-07-27.0001.mqd
VioBlue-A, SSC-A subset
52949

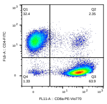

3494_Panel 2 CD4 CD8 PD1_2025-07-27.0001.mqd
VioBlue-A, SSC-A subset
60583

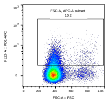

3494_Panel 2 CD4 CD8 PD1_2025-07-27.0001.mqd
VioBlue-A, SSC-A subset
60583

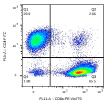

3718_Panel 2 CD4 CD8 PD1_2025-07-27.0001.mqd
VioBlue-A, SSC-A subset
69121

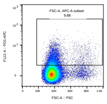

3718_Panel 2 CD4 CD8 PD1_2025-07-27.0001.mqd
VioBlue-A, SSC-A subset
69121

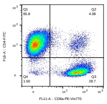

5300_Panel 2 CD4 CD8 PD1_2025-07-27.0001.mqd
VioBlue-A, SSC-A subset
52474

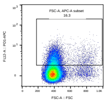

5300_Panel 2 CD4 CD8 PD1_2025-07-27.0001.mqd
VioBlue-A, SSC-A subset
52474

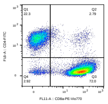

5624_Panel 2 CD4 CD8 PD1_2025-07-27.0001.mqd
VioBlue-A, SSC-A subset
38548

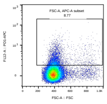

5624_Panel 2 CD4 CD8 PD1_2025-07-27.0001.mqd
VioBlue-A, SSC-A subset
38548

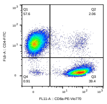

5981_Panel 2 CD4 CD8 PD1_2025-07-27.0001.mqd
VioBlue-A, SSC-A subset
49331

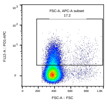

5981_Panel 2 CD4 CD8 PD1_2025-07-27.0001.mqd
VioBlue-A, SSC-A subset
49331

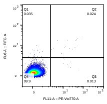

CAR28z-WT_Panel 4 No mAbs_2025-07-27.0001.mqd
VioBlue-A, SSC-A subset
62632

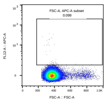

CAR28z-WT_Panel 4 No mAbs_2025-07-27.0001.mqd
VioBlue-A, SSC-A subset
62632
