# Supplementary material for: Validation and analysis of 12,000 AI-driven CAR-T designs in the Bits to Binders competition
Source: Mol Syst Biol. 2026 Oct 8;22(11):1688–712. doi: 10.1038/s44320-026-00246-1 (PMC13728253; doi:10.1038/s44320-026-00246-1)
Supplement: Supplementary file 5 [file 44320_2026_246_MOESM11_ESM.zip › Figure EV1 (pt 1)/06-Aug-2025-Immunophenotyping Analysis-Panel 1 - Layout.pdf]

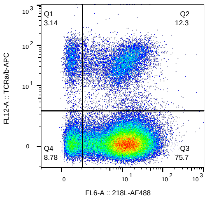

CAR28z-WT_Panel 1 CAR HLA TCR_2025-07-27.0002.mqd
VioBlue-A, SSC-A subset
68450

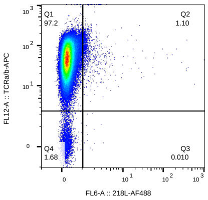

S2 Mock_Panel 1 CAR HLA TCR_2025-07-27.0001.mqd
VioBlue-A, SSC-A subset
136534

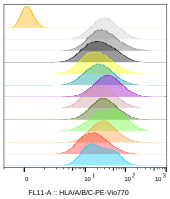

	Sample Name	Subset Name	Count	Geometric Mean : FL11-A
	CAR28z-WT_Panel 4 No mAbs_2025-07-27.0001.mqd	VioBlue-A, SSC-A subset	62632	0.20
	5981_Panel 1 CAR HLA TCR_2025-07-27.0001.mqd	VioBlue-A, SSC-A subset	47483	31.3
	5624_Panel 1 CAR HLA TCR_2025-07-27.0001.mqd	VioBlue-A, SSC-A subset	35798	28.9
	5300_Panel 1 CAR HLA TCR_2025-07-27.0001.mqd	VioBlue-A, SSC-A subset	47063	22.7
	3718_Panel 1 CAR HLA TCR_2025-07-27.0001.mqd	VioBlue-A, SSC-A subset	72861	19.7
	3494_Panel 1 CAR HLA TCR_2025-07-27.0001.mqd	VioBlue-A, SSC-A subset	60978	22.4
	3109_Panel 1 CAR HLA TCR_2025-07-27.0001.mqd	VioBlue-A, SSC-A subset	51464	37.1
	2383_Panel 1 CAR HLA TCR_2025-07-27.0001.mqd	VioBlue-A, SSC-A subset	67363	24.0
	1892_Panel 1 CAR HLA TCR_2025-07-27.0001.mqd	VioBlue-A, SSC-A subset	65112	29.9
	1506_Panel 1 CAR HLA TCR_2025-07-27.0001.mqd	VioBlue-A, SSC-A subset	78023	36.5
	1304_Panel 1 CAR HLA TCR_2025-07-27.0001.mqd	VioBlue-A, SSC-A subset	68997	28.7
	CAR28z-WT_Panel 1 CAR HLA TCR_2025-07-27.0002.mqd	VioBlue-A, SSC-A subset	68450	16.8
	S2 Mock_Panel 1 CAR HLA TCR_2025-07-27.0001.mqd	VioBlue-A, SSC-A subset	136534	22.7

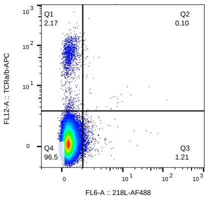

1304_Panel 1 CAR HLA TCR_2025-07-27.0001.mqd
VioBlue-A, SSC-A subset
68997

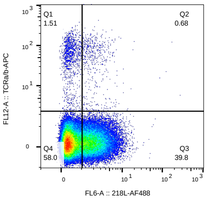

1506_Panel 1 CAR HLA TCR_2025-07-27.0001.mqd
VioBlue-A, SSC-A subset
78023

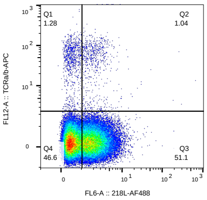

1892_Panel 1 CAR HLA TCR_2025-07-27.0001.mqd
VioBlue-A, SSC-A subset
65112

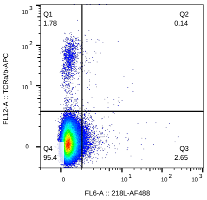

2383_Panel 1 CAR HLA TCR_2025-07-27.0001.mqd
VioBlue-A, SSC-A subset
67363

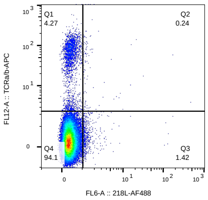

3109_Panel 1 CAR HLA TCR_2025-07-27.0001.mqd
VioBlue-A, SSC-A subset
51464

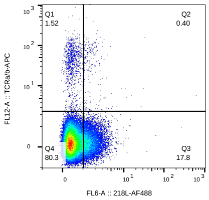

3494_Panel 1 CAR HLA TCR_2025-07-27.0001.mqd
VioBlue-A, SSC-A subset
60978

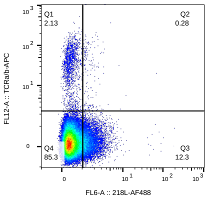

3718_Panel 1 CAR HLA TCR_2025-07-27.0001.mqd
VioBlue-A, SSC-A subset
72861

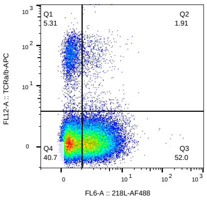

5300_Panel 1 CAR HLA TCR_2025-07-27.0001.mqd
VioBlue-A, SSC-A subset
47063

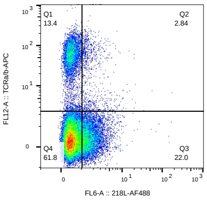

5624_Panel 1 CAR HLA TCR_2025-07-27.0001.mqd
VioBlue-A, SSC-A subset
35798

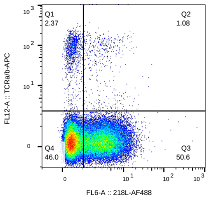

5981_Panel 1 CAR HLA TCR_2025-07-27.0001.mqd
VioBlue-A, SSC-A subset
47483

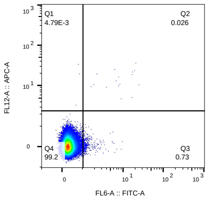

CAR28z-WT_Panel 4 No mAbs_2025-07-27.0001.mqd
VioBlue-A, SSC-A subset
62632
